# High-flow nasal cannula for pre- and apneic oxygenation during rapid sequence induction intubation in emergency surgery: A systematic review and meta-analysis

**DOI:** 10.1371/journal.pone.0316918

**Published:** 2025-01-24

**Authors:** Hong Tang, Yanyan Yang, Hong Li

**Affiliations:** Department of Anesthesiology, The Second Affiliated Hospital, The Army Military Medical University, Chongqing, China; Universidad de La Sabana, COLOMBIA

## Abstract

**Background:**

Rapid sequence induction intubation (RSII) is commonly used in emergency surgeries for patients at high risk of aspiration. However, these patients are more susceptible to hypoxemia during the RSII process. High-flow nasal cannula (HFNC) oxygen therapy has emerged as a potential alternative to traditional face mask (FM) ventilation pre- and apneic oxygenation. This meta-analysis aimed to evaluate the efficacy of HFNC compared to FM during RSII in emergency surgeries.

**Methods:**

We conducted a comprehensive literature search across PubMed-MEDLINE, EMBASE-OVID, Scopus, and Web of Science databases up to July 20, 2024. Randomized controlled trials comparing HFNC with FM during RSII for emergency surgery patients were included. The primary outcomes were post-intubation arterial partial pressures of oxygen (PaO_2_) and carbon dioxide (PaCO_2_). Secondary outcomes included post-intubation end-tidal carbon dioxide concentration (EtCO_2_), incidence of desaturation, apnea time, lowest peripheral oxygen saturation (Lowest SpO_2_), and occurrence of regurgitant aspiration. We used the GRADE approach to assess the certainty of evidence and the Cochrane Risk of Bias 2 (RoB 2) tool to evaluate risk of bias.

**Findings:**

This meta-analysis encompassed six studies, involving a total of 703 patients. HFNC oxygen therapy demonstrated a significant increase in post-intubation arterial PaO_2_ compared to FM (mean difference = 63.02 mmHg, 95% CI: 8.99 to 117.05, p = 0.02), while no significant difference was observed in arterial PaCO_2_. Moreover, HFNC substantially prolonged apnea time (mean difference = 19.25 seconds, 95% CI: 1.69 to 36.82, p = 0.03). No statistically significant differences were found between HFNC and FM regarding EtCO_2_, incidence of desaturation, Lowest SpO_2_, or regurgitant aspiration.

**Conclusion:**

This systematic review and meta-analysis indicates that HFNC may be superior to FM for pre-oxygenation and apneic oxygenation during RSII in emergency surgeries, particularly in improving oxygenation. While these findings are promising, further high-quality research is necessary to establish definitive guidelines for HFNC use in this context.

## Introduction

For patients with gastrointestinal obstruction, full stomach, or at high risk of regurgitant aspiration, rapid sequence induction intubation (RSII) is commonly employed to minimize the time interval between the loss of airway protective reflexes and successful tracheal intubation. Studies indicate that emergency surgery patients are not only more susceptible to hypoxemia [1], but also experience a high incidence of hypoxemia during the RSII process itself [1,2]. This dual risk underscores the importance of selecting effective pre- and apneic oxygenation methods for patients undergoing RSII in emergency surgeries.

In recent years, trans-nasal humidified rapid insufflation ventilatory exchange (THRIVE), also known as high-flow nasal cannula (HFNC) oxygen therapy, has gained attention. HFNC consists of an air/oxygen blender, an active humidifier, a single heated circuit, and a nasal cannula, capable of delivering a constant fraction of inspired oxygen (0.21–1.0) at high flow rates (up to 60 L·min^-1^ or higher) [3]. HFNC is widely used in intensive care unit (ICU) patients for the management of hypoxemic respiratory failure, owing to its straightforward setup, good patient tolerance, and demonstrated efficacy [4]. Studies have demonstrated several physiological advantages of HFNC, including generation of continuous positive airway pressure [5], reduction of anatomical dead space [6], improvement of ventilation-perfusion ratio [7], enhancement of mucociliary clearance [8], and reduction of work of breathing [7,9], among others.

Since its first application for pre- and apneic oxygenation during general anesthesia in 2015, HFNC has shown potential benefits in improving pre-oxygenation levels, enhancing oxygenation, and prolonging safe apnea time [10]. Although numerous clinical anesthesia studies have extensively discussed the perioperative application of HFNC, particularly its effects during anesthetic induction and apneic periods, many research findings remain controversial [11–13]. Currently, there is a lack of systematic reviews and meta-analyses on the effectiveness of HFNC application in RSII. Therefore, this meta-analysis aims to evaluate the efficacy of HFNC compared to traditional face mask (FM) ventilation in reducing the risk of hypoxemia during RSII for emergency surgeries by comprehensively analyzing existing randomized controlled trials (RCTs). We hope to provide more reliable evidence-based guidance for clinical practice and optimize oxygenation strategies during RSII through this study.

## Methods

The protocol for this systematic review and meta-analysis was prospectively registered in PROSPERO (International Prospective Register of Systematic Reviews; registration number: CRD42024575204). This study was conducted and reported in accordance with the Preferred Reporting Items for Systematic Reviews and Meta-Analyses (PRISMA) statement [14] (S1 Fig).

### Search strategy

A comprehensive literature search was performed in PubMed, Embase, Scopus, and Web of Science databases from inception through July 20, 2024. The full search strategy is detailed in the Supplementary materials. To ensure comprehensive coverage, a manual examination of the bibliographies of all included studies was conducted to identify any additional relevant articles not captured by the electronic search.

### Eligibility criteria

Inclusion criteria were: (1) comparing the effects of HFNC and FM during RSII for patients undergoing emergency surgery requiring general anesthesia;(2) RCTs; (3) participants aged over 16 years; (4) non-pregnant individuals. Literature exclusion criteria:(1) case reports, reviews, and other non-original literature were excluded; (2) studies with inaccessible full-text articles or insufficient data for analysis.

### Study selection and data extraction

Two independent reviewers (H.T. and Y.Y.Y.) systematically screened titles and abstracts after removing duplicate records, adhering to predetermined inclusion and exclusion criteria. Full-text articles of potentially eligible studies were subsequently retrieved and independently evaluated by the same reviewers. Any discrepancies arising during the screening process were resolved through consultation with a third reviewer (H.L.). From the final set of included studies, we extracted pertinent data, including the first author, publication year, country, sample size, and interventions in both the HFNC and FM groups. Primary outcomes encompassed post-intubation arterial partial pressures of oxygen (PaO_2_) and carbon dioxide (PaCO_2_). Secondary outcomes included post-intubation end-tidal carbon dioxide concentration (EtCO_2_), incidence of desaturation (defined as oxygen saturation <93%) from anesthesia induction to intubation, apnea time, lowest peripheral oxygen saturation (Lowest SpO_2_), occurrence of regurgitant aspiration. Apnea time was specifically defined as the period from the start of apnea until a carbon dioxide trace was visible on capnography.

### Risk of bias and quality assessment

We applied the Grading of Recommendations Assessment, Development and Evaluation (GRADE) [15] approach to assess the certainty of evidence, and utilized the Cochrane Risk of Bias 2 (RoB 2) [16] tool to evaluate risk of bias in the included studies. These assessments were conducted independently by two reviewers (H.T. and Y.Y.Y.), with any disagreements resolved through discussion or consultation with a third reviewer (H.L.) when necessary.

#### Statistical analysis

A meta-analysis was conducted using Review Manager (RevMan version 5.4.1), software developed by the Cochrane Collaboration. For categorical outcomes, the Mantel-Haenszel method was employed, with risk ratio (RR) as the effect measure. For continuous outcomes, the inverse variance method was utilized, with mean difference as the effect measure. Due to potential clinical heterogeneity among the included studies, a random-effects model was chosen for data pooling. Heterogeneity among studies was assessed using the I^2^ statistic. I^2^ values of 25%, 50%, and 75% were considered to indicate low, moderate, and high heterogeneity, respectively.

## Results

### Study selection and characteristics

**Fig 1:**
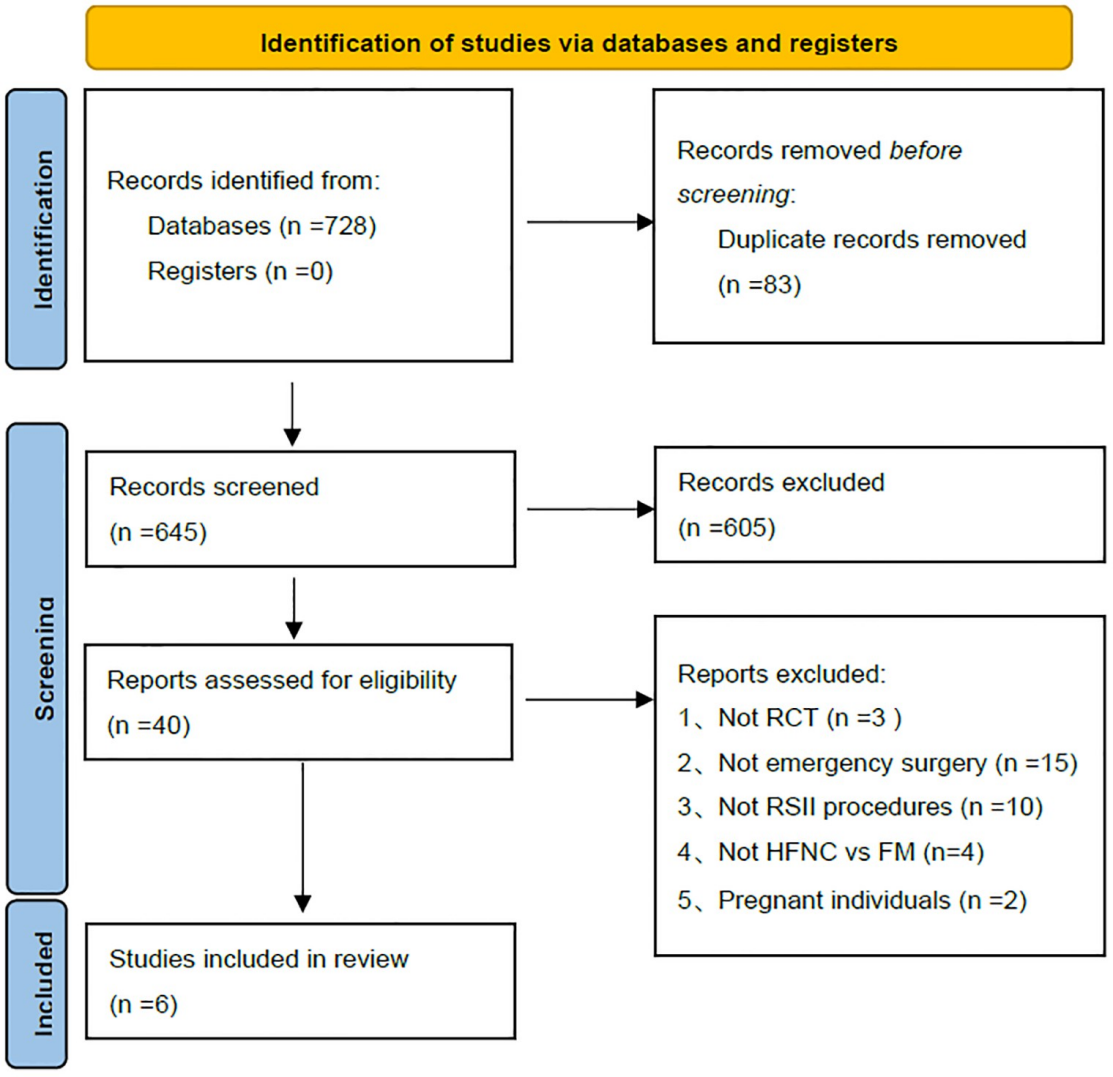
PRISMA-based flowchart for the selection of studies risk of bias and GRADE quality of outcomes.

Our initial literature search yielded 728 potentially relevant articles. After a rigorous screening process, six studies met our inclusion criteria and were selected for the meta-analysis. These studies collectively included 703 patients, with 352 allocated to the HFNC group and 351 to the FM group. The detailed study selection process is illustrated in Fig 1. Detailed information about the included studies is presented in S1 Table.

We conducted a comprehensive assessment of the risk of bias for all included studies and evaluated the quality of evidence for each outcome using the GRADE approach. The risk of bias assessment for individual studies is visually presented in S2 and S3 Figs. These figures provide a clear overview of the methodological quality across the included studies. To synthesize our findings and evaluate the overall quality of evidence, we performed a meta-analysis for each outcome and applied the GRADE methodology. S2 Table presents a comprehensive summary of these results, including effect estimates, confidence intervals, and the GRADE quality assessment for each outcome.

### Primary outcomes

**Fig 2:**
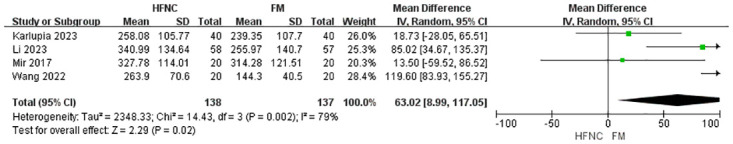
Forest plot of PaO_2_.

**Fig 3:**
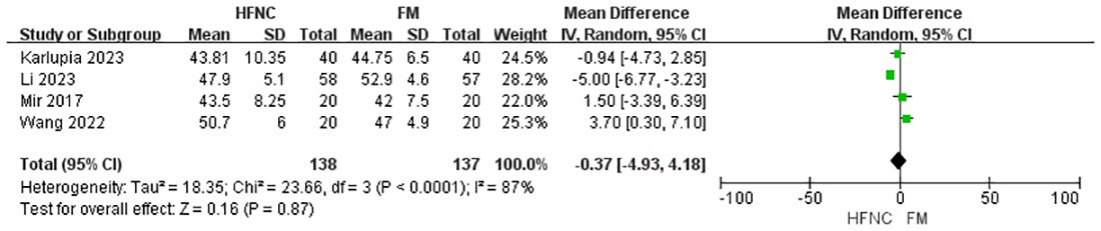
Forest plot of PaCO_2_.

Our meta-analysis focused on two primary outcomes: post-intubation PaO_2_ and PaCO_2_. For post-intubation PaO_2_, our analysis encompassed four studies with a total of 275 participants. The pooled effect estimate indicated a statistically significant increase in PaO_2_ favoring HFNC (MD = 63.02, 95% CI: 8.99 to 117.05, p = 0.02). However, high heterogeneity was detected among the studies (I^2^ = 79%) (Fig 2). For post-intubation PaCO_2_, our analysis encompassed four studies with a total of 275 participants. The analysis revealed no statistically significant difference between the HFNC and FM groups (MD = -0.37, 95% CI: -4.93 to 4.18, p = 0.87), with high heterogeneity also observed (I^2^ = 87%) (Fig 3).

### Secondary outcomes

**Fig 4:**
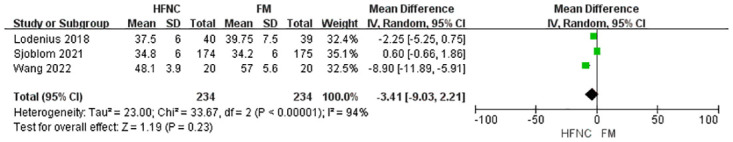
Forest plot of EtCO_2._

**Fig 5:**
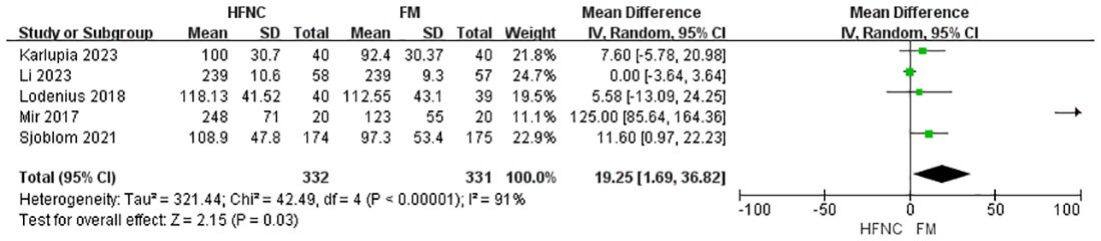
Forest plot of apnea time.

**Fig 6:**
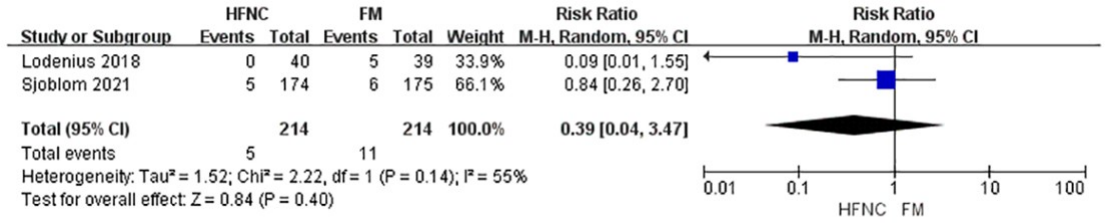
Forest plot of desaturation.

**Fig 7:**
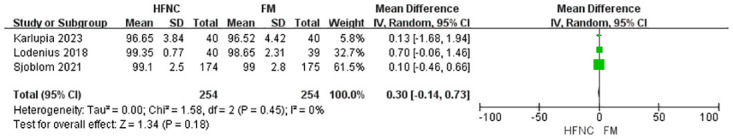
Forest plot of lowest SpO_2._

**Fig 8:**
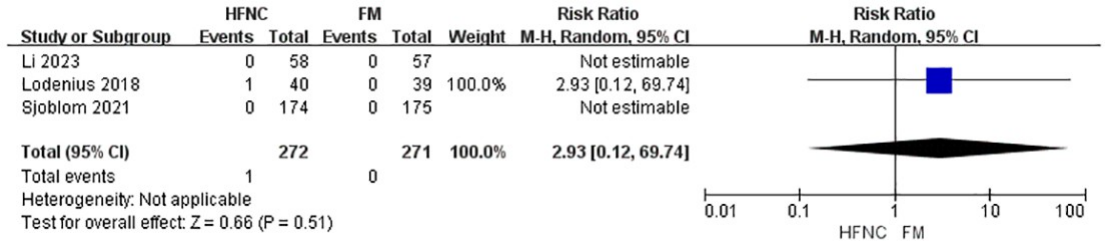
Forest plot of regurgitant aspiration.

The meta-analysis also evaluated several secondary outcomes. For post-intubation EtCO_2_, the analysis of three studies with 468 participants found no statistically significant difference between the HFNC and FM groups, although high heterogeneity was observed (MD = -3.41, 95% CI: -9.03 to 2.21, p = 0.23) (Fig 4). Regarding apnea time, the pooled analysis of five studies with 663 participants showed a statistically significant increase in apnea time favoring HFNC (MD = 19.25, 95% CI: 1.69 to 36.82, p = 0.03), again with high heterogeneity (I^2^ = 91%) (Fig 5). For oxygen saturation desaturation, the analysis of two studies with 428 participants found no significant difference between HFNC and FM (RR, 0.39; 95% CI, 0.04 to 3.57; p = 0.40), with moderate heterogeneity (I^2^ = 55%) (Fig 6). Similarly, the analysis of three studies with 508 participants revealed no significant difference in the lowest SpO_2_ between the two groups (MD = 0.30, 95% CI: -0.14 to 0.73, p = 0.18), with low heterogeneity (I^2^ = 0%) (Fig 7). Finally, the analysis of three studies with 543 participants found no significant difference in the incidence of regurgitant aspiration between HFNC and FM (MD = 2.92, 95% CI:0.12 to 69.74, p = 0.18), though the limited number of events prevented a robust assessment of heterogeneity (Fig 8).

## Discussion

Our meta-analysis of 6 studies encompassing 703 patients revealed that HFNC demonstrated superior oxygenation compared to FM during RSII, with significantly higher post-intubation PaO_2_ and longer apnea time. However, no significant differences were found in other outcomes including PaCO_2_, EtCO_2_, desaturation incidence, and regurgitant aspiration.

The primary outcome demonstrated significantly higher post-intubation PaO_2_ in the HFNC group compared to the FM group, suggesting superior oxygenation maintenance with HFNC. Notably, no significant difference was observed in post-intubation PaCO_2_ between the two groups, indicating that HFNC improved oxygenation without affecting carbon dioxide clearance. This enhanced oxygenation can be attributed to HFNC’s ability to provide a stable inspired oxygen concentration, generate distal positive airway pressure, increase end-expiratory lung volume and alveolar oxygen partial pressure, reduce intrapulmonary shunt, and decrease dead space ventilation due to its washout effect [5–8]. These physiological mechanisms may explain HFNC’s superior performance in increasing PaO_2_ compared to FM during pre-oxygenation.

Secondary outcomes showed no significant differences between HFNC and FM in EtCO_2_, incidence of desaturation, lowest SpO_2_, and regurgitant aspiration. These findings suggest that HFNC is comparable to traditional FM in maintaining these crucial physiological parameters. Of interest is the significantly prolonged apnea time observed with HFNC. Despite this extended apneic period, the HFNC group maintained higher post-intubation PaO_2_ levels, strongly indicating superior oxygenation during pre- and apneic oxygenation phases. Importantly, our analysis found no significant difference in the incidence of regurgitant aspiration between the two methods, a critical consideration for emergency surgery patients at higher risk of aspiration. This similar safety profile suggests that HFNC could be a viable alternative to FM without increasing aspiration risk. However, the wide confidence interval indicates considerable uncertainty in this estimate, likely due to the rarity of aspiration events in the included studies.

Our findings align with the meta-analysis by Song et al. [17], which did not restrict its focus to RSII, and with Bright et al. [18] study on obese patients. These consistencies across different patient populations and induction methods underscore HFNC’s potential for superior oxygenation maintenance during anesthesia induction in the operating room.

However, this study has several important limitations that warrant consideration. Firstly, the results are specifically applicable to patients undergoing RSII in emergency surgery, potentially limiting generalizability to other patient populations or clinical scenarios. This specificity is both a strength, as it focuses on a critical clinical context, and a limitation in terms of broader applicability. Secondly, the classical RSII technique emphasizes avoiding positive pressure ventilation before tracheal intubation [19], while modified RSII techniques recommend using pressure-limited positive pressure ventilation [20]. Regardless of the method used, these techniques inherently affect the oxygenation efficacy of the FM group during the induction to intubation phase, potentially biasing the comparison with HFNC. Furthermore, our meta-analysis is limited by high heterogeneity in some outcomes and a relatively small number of included studies. This heterogeneity might be attributed to variations in patient populations, pre-oxygenation times, and HFNC application methods across studies. The diversity in RSII techniques employed across different centers and studies further contributes to this heterogeneity, making direct comparisons challenging.

Future research should prioritize several key areas to advance our understanding and application of HFNC in RSII. Firstly, standardizing HFNC protocols for RSII is crucial to ensure consistency across studies and clinical practice. Secondly, investigating HFNC’s efficacy in specific patient subgroups, such as those with obesity or difficult airways, could provide valuable insights into its targeted applications. Thirdly, conducting larger, multicenter trials is essential to reduce heterogeneity and increase the robustness of findings.

Additionally, cost-effectiveness analyses comparing HFNC to FM would be valuable for informing clinical practice and healthcare resource allocation. These economic evaluations, coupled with clinical efficacy data, would provide a more comprehensive basis for decision-making in adopting HFNC for RSII in various healthcare settings.

## Conclusion

This systematic review and meta-analysis suggests that HFNC may offer advantages over FM for pre- and apneic oxygenation during RSII in emergency surgeries, particularly in terms of improved oxygenation. While these findings are promising, further high-quality research is needed to establish definitive guidelines for HFNC use in this setting.

## Supporting information

S1 Fig PRISMA checklist. (DOCX)

S2 Fig Risk of bias graph. (DOCX)

S3 Fig Risk of bias summary. (DOCX)

S1 Table Information about the included studies. (DOCX)

S2 Table GRADE quality assessment. (DOCX)

S1 File Search results. (DOCX)

S2 File Detailed content of GRADE quality assessment. (XLSX)

S3 File Details of literature selection. (XLSX)

S4 File Data extraction from included studies. (XLSX)

S5 File Search strategies. (DOCX)

S6 File (RIS)

S7 File (XLSM)

S8 File (XLSX)

S9 File (XLSX)

S10 File (RIS)

S11 File (CIW)

S12 File (NBIB)
