# Supplementary figures and images for: High-flow nasal cannula for pre- and apneic oxygenation during rapid sequence induction intubation in emergency surgery: A systematic review and meta-analysis
Source: PLoS One. 2025 Jan 24;20(1):e0316918. doi: 10.1371/journal.pone.0316918 (PMC11760591; doi:10.1371/journal.pone.0316918)

| 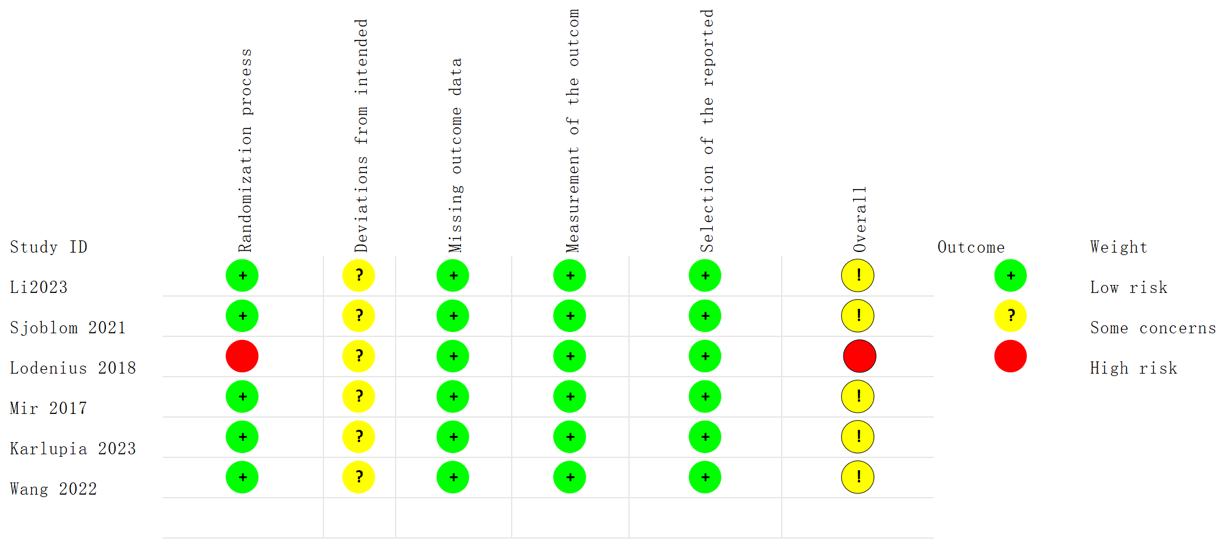 |
| --- |
| Figure 3 Risks of bias of individual studies |

Supplement: S2 Fig — (DOCX) [file pone.0316918.s002.docx]

| Table 3 Quality and certainty of evidence of included studies through the GRADE framework. |
| --- |
| 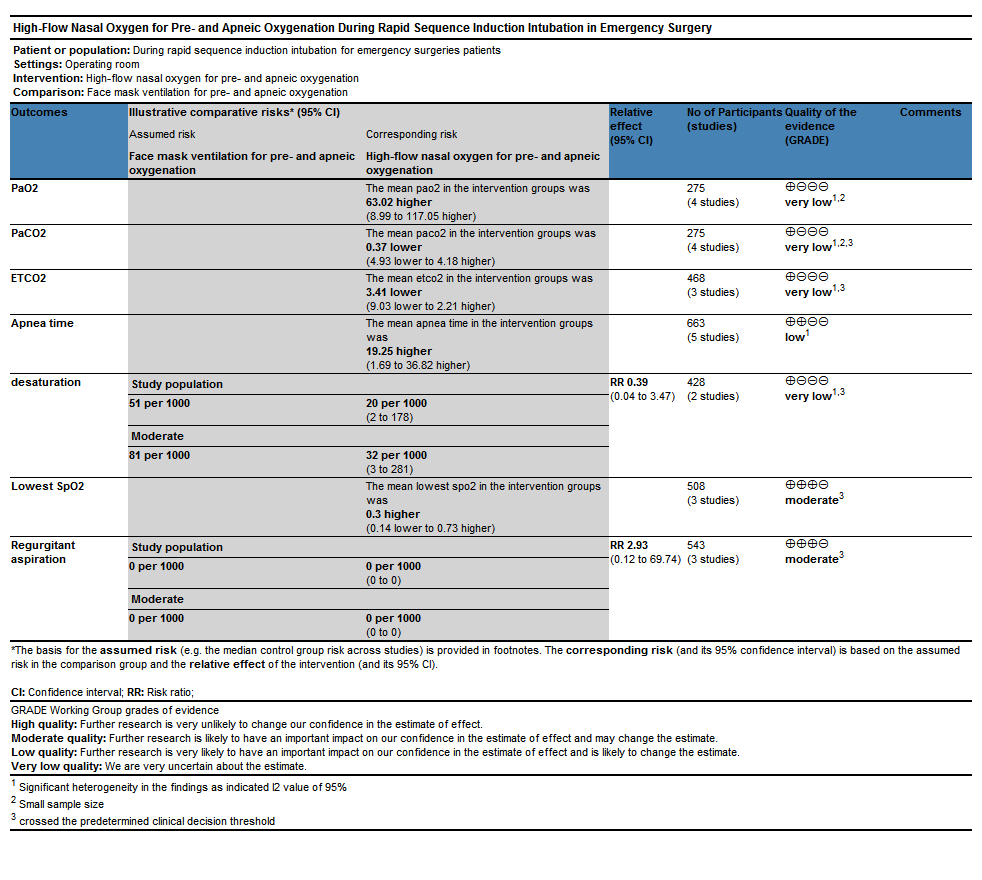 |
|  |

Supplement: S2 Table — (DOCX) [file pone.0316918.s005.docx]
