# Supplementary material for: High-flow nasal cannula for pre- and apneic oxygenation during rapid sequence induction intubation in emergency surgery: A systematic review and meta-analysis
Source: PLoS One. 2025 Jan 24;20(1):e0316918. doi: 10.1371/journal.pone.0316918 (PMC11760591; doi:10.1371/journal.pone.0316918)
Supplement: S3 Fig — (DOCX) [file pone.0316918.s003.docx]

Figure 3 Overall risks of bias of the six included studies
