# Supplementary material for: High-flow nasal cannula for pre- and apneic oxygenation during rapid sequence induction intubation in emergency surgery: A systematic review and meta-analysis
Source: PLoS One. 2025 Jan 24;20(1):e0316918. doi: 10.1371/journal.pone.0316918 (PMC11760591; doi:10.1371/journal.pone.0316918)
Supplement: S1 Table — (DOCX) [file pone.0316918.s004.docx]

| Table 2 Summary of study findings | | | | | | | | | | |
| --- | --- | --- | --- | --- | --- | --- | --- | --- | --- | --- |
| Author(year) | country | N(HFNC/FM) | Pre-o time | Induction-to-intubation time | HFNC | | | FM | | |
|  |  |  |  |  | Pre-o | Induction to intubation | During intubation | Pre-o | Induction to intubation | During intubation |
| Li et al (18)（ 2023） | China | 58/57 | 3min | 2.5 min | 30 L/min | 60 L/min | 60 L/min | 8 L/min | 8 L/min | Nil |
| Sjoblom et al(19)（2021） | Sweden | 174/175 | ≥3 min | Not mentioned | 30-50 L/min | 70 L/min | 70 L/min | 10L/min | 10L/min | Nil |
| Lodenius et al (13)（2018） | Sweden | 40/39 | ≥3 min | After NMB for 45-60 s | 40 L/min | 70 L/min | 70 L/min | 10L/min | 10L/min | Nil |
| Mir et al(20)（2017） | United Kingdom | 20/20 | 3min | After NMB for 60 s | 30- 70 L/min | 70 L/min | 70 L/min | 12L/min | 12L/min | Nil |
| Karlupia et al(21)(2023) | india | 40/40 | 3min | After NMB for 60 s | 60L/min | 60L/min | 60L/min | 12L/min | 12L/min | Nil |
| Wang et al(22)(2022) | china | 20/20 | 5 min | After NMB for 60 s | 40 L/min | FM ：6L/min | Nil | 6L/min | 6L/min | Nil |

Abbreviations: NMB, neuromuscular blocking;
