# Supplementary material for: High-flow nasal cannula for pre- and apneic oxygenation during rapid sequence induction intubation in emergency surgery: A systematic review and meta-analysis
Source: PLoS One. 2025 Jan 24;20(1):e0316918. doi: 10.1371/journal.pone.0316918 (PMC11760591; doi:10.1371/journal.pone.0316918)
Supplement: S1 File — (DOCX) [file pone.0316918.s006.docx]

Scopus:

1. Aroonpruksakul N, Sangsungnern P, Kiatchai T. Apneic oxygenation with low-flow oxygen cannula for rapid sequence induction and intubation in pediatric patients: a randomized-controlled trial. Translational Pediatrics. 2022;11(4):427-37. doi: 10.21037/tp-21-484.

2. Ayanmanesh F, Abdat R, Jurine A, Azale M, Rousseaux G, Coulons S, et al. Transnasal humidified rapid-insufflation ventilatory exchange during rapid sequence induction in children. Anaesthesia Critical Care and Pain Medicine. 2021;40(2). doi: 10.1016/j.accpm.2021.100817.

3. Bucher J, Koyfman A. Intubation of the neurologically injured patient. Journal of Emergency Medicine. 2015;49(6):920-7. doi: 10.1016/j.jemermed.2015.06.078.

4. Butterfield M, Bodnar D, Williamson F, Parker L, Ryan G. Prevalence of secondary insults and outcomes of patients with traumatic brain injury intubated in the prehospital setting: a retrospective cohort study. Emergency Medicine Journal. 2023;40(3):167-74. doi: 10.1136/emermed-2022-212513.

5. Chua MT, Khan FA, Ng WM, Lu Q, Low MJW, Yau YW, et al. Pre- and Apnoeic high flow oxygenation for RApid sequence intubation in the Emergency department (Pre-AeRATE): Study protocol for a multicentre, randomised controlled trial. Trials. 2019;20(1). doi: 10.1186/s13063-019-3305-8.

6. Chua MT, Ng WM, Lu Q, Low MJW, Punyadasa A, Cove ME, et al. Pre- and apnoeic high-flow oxygenation for rapid sequence intubation in the emergency department (the Pre-AeRATE trial): A multicentre randomised controlled trial. Annals of the Academy of Medicine, Singapore. 2022;51(3):149-60. doi: 10.47102/annals-acadmedsg.2021407.

7. Clrll MF, Akarca M, Unal Akoglu E, Cimilli Ozturk T, Onur Ö. High-Flow Nasal Cannula versus Bag Valve Mask for Preoxygenation during Rapid Sequence Intubation in the Emergency Department: A Single-Center, Prospective, Randomized Controlled Trial. Prehospital and Disaster Medicine. 2024;39(1):45-51. doi: 10.1017/S1049023X23006684.

8. Crewdson K, Heywoth A, Rehn M, Sadek S, Lockey D. Apnoeic oxygenation for emergency anaesthesia of pre-hospital trauma patients. Scandinavian Journal of Trauma, Resuscitation and Emergency Medicine. 2021;29(1). doi: 10.1186/s13049-020-00817-7.

9. de Lemos Carvalho VE, Couto TB, Moura BMH, Schvartsman C, Reis AG. Atropine does not prevent hypoxemia and bradycardia in tracheal intubation in the pediatric emergency department: observational study. Revista Paulista de Pediatria. 2023;42. doi: 10.1590/1984-0462/2024/42/2022220.

10. Gleason JM, Christian BR, Barton ED. Nasal cannula apneic oxygenation prevents desaturation during endotracheal intubation: An integrative literature review. Western Journal of Emergency Medicine. 2018;19(2):403-11. doi: 10.5811/westjem.2017.12.34699.

11. Grant S, Khan F, Keijzers G, Shirran M, Marneros L. Ventilator-assisted preoxygenation: Protocol for combining non-invasive ventilation and apnoeic oxygenation using a portable ventilator. EMA - Emergency Medicine Australasia. 2016;28(1):67-72. doi: 10.1111/1742-6723.12524.

12. Guitton C, Ehrmann S, Volteau C, Colin G, Maamar A, Jean-Michel V, et al. Nasal high-flow preoxygenation for endotracheal intubation in the critically ill patient: a randomized clinical trial. Intensive Care Medicine. 2019;45(4):447-58. doi: 10.1007/s00134-019-05529-w.

13. Hameed IZS, Hazra D, Ganesan P, Prabhakar AKP. Does apneic oxygenation with nasopharyngeal cannula during intubation improve the oxygenation in patients with acute hypoxemic respiratory failure compared to the standard bag valve mask preoxygenation? An open‑labeled randomized control trial. Turkish Journal of Emergency Medicine. 2024;24(1):33-40. doi: 10.4103/tjem.tjem_176_23.

14. Huda AU, Deabes AM. Perioperative anesthesia management of a pregnant patient with COVID-19 and Guillain-Barre syndrome undergoing emergency cesarean section – a case report. Anaesthesia, Pain and Intensive Care. 2021;25(3):395-8. doi: 10.35975/apic.v25i3.1517.

15. Jaber S, De Jong A, Schaefer MS, Zhang J, Ma X, Hao X, et al. Preoxygenation with standard facemask combining apnoeic oxygenation using high flow nasal cannula versuss standard facemask alone in patients with and without obesity: the OPTIMASK international study. Annals of Intensive Care. 2023;13(1). doi: 10.1186/s13613-023-01124-x.

16. Kulkarni KS, Dave N, Saran S, Garasia M, Parelkar S. Ultra-modified rapid sequence induction with transnasal humidified rapid insufflation ventilatory exchange: Challenging convention. Indian Journal of Anaesthesia. 2018;62(4):310-3. doi: 10.4103/ija.IJA_536_17.

17. Lodenius Å, Piehl J, Östlund A, Ullman J, Jonsson Fagerlund M. Transnasal humidified rapid-insufflation ventilatory exchange (THRIVE) vs. facemask breathing pre-oxygenation for rapid sequence induction in adults: a prospective randomised non-blinded clinical trial. Anaesthesia. 2018;73(5):564-71. doi: 10.1111/anae.14215.

18. Madhok J, Vogelsong MA, Lee TC, Wilson JG, Mihm F. Retrospective Analysis of Peri-Intubation Hypoxemia During the Coronavirus Disease 2019 Epidemic Using a Protocol for Modified Airway Management. A and A Practice. 2020;14(14):E01360. doi: 10.1213/XAA.0000000000001360.

19. McQuade D, Miller MR, Hayes-Bradley C. Addition of nasal cannula can either impair or enhance preoxygenation with a bag valve mask: A randomized crossover design study comparing oxygen flow rates. Anesthesia and Analgesia. 2018;126(4):1214-8. doi: 10.1213/ANE.0000000000002341.

20. Miller KA, Eisenberg MA, Abid ES, Nagler J. Use of the C-MAC Macintosh 0 Blade for Intubation of Infants in the Emergency Department. Pediatric Emergency Care. 2021;37(7):E404-E5. doi: 10.1097/PEC.0000000000001665.

21. Mir F, Patel A, Iqbal R, Cecconi M, Nouraei SAR. A randomised controlled trial comparing transnasal humidified rapid insufflation ventilatory exchange (THRIVE) pre-oxygenation with facemask pre-oxygenation in patients undergoing rapid sequence induction of anaesthesia. Anaesthesia. 2017;72(4):439-43. doi: 10.1111/anae.13799.

22. Mosier J, Reardon RF, DeVries PA, Stang JL, Nelsen A, Prekker ME, et al. Time to Loss of Preoxygenation in Emergency Department Patients. Journal of Emergency Medicine. 2020;59(5):637-42. doi: 10.1016/j.jemermed.2020.06.064.

23. Myatra SN, Ahmed SM, Kundra P, Garg R, Ramkumar V, Patwa A, et al. Republication: All India difficult airway association 2016 guidelines for tracheal intubation in the intensive care unit. Indian Journal of Critical Care Medicine. 2017;21(3):146-53. doi: 10.4103/ijccm.IJCCM_57_17.

24. Myatra SN, Ahmed SM, Kundra P, Garg R, Ramkumar V, Patwa A, et al. The all India difficult airway association 2016 guidelines for tracheal intubation in the intensive care unit. Indian Journal of Anaesthesia. 2016;60(12):922-30. doi: 10.4103/0019-5049.195485.

25. Myatra SN, Dhawan I, D Souza SA, Elakkumanan LB, Jain D, Natarajan P. Recent advances in airway management. Indian Journal of Anaesthesia. 2023;67(1):48-55. doi: 10.4103/ija.ija_26_23.

26. Ng I, Krieser R, Mezzavia P, Lee K, Tseng C, Douglas NWR, et al. The Use of Transnasal Humidified Rapid-Insufflation Ventilatory Exchange (THRIVE) for Pre-Oxygenation in Neurosurgical Patients: A Randomised Controlled Trial. Anaesthesia and Intensive Care. 2018;46(4):360-7. doi: 10.1177/0310057X1804600403.

27. Overmann KM, Boyd SD, Zhang Y, Kerrey BT. Apneic oxygenation to prevent oxyhemoglobin desaturation during rapid sequence intubation in a pediatric emergency department. American Journal of Emergency Medicine. 2019;37(8):1416-21. doi: 10.1016/j.ajem.2018.10.030.

28. Patwa A, Shah A, Garg R, Divatia J, Kundra P, Doctor JR, et al. All India difficult airway association (AIDAA) consensus guidelines for airway management in the operating room during the COVID-19 pandemic. Indian Journal of Anaesthesia. 2020;64(14):S107-S15. doi: 10.4103/ija.IJA_498_20.

29. Perera A, Alkouri H, Fogg T, Vassiliadis J, MacKenzie J, Wimalasena Y. Apnoeic oxygenation was associated with decreased desaturation rates during rapid sequence intubation in multiple Australian and New Zealand emergency departments. Emergency Medicine Journal. 2021;38(2):118-24. doi: 10.1136/emermed-2019-208424.

30. Pillai A, Chikhani M, Hardman JG. Apnoeic oxygenation in pregnancy: a modelling investigation. Anaesthesia. 2016;71(9):1077-80. doi: 10.1111/anae.13563.

31. Raineri SM, Cortegiani A, Accurso G, Procaccianti C, Vitale F, Caruso S, et al. Efficacy and safety of using high-flow nasal oxygenation in patients undergoing rapid sequence intubation. Turk Anesteziyoloji ve Reanimasyon Dernegi Dergisi. 2017;45(6):335-9. doi: 10.5152/TJAR.2017.47048.

32. Ramkumar V, Dinesh E, Shetty SR, Shah A, Kundra P, Das S, et al. All India difficult airway association 2016 guidelines for the management of unanticipated difficult tracheal intubation in obstetrics. Indian Journal of Anaesthesia. 2016;60(12):899-905. doi: 10.4103/0019-5049.195482.

33. Riddell A. The Effect of Apneic Oxygenation on Reducing Hypoxemia during Rapid Sequence Induction and Intubation in the Acutely Ill or Injured. Advanced Emergency Nursing Journal. 2017;39(4):309-17. doi: 10.1097/TME.0000000000000168.

34. Riyapan S, Lubin J. Apneic Oxygenation May Not Prevent Severe Hypoxemia During Rapid Sequence Intubation: A Retrospective Helicopter Emergency Medical Service Study. Air Medical Journal. 2016;35(6):365-8. doi: 10.1016/j.amj.2016.07.008.

35. Sakles JC, Mosier JM, Patanwala AE, Arcaris B, Dicken JM, Reardon RF. First Pass Success Without Hypoxemia is Increased with the Use of Apneic Oxygenation during Rapid Sequence Intubation in the Emergency Department. Academic Emergency Medicine. 2016;23(6):703-10. doi: 10.1111/acem.12931.

36. Scoccimarro A, West JR, Kanter M, Caputo ND. Waveform capnography: An alternative to physician gestalt in determining optimal intubating conditions after administration of paralytic agents. Emergency Medicine Journal. 2018;35(1):62-4. doi: 10.1136/emermed-2017-206922.

37. Sjöblom A, Broms J, Hedberg M, Lodenius Å, Furubacke A, Henningsson R, et al. Pre-oxygenation using high-flow nasal oxygen vs. tight facemask during rapid sequence induction. Anaesthesia. 2021;76(9):1176-83. doi: 10.1111/anae.15426.

38. Sjöblom A, Hedberg M, Gille A, Guerra A, Aanesen V, Forsberg IM, et al. Pre-oxygenation using high-flow nasal oxygen versus tight facemask in trauma patients undergoing emergency anaesthesia. Acta Anaesthesiologica Scandinavica. 2024;68(4):447-56. doi: 10.1111/aas.14368.

39. Sjöblom A, Hedberg M, Johansson S, Henningsson R, Soumpasis I, Lafrenz H, et al. Pre-oxygenation using high-flow nasal oxygen in parturients undergoing caesarean section in general anaesthesia: A prospective, multi-centre, pilot study. Acta Anaesthesiologica Scandinavica. 2023;67(8):1028-36. doi: 10.1111/aas.14264.

40. Stampfl M, Tillman D, Borelli N, Bandara T, Cathers A. Rapid Sequence Intubation Using the SEADUC Manual Suction Unit in a Contaminated Airway. Air Medical Journal. 2023;42(4):296-9. doi: 10.1016/j.amj.2023.03.007.

41. Vourc'H M, Huard D, Feuillet F, Baud G, Guichoux A, Surbled M, et al. Preoxygenation in difficult airway management: High-flow oxygenation by nasal cannula versus face mask (the PREOPTIDAM study). Protocol for a single-centre randomised study. BMJ Open. 2019;9(4). doi: 10.1136/bmjopen-2018-025909.

42. Waheed S, Kapadia NN, Khan MF, Kerai SM, Raheem A, Naeem R. Randomised controlled trial to assess the effectiveness of apnoeic oxygenation in adults using low-flow or high-flow nasal cannula with head side elevation versus usual care to prevent desaturation during endotracheal intubation in the emergency department (ApOxED): Study protocol. BMJ Open. 2020;10(11). doi: 10.1136/bmjopen-2020-037964.

43. Wang W, Zhang W, Lu Y, Xu Y, Zhang Y, Shi H, et al. Comparison of the Effectiveness of Transnasal Humidified Rapid Insufflation Ventilator Exchange (THRIVE) with Facemask Pre-Oxygenation in 40 Patients 365 Years of Age Undergoing General Anaesthesia During Gastrointestinal Surgery for Intestinal Obstruction. Medical Science Monitor. 2022;28. doi: 10.12659/MSM.938168.

44. Wimalasena Y, Burns B, Reid C, Ware S, Habig K. Apneic oxygenation was associated with decreased desaturation rates during rapid sequence intubation by an australian helicopter emergency medicine service. Annals of Emergency Medicine. 2015;65(4):371-6. doi: 10.1016/j.annemergmed.2014.11.014.

45. Wu YM, Li CC, Huang SY, Su YH, Wang CW, Chen JT, et al. A Comparison of Oxygenation Efficacy between High-Flow Nasal Cannulas and Standard Facemasks during Elective Tracheal Intubation for Patients with Obesity: A Randomized Controlled Trial. Journal of Clinical Medicine. 2022;11(6). doi: 10.3390/jcm11061700.

46. Ynineb Y, Boglietto E, Bonnet F, Quesnel C, Garnier M. Face-To-Face Double-Lumen Tube Intubation With the Airtraq Video Laryngoscope for Emergency Thoracic Surgery: A Case Report. Seminars in Cardiothoracic and Vascular Anesthesia. 2022;26(1):90-4. doi: 10.1177/10892532211007664.

Embase:

1. Abstracts of Posters Presented at the 2016 Annual Meeting of the International Anesthesia Research Society. Anesthesia and Analgesia. 2016;122(5).

2. 2019 Oncologic Emergency Medicine Conference. Journal of Emergency Medicine. 2019;57(3):e65-e98. doi: 10.1016/j.jemermed.2019.05.032.

3. World Airway Management Meeting. British Journal of Anaesthesia. 2020;125(1):e1-e216. doi: 10.1016/j.bja.2019.12.001.

4. Hydrocortisone for severe community acquired pneumonia in ICU. Drug and Therapeutics Bulletin. 2023;62(1):3. doi: 10.1136/dtb.2023.000057.

5. Adi O, Kai Fei S, Azma Haryaty A, Mohamed Sakan M. Preliminary report: A randomized controlled trial comparing helmet continuous positive airway pressure (CPAP) vs high flow nasal cannula (HFNC) for treatment of acute cardiogenic pulmonary oedema in the emergency department. Critical Care. 2019;23. doi: 10.1186/s13054-019-2358-0.

6. Agmy G, Adam M, Hsanen E, Mahmoud MA. High-flow nasal cannula versus noninvasive ventilation in the prevention of escalation to invasive mechanical ventilation in patients with acute hypoxemic respiratory failure. Egyptian Journal of Chest Diseases and Tuberculosis. 2022;71(1):81-7. doi: 10.4103/ecdt.ecdt_12_20.

7. Agmy GR, Adam M, Elkhawaga M, Hsanen E. High-flow nasal cannula versus noninvasive ventilation in the prevention of escalation to invasive mechanical ventilation in patients with acute hypoxemic respiratory failure. European Respiratory Journal. 2022;60. doi: 10.1183/13993003.congress-2022.4700.

8. Akbarian-rad Z, Mohammadi A, Khafri S, Ahmadpour-kacho M, Zahed-pasha Y, Haghshenas-Mojaveri M. Comparison of heated humidified high flow nasal cannula and nasal continuous positive airway pressure after surfactant administration in preterm neonates with respiratory distress syndrome. Clinical Respiratory Journal. 2020;14(8):740-7. doi: 10.1111/crj.13191.

9. Al Hashim AH, Al Reesi A, Al Lawati NM, Burad J, Al Khabori M, Chandwani J, et al. Comparison of Noninvasive Mechanical Ventilation With High-Flow Nasal Cannula, Face-Mask, and Helmet in Hypoxemic Respiratory Failure in Patients With COVID-19: A Randomized Controlled Trial∗. Critical Care Medicine. 2023;51(11):1515-26. doi: 10.1097/CCM.0000000000005963.

10. Alcock A, Thompson C, Robertson R, Ali T. An examination of the safety and efficacy of high flow nasal cannula therapy for acute severe asthma and viral induced wheeze in paediatric critical care. Archives of Disease in Childhood. 2016;101:A296-A8. doi: 10.1136/archdischild-2016-310863.487.

11. Aldawood B, Al-Rajhi A, Shahin J. High-flow nasal cannula in post-extubation patients: A meta-analysis. Critical Care Medicine. 2016;44(12):322. doi: 10.1097/01.ccm.0000509661.67290.5c.

12. Alfredo CA, Noemí CR, Samuel RL, Daniel OC, Rodrigo RB, Paul MT, et al. Effect of Norelgestromin and Ethinylestradiol in Transdermal Patches on the Clinical Outcomes and Biochemical Parameters of COVID-19 Patients: A Clinical Trial Pilot Study. Pharmaceuticals. 2022;15(6). doi: 10.3390/ph15060757.

13. Algamdi M, Ball I. High flow nasal cannula oxygen therapy for acute hypoxemic respiratory failure: A systematic review. Chest. 2016;150(4):306A. doi: 10.1016/j.chest.2016.08.319.

14. Amaru P, Delannoy B, Genty T, Desebbe O, Laverdure F, Rezaiguia-Delclaux S, et al. Effect of recruitment maneuvers and PEEP on respiratory failure after cardiothoracic surgery in obese subjects: A randomized controlled trial. Respiratory Care. 2021;66(8):1306-14. doi: 10.4187/RESPCARE.08607.

15. Andino R, Vega G, Pacheco SK, Arevalillo N, Leal A, Fernández L, et al. High-flow nasal oxygen reduces endotracheal intubation: a randomized clinical trial. Therapeutic Advances in Respiratory Disease. 2020;14. doi: 10.1177/1753466620956459.

16. Andreu M, Bertozzi M, Bezzi M, Borello S, Castro D, Di Giorgio V, et al. Comparison of Two Extubation Techniques in Critically Ill Adult Subjects: The ExtubAR Randomized Clinical Trial. Respiratory Care. 2022;67(1):76-86. doi: 10.4187/respcare.09276.

17. Andrews McArthur E, Saroha V. Retrospective consent for neonatal intubations. Going with the flow? Journal of Perinatology. 2023;43(10):1330-3. doi: 10.1038/s41372-023-01758-6.

18. Arabi YM, Aldekhyl S, Al Qahtani S, Al-Dorzi HM, Abdukahil SA, Al Harbi MK, et al. Effect of Helmet Noninvasive Ventilation vs Usual Respiratory Support on Mortality among Patients with Acute Hypoxemic Respiratory Failure Due to COVID-19: The HELMET-COVID Randomized Clinical Trial. JAMA. 2022;328(11):1063-72. doi: 10.1001/jama.2022.15599.

19. Arman PD, Varn MN, Povian S, Davis A, Uchakin P, Bhar A, et al. Effects of direct extubation to high-flow nasal cannula compared to standard nasal cannula in patients in the intensive care unit. American Journal of Respiratory and Critical Care Medicine. 2017;195. doi: 10.1164/ajrccm-conference.2017.A53.

20. Armanian AM, Iranpour R, Parvaneh M, Salehimehr N, Feizi A, Hajirezaei M. Heated Humidified High Flow Nasal Cannula (HHHFNC) is not an effective method for initial treatment of Respiratory Distress Syndrome (RDS) versus nasal intermittent mandatory ventilation (NIMV) and nasal continuous positive airway pressure (NCPAP). Journal of Research in Medical Sciences. 2019;24(1). doi: 10.4103/jrms.JRMS_2_19.

21. Asad H, Berkowitz D, Jaber W, Niroula A, Collins J. UTILIZATION OF HIGH FLOW NASAL CANNULA DURING RIGID BRONCHOSCOPIES AS A MODE OF VENTILATION. Chest. 2023;164(4):A5229. doi: 10.1016/j.chest.2023.07.3390.

22. Atallah J, Archambault D, Randall JD, Shepro A, Styskal LE, Glenn DR, et al. Rapid Quantum Magnetic IL-6 Point-of-Care Assay in Patients Hospitalized with COVID-19. Diagnostics. 2022;12(5). doi: 10.3390/diagnostics12051164.

23. Azoulay E, Lemiale V, Mokart D, Nseir S, Argaud L, Pène F, et al. Effect of High-Flow Nasal Oxygen vs Standard Oxygen on 28-Day Mortality in Immunocompromised Patients with Acute Respiratory Failure: The HIGH Randomized Clinical Trial. JAMA - Journal of the American Medical Association. 2018;320(20):2099-107. doi: 10.1001/jama.2018.14282.

24. Azoulay E, Lemiale V, Mokart D, Nseir S, Argaud L, Pène F, et al. High-flow nasal oxygen vs. standard oxygen therapy in immunocompromised patients with acute respiratory failure: Study protocol for a randomized controlled trial. Trials. 2018;19(1). doi: 10.1186/s13063-018-2492-z.

25. Bailly A, Boisramé-Helms J, Champigneulle B, Kamel T, Mercier E, Ricard JD, et al. Preoxygenation for intubation in the ICU: An ancillary study of the MACMAN Trial. Annals of Intensive Care. 2017;7(1):149-50. doi: 10.1186/s13613-016-0224-7.

26. Bailly A, Ricard JD, Le Thuaut A, Helms J, Kamel T, Mercier E, et al. Compared Efficacy of Four Preoxygenation Methods for Intubation in the ICU: Retrospective Analysis of McGrath Mac Videolaryngoscope Versus Macintosh Laryngoscope (MACMAN) Trial Data. Critical Care Medicine. 2019;47(4):E340-E8. doi: 10.1097/CCM.0000000000003656.

27. Baker EH, Patel K, Ball J, Edwards S, Harrison TS, Kaul A, et al. Insights from compassionate use of tocilizumab for COVID-19 to inform appropriate design of randomised controlled trials. British Journal of Clinical Pharmacology. 2021;87(3):1584-6. doi: 10.1111/bcp.14466.

28. Beran A, Srour O, Malhas SE, Mhanna M, Ayesh H, Sajdeya O, et al. High-Flow Nasal Cannula Versus Noninvasive Ventilation in Patients With COVID-19. Respiratory Care. 2022;67(9):1177-89. doi: 10.4187/respcare.09987.

29. Bernardo J, Makker K. Is aerosolized calfactant effective and safe in the treatment of respiratory distress syndrome? Journal of Perinatology. 2022;42(4):540-3. doi: 10.1038/s41372-022-01321-9.

30. Bikdeli B, Talasaz AH, Sharif-Kashani B, Rashidi F, Beigmohammadi MT, Moghadam KG, et al. Atorvastatin versus placebo in patients with covid-19 in intensive care: randomized controlled trial. The BMJ. 2022;376. doi: 10.1136/bmj-2021-068407.

31. Bonifácio LP, Ramacciotti E, Agati LB, Vilar FC, da Silva ACT, Júnior PL, et al. Efficacy and safety of Ixekizumab vs. low-dose IL-2 vs. Colchicine vs. standard of care in the treatment of patients hospitalized with moderate-to-critical COVID-19: A pilot randomized clinical trial (STRUCK: Survival Trial Using Cytokine Inhibitors). Revista da Sociedade Brasileira de Medicina Tropical. 2023;56. doi: 10.1590/0037-8682-0565-2022.

32. Borgi A, Louati A, Ghali N, Hajji A, Ayari A, Bouziri A, et al. High flow nasal cannula therapy versus continuous positive airway pressure and nasal positive pressure ventilation in infants with severe bronchiolitis: A randomized controlled trial. Pan African Medical Journal. 2021;40. doi: 10.11604/pamj.2021.40.133.30350.

33. Bräunlich J, Köppe-Bauernfeind N, Petroff D, Franke A, Wirtz H. Nasal high-flow compared to non-invasive ventilation in treatment of acute acidotic hypercapnic exacerbation of chronic obstructive pulmonary disease—protocol for a randomized controlled noninferiority trial (ELVIS). Trials. 2022;23(1). doi: 10.1186/s13063-021-05978-z.

34. Brugiere B, Frat JP, Petitpas F, Goudet V, Robert R, Debaene B. High flow oxygen therapy associated with noninvasive ventilation in hypoxemic acute respiratory failure: A prospective cohort study. European Journal of Anaesthesiology. 2012;29:6.

35. Cai Q, Ma W, Wu C, Liu H, Wang S, Zhang G. Is pre-oxygenation with high-flow nasal oxygen safe? randomized control trial of 56 cases of elderly patients during induction of general anesthesia with endotracheal intubation. Nan fang yi ke da xue xue bao = Journal of Southern Medical University. 2022;42(7):1069-74. doi: 10.12122/j.issn.1673-4254.2022.07.16.

36. Campbell DM, Shah PS, Shah V, Kelly EN. Nasal continuous positive airway pressure from high flow cannula versus Infant Flow for preterm infants. Journal of Perinatology. 2006;26(9):546-9. doi: 10.1038/sj.jp.7211561.

37. Carlesso E, Mauri T, Spinelli E, Galazzi A, Binda F, Tortolani D, et al. Effects of set flow rate on the ROX index in acute hypoxemic respiratory failure patients undergoing high flow therapy. Intensive Care Medicine Experimental. 2018;6. doi: 10.1186/s40635-018-0201-6.

38. Carrié C, Rieu B, Benard A, Trin K, Petit L, Massri A, et al. Early non-invasive ventilation and high-flow nasal oxygen therapy for preventing endotracheal intubation in hypoxemic blunt chest trauma patients: the OptiTHO randomized trial. Critical Care. 2023;27(1). doi: 10.1186/s13054-023-04429-2.

39. Caruso T, Tsui B, Wang E, Darling C, Sidell D. The incidence of desaturation during microlaryngoscopy and bronchoscopy: A quality control review. Anesthesia and Analgesia. 2018;126(4):572.

40. Casey JD, Vaughan EM, Lloyd BD, Billas PA, Jackson KE, Hall EJ, et al. Protocolized postextubation respiratory support to prevent reintubation a randomized clinical trial. American Journal of Respiratory and Critical Care Medicine. 2021;204(3):294-302. doi: 10.1164/rccm.202009-3561OC.

41. Cesar R, Bispo B, Felix PH, Modolo MC, Cabó S, Souza A, et al. A randomized controlled trial of high-flow nasal cannula versus cpap in critical bronchiolitis. Critical Care Medicine. 2018;46:553. doi: 10.1097/01.ccm.0000529144.79628.12.

42. Cesar RG, Bispo BRP, Felix PHCA, Modolo MCC, Souza AAF, Horigoshi NK, et al. High-Flow Nasal Cannula versus Continuous Positive Airway Pressure in Critical Bronchiolitis: A Randomized Controlled Pilot. Journal of Pediatric Intensive Care. 2020;9(4):248-55. doi: 10.1055/s-0040-1709656.

43. Cetinkaya M, Cebeci B, Semerci SY, Kurnaz D, Saǧlam O. Comparison of three different non-invasive ventilation modes in preterm infants with respiratory distress syndrome: Prospective randomized study. Journal of Neonatal-Perinatal Medicine. 2018;11(2):227-8. doi: 10.3233/NPM189001.

44. Chen J, Lin Y, Du L, Kang M, Chi X, Wang Z, et al. The Comparison of HHHFNC and NCPAP in Extremely Low-Birth-Weight Preterm Infants After Extubation: A Single-Center Randomized Controlled Trial. Frontiers in Pediatrics. 2020;8. doi: 10.3389/fped.2020.00250.

45. Chisti MJ, Salam MA, Smith JH, Ahmed T, Pietroni MAC, Shahunja KM, et al. Bubble continuous positive airway pressure for children with severe pneumonia and hypoxaemia in Bangladesh: An open, randomised controlled trial. The Lancet. 2015;386(9998):1057-65. doi: 10.1016/S0140-6736(15)60249-5.

46. Cho JY, Kim HS, Kang H, Kim SH, Choe KH, Lee KM, et al. Comparison of Postextubation Outcomes Associated with High-Flow Nasal Cannula vs. Conventional Oxygen Therapy in Patients at High Risk of Reintubation: a Randomized Clinical Trial. Journal of Korean medical science. 2020;35(25):e194. doi: 10.3346/jkms.2020.35.e194.

47. Christophe M, Florence PA, Anne D, Robin P, Jean-Michel L, Camille G, et al. Can a flow rate of 3 L/kg/min, compared to 2 L/kg/min, reduce the risk of failure during the initial management of acute viral bronchiolitis with high flow nasal cannulae: A randomized controlled trial (TRAMONTANE 2 study). Annals of Intensive Care. 2018;8(1). doi: 10.1186/s13613-017-0345-7.

48. Chua MT, Khan FA, Ng WM, Lu Q, Low MJW, Yau YW, et al. Pre- and Apnoeic high flow oxygenation for RApid sequence intubation in the Emergency department (Pre-AeRATE): Study protocol for a multicentre, randomised controlled trial. Trials. 2019;20(1). doi: 10.1186/s13063-019-3305-8.

49. Chua MT, Ng WM, Lu Q, Low MJW, Punyadasa A, Cove ME, et al. Pre- and apnoeic high-flow oxygenation for rapid sequence intubation in the emergency department (the Pre-AeRATE trial): A multicentre randomised controlled trial. Annals of the Academy of Medicine, Singapore. 2022;51(3):149-60. doi: 10.47102/annals-acadmedsg.2021407.

50. Cırıl MF, Akarca M, Unal Akoglu E, Cimilli Ozturk T, Onur Ö. High-Flow Nasal Cannula versus Bag Valve Mask for Preoxygenation during Rapid Sequence Intubation in the Emergency Department: A Single-Center, Prospective, Randomized Controlled Trial. Prehospital and disaster medicine. 2024;39(1):45-51. doi: 10.1017/S1049023X23006684.

51. Connolly B, Perkins GD, Ji C, Couper K, Lall R, Baillie JK, et al. RCT Abstract- An adaptive randomized controlled trial of non-invasive respiratory strategies in acute respiratory failure patients with COVID-19. European Respiratory Journal. 2021;58(SUPPL 65). doi: 10.1183/13993003.congress-2021.RCT4271.

52. Corley A, Bull T, Spooner AJ, Barnett AG, Fraser JF. Direct extubation onto high-flow nasal cannulae post-cardiac surgery versus standard treatment in patients with a BMI ≥30: a randomised controlled trial. Intensive Care Medicine. 2015. doi: 10.1007/s00134-015-3765-6.

53. Corral-Gudino L, Cusacovich I, Martín-González JI, Muela-Molinero A, Abadía-Otero J, González-Fuentes R, et al. Effect of intravenous pulses of methylprednisolone 250 mg versus dexamethasone 6 mg in hospitalised adults with severe COVID-19 pneumonia: An open-label randomised trial. European Journal of Clinical Investigation. 2023;53(1). doi: 10.1111/eci.13881. PubMed Central PMCID: PMCAstra Zeneca

Jansen

Pfizer.

54. Cortegiani A, Longhini F, Carlucci A, Scala R, Groff P, Bruni A, et al. High-flow nasal therapy versus noninvasive ventilation in COPD patients with mild-to-moderate hypercapnic acute respiratory failure: Study protocol for a noninferiority randomized clinical trial. Trials. 2019;20(1). doi: 10.1186/s13063-019-3514-1.

55. Coudroy R, Frat JP, Ehrmann S, Pène F, Decavèle M, Terzi N, et al. High-flow nasal oxygen alone or alternating with non-invasive ventilation in critically ill immunocompromised patients with acute respiratory failure: a randomised controlled trial. The Lancet Respiratory Medicine. 2022;10(7):641-9. doi: 10.1016/S2213-2600(22)00096-0.

56. Coudroy R, Frat JP, Ehrmann S, Pène F, Terzi N, Decavèle M, et al. High-flow nasal oxygen therapy alone or with non-invasive ventilation in immunocompromised patients admitted to ICU for acute hypoxemic respiratory failure: The randomised multicentre controlled FLORALI-IM protocol. BMJ Open. 2019;9(8). doi: 10.1136/bmjopen-2019-029798.

57. Daish H, Badurdeen S. Heated humidified high-flow nasal cannula versus nasal continuous positive airway pressure for postextubation ventilatory support in neonates: A meta-analysis. Journal of Maternal-Fetal and Neonatal Medicine. 2014;27:208. doi: 10.3109/14767058.2014.924236.

58. De Jong A, Bignon A, Stephan F, Godet T, Constantin JM, Asehnoune K, et al. Effect of non-invasive ventilation after extubation in critically ill patients with obesity in France: a multicentre, unblinded, pragmatic randomised clinical trial. The Lancet Respiratory Medicine. 2023;11(6):530-9. doi: 10.1016/S2213-2600(22)00529-X.

59. De Jong A, Huguet H, Molinari N, Jaber S. Non-invasive ventilation versus oxygen therapy after extubation in patients with obesity in intensive care units: The multicentre randomised EXTUB-OBESE study protocol. BMJ Open. 2022;12(1). doi: 10.1136/bmjopen-2021-052712.

60. De Jong A, Monnin M, Girard M, Chanques G, Molinari N, Jaber S. Apnoeic oxygenation via high-flow nasal oxygen combined with non-invasive ventilation preoxygenation for intubation in hypoxaemic patients in intensive care unit: The randomised OPTINIV study. Annals of Intensive Care. 2017;7(1):23-4. doi: 10.1186/s13613-016-0223-8.

61. Demirel G, Vatansever B, Tastekin A. High flow nasal cannula versus nasal continuous positive airway pressure for primary respiratory support in preterm infants: A prospective randomized study. American Journal of Perinatology. 2021;38(3):237-41. doi: 10.1055/s-0039-1696673.

62. Demoule A, Baptiste A, Thille A, Similowski T, Ragot S, Prat G, et al. Dyspnea Is Associated With a Higher Intubation Rate and Mortality in De Novo Acute Hypoxemic Respiratory Failure - A Secondary Analysis of a Randomized Trial. American Journal of Respiratory and Critical Care Medicine. 2023;207(1). doi: 10.1164/ajrccm-conference.2023.A46.

63. Devraj P, Shrotey VR, Yogesh, Zanwar. Effect on oxygenation with high flow nasal cannula (HFNC) vs conventional oxygen therapy (Venturi mask) after extubation in patients undergoing cardiac surgery. Indian Journal of Anaesthesia. 2020;64(13):S4. doi: 10.4103/0019-5049.277899.

64. Ding Y, Huang T, Ge Y, Gao J, Zhang Y. Effect of trans-nasal humidified rapid insufflation ventilatory exchange on reflux and microaspiration in patients undergoing laparoscopic cholecystectomy during induction of general anesthesia: a randomized controlled trial. Frontiers in Medicine. 2023;10. doi: 10.3389/fmed.2023.1212646.

65. Dodge D, Whittle JS, Bublewicz MB, Kearney J, Ashe T, Harsch M, et al. High velocity nasal insufflation in hypercapneic respiratory failure: Secondary analysis of randomized clinical trial. American Journal of Respiratory and Critical Care Medicine. 2018;197(MeetingAbstracts).

66. Doshi P, Whittle J, Bubblewicz M, Kearny J, Ashe T, Graham R, et al. High velocity nasal insufflation compared to non-invasive positive pressure ventilation in the treatment of respiratory failure in the emergency department: A randomized clinical trial. Academic Emergency Medicine. 2017;24:S19. doi: 10.1111/acem.13203.

67. Doshi PB, Whittle JS, Dungan G, Volakis LI, Bublewicz M, Kearney J, et al. The ventilatory effect of high velocity nasal insufflation compared to non-invasive positive-pressure ventilation in the treatment of hypercapneic respiratory failure: A subgroup analysis. Heart and Lung. 2020;49(5):610-5. doi: 10.1016/j.hrtlng.2020.03.008.

68. Doyle AJ, Stolady D, Mariyaselvam M, Wijewardena G, Gent E, Blunt M, et al. Preoxygenation and apneic oxygenation using Transnasal Humidified Rapid-Insufflation Ventilatory Exchange for emergency intubation. Journal of Critical Care. 2016;36:8-12. doi: 10.1016/j.jcrc.2016.06.011.

69. Duan X, Wei N, Wei J, Zhu Y, Kang Y, He Y, et al. Effect of High-Flow Nasal Cannula Oxygen Therapy on Pediatric Patients With Congenital Heart Disease in Procedural Sedation: A Prospective, Randomized Trial. Journal of Cardiothoracic and Vascular Anesthesia. 2021;35(10):2913-9. doi: 10.1053/j.jvca.2021.03.031.

70. Dumas G, Chevret S, Lemiale V, Mokart D, Mayaux J, Pène F, et al. Initial ventilation strategy and risk for intubation in immunocompromised patients with acute respiratory failure. Annals of Intensive Care. 2017;7(1):147-8. doi: 10.1186/s13613-016-0224-7.

71. Ehrmann S, Li J, Ibarra-Estrada M, Perez Y, Pavlov I, McNicholas B, et al. Awake prone positioning for COVID-19 acute hypoxaemic respiratory failure: a randomised, controlled, multinational, open-label meta-trial. The Lancet Respiratory Medicine. 2021;9(12):1387-95. doi: 10.1016/S2213-2600(21)00356-8.

72. Elagamy AE, Taha SS, Elfawy DM. High flow nasal cannula versus non- invasive ventilation in prevention of intubation in immunocompromised patient with acute hypoxemic respiratory failure. Egyptian Journal of Anaesthesia. 2021;37(1):432-9. doi: 10.1080/11101849.2021.1978744.

73. Enayati F, Amini S, Gerdrodbari MG, Jarahi L, Ansari M. Effect of high-flow nasal Oxygen on respiratory parameters and pulmonary complications after early extubation following pediatric heart surgery. Journal of Comprehensive Pediatrics. 2021;12(3). doi: 10.5812/compreped.116104.

74. Ergul AB, Calıskan E, Samsa H, Gokcek I, Kaya A, Zararsiz GE, et al. Using a high-flow nasal cannula provides superior results to OxyMask delivery in moderate to severe bronchiolitis: a randomized controlled study. European Journal of Pediatrics. 2018;177(8):1299-307. doi: 10.1007/s00431-018-3191-1.

75. Essghaier S. Preoxygenation in SARS-COV-2 hypoxemic pneumonia. Intensive Care Medicine Experimental. 2022;10. doi: 10.1186/s40635-022-00468-1.

76. Fakharian A, Raeisi S. Value and safety of high flow oxygenation in the treatment of inpatient asthma, a randomized, double-blind, clinical trial. American Journal of Respiratory and Critical Care Medicine. 2019;199(9).

77. Farhat AS, Mohammadzadeh A, Mamuri GA, Saeidi R, Noorizadeh S. Comparison of nasal non-invasive ventilation methods in preterm neonates with respiratory distress syndrome. Iranian Journal of Neonatology. 2018;9(4):53-60. doi: 10.22038/ijn.2018.24544.1313.

78. Fong KM, Au SY, Ng GWY. Preoxygenation before intubation in adult patients with acute hypoxemic respiratory failure: A network meta-analysis of randomized trials. Critical Care. 2019;23(1). doi: 10.1186/s13054-019-2596-1.

79. Foran J, Moore CM, Ni Chathasaigh CM, Moore S, Purna JR, Curley A. Nasal high-flow therapy to Optimise Stability during Intubation: The NOSI pilot trial. Archives of Disease in Childhood: Fetal and Neonatal Edition. 2023;108(3):244-9. doi: 10.1136/archdischild-2022-324649.

80. Forsberg IM, Mkrtchian S, Ebberyd A, Ullman J, Eriksson LI, Lodenius Å, et al. Biomarkers for oxidative stress and organ injury during Transnasal Humidified Rapid-Insufflation Ventilatory Exchange compared to mechanical ventilation in adults undergoing microlaryngoscopy: A randomised controlled study. Acta Anaesthesiologica Scandinavica. 2021;65(9):1276-84. doi: 10.1111/aas.13927. PubMed Central PMCID: PMCB Braun(Germany)

Merck Sharp and Dohme(Netherlands)

Glaxo SmithKline(Sweden).

81. Franklin D, Dalziel S, Schlapbach LJ, Babl FE, Oakley E, Craig SS, et al. Early high flow nasal cannula therapy in bronchiolitis, a prospective randomised control trial (protocol): A Paediatric Acute Respiratory Intervention Study (PARIS). BMC Pediatrics. 2015;15(1). doi: 10.1186/s12887-015-0501-x.

82. Frat JP, Coudroy R, Ragot S, Constantin JM, Prat G, Mercat A, et al. Acute hypoxemic respiratory failure: Which patients need intubation? Annals of Intensive Care. 2017;7(1):148. doi: 10.1186/s13613-016-0224-7.

83. Frat JP, Quenot JP, Badie J, Coudroy R, Guitton C, Ehrmann S, et al. Effect of High-Flow Nasal Cannula Oxygen vs Standard Oxygen Therapy on Mortality in Patients with Respiratory Failure Due to COVID-19: The SOHO-COVID Randomized Clinical Trial. JAMA. 2022;328(12):1212-22. doi: 10.1001/jama.2022.15613.

84. Frat JP, Ragot S, Coudroy R, Constantin JM, Girault C, Prat G, et al. Predictors of intubation in patients with acute hypoxemic respiratory failure treated with a noninvasive oxygenation strategy. Critical Care Medicine. 2018;46(2):208-15. doi: 10.1097/CCM.0000000000002818.

85. Frat JP, Ragot S, Coudroy R, Constantin JM, Girault C, Prat G, et al. Acute hypoxemic respiratory failure: Which patients need intubation? Intensive Care Medicine Experimental. 2017;5(2). doi: 10.1186/s40635-017-0151-4.

86. Frat JP, Ricard JD, Coudroy R, Robert R, Ragot S, Thille AW. Preoxygenation with non-invasive ventilation versus high-flow nasal cannula oxygen therapy for intubation of patients with acute hypoxaemic respiratory failure in ICU: The prospective randomised controlled FLORALI-2 study protocol. BMJ Open. 2017;7(12). doi: 10.1136/bmjopen-2017-018611.

87. Frat JP, Ricard JD, Quenot JP, Pichon N, Demoule A, Forel JM, et al. Non-invasive ventilation versus high-flow nasal cannula oxygen therapy with apnoeic oxygenation for preoxygenation before intubation of patients with acute hypoxaemic respiratory failure: a randomised, multicentre, open-label trial. The Lancet Respiratory Medicine. 2019;7(4):303-12. doi: 10.1016/S2213-2600(19)30048-7.

88. Freundlich RE. Reducing Reintubation Risk in High-Risk Cardiac Surgery Patients with High Flow Nasal Cannula. Journal of Clinical and Translational Science. 2019;3:29. doi: 10.1017/cts.2019.70.

89. Futier E, Paugam-Burtz C, Constantin JM, Pereira B, Jaber S. The OPERA trial - comparison of early nasal high flow oxygen therapy with standard care for prevention of postoperative hypoxemia after abdominal surgery: Study protocol for a multicenter randomized controlled trial. Trials. 2013;14(1). doi: 10.1186/1745-6215-14-341.

90. George S, Gibbons K, Williams T, Humphreys S, Gelbart B, Le Marsney R, et al. Transnasal Humidified Rapid Insufflation Ventilatory Exchange in children requiring emergent intubation (Kids THRIVE): a statistical analysis plan for a randomised controlled trial. Trials. 2023;24(1). doi: 10.1186/s13063-023-07330-z.

91. George S, Humphreys S, Williams T, Gelbart B, Chavan A, Rasmussen K, et al. Transnasal Humidified Rapid Insufflation Ventilatory Exchange in children requiring emergent intubation (Kids THRIVE): A protocol for a randomised controlled trial. BMJ Open. 2019;9(2). doi: 10.1136/bmjopen-2018-025997.

92. Gibu CK, Cheng PY, Ward RJ, Castro B, Heldt GP. Feasibility and physiological effects of noninvasive neurally adjusted ventilatory assist in preterm infants. Pediatric Research. 2017;82(4):650-7. doi: 10.1038/pr.2017.100.

93. Goodchild K, Spencer SA, Brain L, Loh B, Laver S, Swinburne H, et al. High Flow Nasal Cannula Oxygen in COVID-19; still an important role to play. Journal of the Intensive Care Society. 2022;23(1):47-8. doi: 10.1177/17511437221095122.

94. Grieco DL, Menga LS, Cesarano M, Rosà T, Spadaro S, Bitondo MM, et al. Effect of Helmet Noninvasive Ventilation vs High-Flow Nasal Oxygen on Days Free of Respiratory Support in Patients with COVID-19 and Moderate to Severe Hypoxemic Respiratory Failure: The HENIVOT Randomized Clinical Trial. JAMA - Journal of the American Medical Association. 2021;325(17):1731-43. doi: 10.1001/jama.2021.4682.

95. Grover R, Singh P, Shubham S, Priyadarshi M, Chaurasia S, Basu S. Delivery Room Respiratory Stabilization of Preterm Neonates: A Randomized, Controlled Trial. Indian Journal of Pediatrics. 2022;89(8):793-800. doi: 10.1007/s12098-022-04124-0.

96. Guia M, Alpay N, Gerardo A, Madney Y, Abdelrahim M, Saeed H, et al. High-flow nasal oxygen therapy in acute hypoxemic respiratory failure: Concise review on technology and initial methodology. Turkish Thoracic Journal. 2021;22(6):494-500. doi: 10.5152/TurkThoracJ.2021.20213.

97. Guitton C, Ehrmann S, Volteau C, Colin G, Maamar A, Jean-Michel V, et al. Nasal high-flow preoxygenation for endotracheal intubation in the critically ill patient: a randomized clinical trial. Intensive Care Medicine. 2019;45(4):447-58. doi: 10.1007/s00134-019-05529-w.

98. Guoqiang F, Qiufeng W, Yajie T, Wenting J, Xi L, Ting Y, et al. Comparative study on pros and cons of sequential high-flow nasal cannula and non-invasive positive pressure ventilation immediately following early extubated patients with severe respiratory failure due to acute exacerbations of chronic obstructive pulmonary disease. Chinese Critical Care Medicine. 2021;33(10):1215-20. doi: 10.3760/cma.j.cn121430-20210623-00939.

99. Gupta S, Govil D, Srinivasan S, Patel SJ, Gupta A, Tomar DS, et al. High flow nasal cannula (HFNC) as an alternative to noninvasive ventilation (NIV) in acute respiratory failure (ARF) in immunosuppressed patients-an Indian post liver transplant experience. Intensive Care Medicine Experimental. 2016;4. doi: 10.1186/s40635-016-0099-9.

100. Hameed IZS, Hazra D, Ganesan P, Prabhakar AKP. Does apneic oxygenation with nasopharyngeal cannula during intubation improve the oxygenation in patients with acute hypoxemic respiratory failure compared to the standard bag valve mask preoxygenation? An open‑labeled randomized control trial. Turkish Journal of Emergency Medicine. 2024;24(1):33-40. doi: 10.4103/tjem.tjem_176_23.

101. Hamp T, Prager G, Baron-Stefaniak J, Müller J, Bichler C, Plöchl W. Duration of safe apnea in patients with morbid obesity during passive oxygenation using high-flow nasal insufflation versus regular flow nasal insufflation, a randomized trial. Surgery for Obesity and Related Diseases. 2021;17(2):347-55. doi: 10.1016/j.soard.2020.09.027.

102. Hanouz JL, Lhermitte D, Gerard JL, Fischer MO. Comparison of pre-oxygenation using spontaneous breathing through face mask and high-flow nasal oxygen A randomised controlled crossover study in healthy volunteers. European Journal of Anaesthesiology. 2019;36(5):335-41. doi: 10.1097/EJA.0000000000000954.

103. Hao J, Liu J, Pu L, Li C, Zhang M, Tan J, et al. High-Flow Nasal Cannula Oxygen Therapy versus Non-Invasive Ventilation in AIDS Patients with Acute Respiratory Failure: A Randomized Controlled Trial. Journal of Clinical Medicine. 2023;12(4). doi: 10.3390/jcm12041679.

104. Hathorn C, Ernst G, Hasan S, Wong D, Seear M. The hi-FLO study: A prospective open randomised controlled trial of high flow nasal cannula oxygen therapy against standard care in bronchiolitis. Thorax. 2014;69:A38. doi: 10.1136/thoraxjnl-2014-206260.74.

105. Haywood ST, Whittle JS, Bublewicz M, Kearney J, Ashe T, Miller T, et al. High velocity nasal insufflation vs noninvasive positive pressure ventilation in heart failure. Academic Emergency Medicine. 2018;25:S181. doi: 10.1111/acem.13424.

106. Haywood ST, Whittle JS, Volakis LI, Dungan G, Bublewicz M, Kearney J, et al. HVNI vs NIPPV in the treatment of acute decompensated heart failure: Subgroup analysis of a multi-center trial in the ED. American Journal of Emergency Medicine. 2019;37(11):2084-90. doi: 10.1016/j.ajem.2019.03.002.

107. He R, Fang Y, Jiang Y, Yao D, Li Z, Zheng W, et al. High-flow nasal oxygenation versus face mask oxygenation for preoxygenation in patients undergoing double-lumen endobronchial intubation: Protocol of a randomised controlled trial. BMJ Open. 2024;14(3). doi: 10.1136/bmjopen-2023-080422.

108. Hernández G, Paredes I, Moran F, Buj M, Colinas L, Rodríguez ML, et al. Effect of postextubation noninvasive ventilation with active humidification vs high-flow nasal cannula on reintubation in patients at very high risk for extubation failure: a randomized trial. Intensive Care Medicine. 2022;48(12):1751-9. doi: 10.1007/s00134-022-06919-3.

109. Hernández G, Vaquero C, González P, Subira C, Frutos-Vivar F, Rialp G, et al. Effect of postextubation high-flow nasal cannula vs conventional oxygen therapy on reintubation in low-risk patients: A randomized clinical trial. JAMA - Journal of the American Medical Association. 2016;315(13):1354-61. doi: 10.1001/jama.2016.2711.

110. Hinckley D, Jones J. Outcomes of Children With Bronchiolitis Treated With High-Flow Nasal Cannula or Noninvasive Positive Pressure Ventilation: Clayton JA, McKee B, Slain K, et al. Pediatr Crit Care Med. 2019;20(2):128-135. Journal of Emergency Medicine. 2019;57(3):417-8. doi: 10.1016/j.jemermed.2019.08.019.

111. Hinojosa Camargo WE, Iglesias Echeverria AC, Vera Vera SH, Cabezon Villalba G, Aparisi Sanz A, Gomez I, et al. Prognostic impact of high flow nasal cannula compared to noninvasive positive-pressure ventilation in the treatment of acute pulmonary edema. European Heart Journal: Acute Cardiovascular Care. 2021;10(SUPPL 1):i38. doi: 10.1093/ehjacc/zuab020.033.

112. Hodgson KA, Owen LS, Kamlin CO, Roberts CT, Donath SM, Davis PG, et al. A multicentre, randomised trial of stabilisation with nasal high flow during neonatal endotracheal intubation (the SHINE trial): A study protocol. BMJ Open. 2020;10(10). doi: 10.1136/bmjopen-2020-039230.

113. Hodgson KA, Owen LS, Kamlin CO, Roberts CT, Newman SE, Francis K, et al. NASAL HIGH-FLOW DURING NEONATAL ENDOTRACHEAL INTUBATION. Journal of Paediatrics and Child Health. 2022;58(SUPPL 2):11-2. doi: 10.1111/jpc.15945.

114. Hodgson KA, Owen LS, Kamlin COF, Roberts CT, Newman SE, Francis KL, et al. Nasal High-Flow Therapy during Neonatal Endotracheal Intubation. New England Journal of Medicine. 2022;386(17):1627-37. doi: 10.1056/NEJMoa2116735.

115. Homer E, Gilbert R, Hardelid P. Effect of high flow nasal oxygen in bronchiolitis: Before-and-after purchase study. Archives of Disease in Childhood. 2019;104:A45. doi: 10.1136/archdischild-2019-rcpch.106.

116. Hu J, Zhang J. High-flow nasal oxygen versus conventional oxygen therapy during cesarean section under neuraxial anesthesia in pregnant women with heart disease: a randomized controlled trial. Nan fang yi ke da xue xue bao = Journal of Southern Medical University. 2024;44(6):1040-7. doi: 10.12122/j.issn.1673-4254.2024.06.04.

117. Hua Z, Liu Z, Li Y, Zhang H, Yang M, Zuo M. Transnasal humidified rapid insufflation ventilatory exchange vs. facemask oxygenation in elderly patients undergoing general anaesthesia: a randomized controlled trial. Scientific reports. 2020;10(1):5745. doi: 10.1038/s41598-020-62716-2.

118. Ibarra-Estrada M, Li J, Pavlov I, Perez Y, Roca O, Tavernier E, et al. Factors for success of awake prone positioning in patients with COVID-19-induced acute hypoxemic respiratory failure: analysis of a randomized controlled trial. Critical Care. 2022;26(1). doi: 10.1186/s13054-022-03950-0.

119. Ibarra-Estrada M, Vargas-Obieta A, Marin-Rosales M, Aguirre-Díaz S, García-Salcido R, López-Pulgarín J, et al. Prone positioning in awake patients with COVID-19-associated respiratory failure: The procarf trial. Critical Care Medicine. 2022;50(1 SUPPL):10. doi: 10.1097/01.ccm.0000806544.31055.86.

120. Ibarra-Estrada MÁ, Marín-Rosales M, García-Salcido R, Aguirre-Díaz SA, Vargas-Obieta A, Chávez-Peña Q, et al. Prone positioning in non-intubated patients with COVID-19 associated acute respiratory failure, the PRO-CARF trial: A structured summary of a study protocol for a randomised controlled trial. Trials. 2020;21(1). doi: 10.1186/s13063-020-04882-2.

121. Irfan M, Ahmed M, Breen D. Assessment of High Flow Nasal Cannula Oxygenation in Endobronchial Ultrasound Bronchoscopy: A Randomized Controlled Trial. Journal of Bronchology and Interventional Pulmonology. 2021;28(2):130-7. doi: 10.1097/LBR.0000000000000719.

122. Ishii K, Morimatsu H, Hyodo T, Ono K, Hidaka H, Koyama Y, et al. Relationship between inspired oxygen concentration and atelectasis formation after extubation. Critical Care Medicine. 2018;46:533. doi: 10.1097/01.ccm.0000529104.66235.9e.

123. Itdhi-Amornkulchai S, Anantasit N, Preutthipan A. Modified versus conventional heated humidified high flow nasal cannula in children with respiratory distress, a prospective study. Pediatric Critical Care Medicine. 2018;19(6):69.

124. Jaber S, Molinari N, De Jong A. New method of preoxygenation for orotracheal intubation in patients with hypoxaemic acute respiratory failure in the intensive care unit, non-invasive ventilation combined with apnoeic oxygenation by high flow nasal oxygen: the randomised OPTINIV study protocol. BMJ Open. 2016;6(8). doi: 10.1136/BMJOPEN-2016-011298.

125. Jaber S, Monnin M, Girard M, Conseil M, Cisse M, Carr J, et al. Apnoeic oxygenation via high-flow nasal cannula oxygen combined with non-invasive ventilation preoxygenation for intubation in hypoxaemic patients in the intensive care unit: the single-centre, blinded, randomised controlled OPTINIV trial. Intensive Care Medicine. 2016;42(12):1877-87. doi: 10.1007/s00134-016-4588-9.

126. Jayakumar D, Ramachandran DP, Rabindrarajan DE, Vijayaraghavan MBKT, Ramakrishnan AN, Venkataraman AR. Standard Care Versus Awake Prone Position in Adult Nonintubated Patients With Acute Hypoxemic Respiratory Failure Secondary to COVID-19 Infection—A Multicenter Feasibility Randomized Controlled Trial. Journal of Intensive Care Medicine. 2021;36(8):918-24. doi: 10.1177/08850666211014480.

127. Jo JY, Kim WJ, Ku S, Choi SS. Comparison of preoxygenation with a high-flow nasal cannula and a simple mask before intubation during induction of general anesthesia in patients undergoing head and neck surgery: Study protocol clinical trial (SPIRIT Compliant). Medicine (United States). 2020;99(12). doi: 10.1097/MD.0000000000019525.

128. Jovaisa T, Januskeviciute E, Grinkeviciute G, Montvilaite I, Krauklyte J, Kalimavičius A, et al. Hypoxaemic respiratory failure and awake prone ventilation (HYPER-AP) - Protocol for randomized, controlled clinical trial. Contemporary clinical trials. 2024:107614. doi: 10.1016/j.cct.2024.107614.

129. Kadivar M, Mosayebi Z, Razi N, Nariman S, Sangsari R. High flow nasal cannulae versus nasal continuous positive airway pressure in neonates with respiratory distress syndrome managed with INSURE method: A randomized clinical trial. Iranian Journal of Medical Sciences. 2016;41(6):494-500.

130. Kamga H, Frugier A, Boutros M, Bourges J, Doublet T, Parienti JJ. Flexible nasal bronchoscopy vs. Airtraq® videolaryngoscopy for awake tracheal intubation: a randomised controlled non-inferiority study. Anaesthesia. 2023;78(8):963-9. doi: 10.1111/anae.16042.

131. Kang BJ, Koh Y, Lim CM, Huh JW, Baek S, Han M, et al. Failure of high-flow nasal cannula therapy may delay intubation and increase mortality. Intensive Care Medicine. 2015. doi: 10.1007/s00134-015-3693-5.

132. Kang H, Zhao Z, Tong Z. Effect of high-flow nasal cannula oxygen therapy in immunocompromised subjects with acute respiratory failure. Respiratory Care. 2020;65(3):369-76. doi: 10.4187/respcare.07205.

133. Kang WQ, Xu BL, Liu DP, Zhang YD, Guo J, Li ZH, et al. Efficacy of heated humidified high-flow nasal cannula in preterm infants aged less than 32 weeks after ventilator weaning. Chinese Journal of Contemporary Pediatrics. 2016;18(6):488-91. doi: 10.7499/j.issn.1008-8830.2016.06.004.

134. Karcioǧlu AM, Atli HZ, Karcioǧlu O, Turan IÖ. The Evaluation of COVID-19 Patients Treated with HFNC in ICU. Turkish Journal of Intensive Care. 2022;20(1):104.

135. Kaur R, Vines DL, Mirza S, Elshafei A, Jackson JA, Harnois LJ, et al. Early versus late awake prone positioning in non-intubated patients with COVID-19. Critical Care. 2021;25(1). doi: 10.1186/s13054-021-03761-9.

136. Khalili S, Amiri-Farahani L, Haghani S, Bordbar A, Shojaii A, Pezaro S. The effect of Pimpinella Anisum herbal tea on human milk volume and weight gain in the preterm infant: a randomized controlled clinical trial. BMC Complementary Medicine and Therapies. 2023;23(1). doi: 10.1186/s12906-023-03848-6.

137. Khatib MY, Peediyakkal MZ, Elshafei MS, Elzeer HS, Ananthegowda DC, Shahen MA, et al. Comparison of the clinical outcomes of noninvasive ventilation by helmet vs facemask in patients with acute respiratory distress syndrome. Medicine (United States). 2021;100(4). doi: 10.1097/MD.0000000000024443.

138. Kienzle M, Attebery J, Custer J, Remy K. High-flow nasal oxygen reduces intubation in critically ill children with respiratory failure. Critical Care Medicine. 2016;44(12):331. doi: 10.1097/01.ccm.0000509697.97426.5b.

139. Ko DR, Beom J, Lee HS, You JS, Chung HS, Chung SP. Benefits of high-flow nasal cannula therapy for acute pulmonary edema in patients with heart failure in the emergency department: A prospective multi-center randomized controlled trial. Journal of Clinical Medicine. 2020;9(6):1-14. doi: 10.3390/jcm9061937.

140. Kopp W, Gedeit R, McLaughlin G, Asaro L, Wypij D, Curley M. Negative effects of pre-intubation NIV use in pediatric ARDS. Critical Care Medicine. 2018;46:11. doi: 10.1097/01.ccm.0000528077.93125.e9.

141. Koretz RL. JPEN Journal Club 73. When it is too good to be true. Journal of Parenteral and Enteral Nutrition. 2023;47(4):580-2. doi: 10.1002/jpen.2454.

142. Kugelman A. Controversies and update on non invasive ventilation in the nicu. Pediatric Pulmonology. 2017;52:S73-S4. doi: 10.1002/ppul.23729.

143. Kugelman A. Utility of high-flow nasal cannula therapy in the NICU. Pediatric Pulmonology. 2018;53:S31-S2. doi: 10.1002/ppul.24031.

144. Kugelman A. Neonatal pulmonology: 'year in review' for the pediatric pulmonologist. Pediatric Pulmonology. 2021;56(SUPPL 2):S45-S7. doi: 10.1002/ppul.25498.

145. Kugelman A. Neonatal Pulmonology: “Year in Review” for the Pediatric Pulmonologist. Pediatric Pulmonology. 2022;57:S8-S10. doi: 10.1002/ppul.25960.

146. Kumar V, Angurana SK, Baranwal AK, Nallasamy K. Nasotracheal vs. Orotracheal Intubation and Post-extubation Airway Obstruction in Critically Ill Children: An Open-Label Randomized Controlled Trial. Frontiers in Pediatrics. 2021;9. doi: 10.3389/fped.2021.713516.

147. Kumari N, Kumari B, Kumar S, Arun N, Kumari R. Effectiveness of high flow nasal cannula (HFNC) versus bilevel positive airway pressure (BiPAP) in preventing tracheal reintubation in patients with high risk of extubation failure in intensive care unit-A randomised comparative trial. Indian Journal of Anaesthesia. 2024;68(3):246-53. doi: 10.4103/ija.ija_620_23.

148. Kwizera A, Kabatoro D, Owachi D, Kansiime J, Kateregga G, Nanyunja D, et al. Respiratory support with standard low-flow oxygen therapy, high-flow oxygen therapy or continuous positive airway pressure in adults with acute hypoxaemic respiratory failure in a resource-limited setting: Protocol for a randomised, open-label, clinical trial - The Acute Respiratory Intervention StudiEs in Africa (ARISE-AFRICA) study. BMJ Open. 2024;14(6). doi: 10.1136/bmjopen-2023-082223.

149. Landau J, Sherman C, Shah S, Naeem I, Ahmad M, Saeed R, et al. DRONEDARONE: A RARE CASE OF INTERSTITIAL LUNG INJURY. Chest. 2023;164(4):A3414-A5. doi: 10.1016/j.chest.2023.07.2219.

150. Lane JE, Ford T, Noelck M, Byrd C. High flow, low results: The limits of high flow nasal cannula in the treatment of bronchiolitis. Paediatric Respiratory Reviews. 2024. doi: 10.1016/j.prrv.2024.06.003.

151. Lanzoni G, Linetsky E, Correa D, Messinger Cayetano S, Alvarez RA, Kouroupis D, et al. Umbilical cord mesenchymal stem cells for COVID-19 acute respiratory distress syndrome: A double-blind, phase 1/2a, randomized controlled trial. Stem Cells Translational Medicine. 2021;10(5):660-73. doi: 10.1002/sctm.20-0472.

152. Lee JH, Ji SH, Jang YE, Kim EH, Kim JT, Kim HS. Application of a High-Flow Nasal Cannula for Prevention of Postextubation Atelectasis in Children Undergoing Surgery: A Randomized Controlled Trial. Anesthesia and Analgesia. 2021;133(2):474-82. doi: 10.1213/ANE.0000000000005285.

153. Lee MK, Choi J, Park B, Kim B, Lee SJ, Kim SH, et al. High flow nasal cannulae oxygen therapy in acute-moderate hypercapnic respiratory failure. Clinical Respiratory Journal. 2018;12(6):2046-56. doi: 10.1111/crj.12772.

154. Lee MK, Kim SH, Lee WY, Yong SJ, Lee SJ, Jung YR. The efficacy of high-flow nasal cannulae oxygen therapy in severe acute exacerbation of chronic obstructive pulmonary disease: A randomized controlled trial. European Respiratory Journal. 2016;48. doi: 10.1183/13993003.congress-2016.PA3058.

155. Lei G, Wu L, Xi C, Xiao Y, Wang G. Transnasal Humidified Rapid Insufflation Ventilatory Exchange Augments Oxygenation in Children With Juvenile Onset Recurrent Respiratory Papillomatosis During Surgery: A Prospective Randomized Crossover Controlled Trial. Anesthesia and Analgesia. 2023;137(3):578-86. doi: 10.1213/ANE.0000000000006521.

156. Lei G, Wu L, Xi C, Xiao Y, Wang G. Transnasal Humidified Rapid Insufflation Ventilatory Exchange Augments Oxygenation in Children With Juvenile Onset Recurrent Respiratory Papillomatosis During Surgery: A Prospective Randomized Crossover Controlled Trial. Anesthesia and analgesia. 2023;137(3):578-86. doi: 10.1213/ANE.0000000000006521.

157. Lemiale V, Dumas G, Demoule A, Pène F, Kouatchet A, Bisbal M, et al. Performance of the ROX index to predict intubation in immunocompromised patients receiving high-flow nasal cannula for acute respiratory failure. Annals of Intensive Care. 2021;11(1). doi: 10.1186/s13613-021-00801-z.

158. Lemiale V, Resche-Rigon M, Mokart D, Pène F, Argaud L, Mayaux J, et al. High-Flow Nasal Cannula Oxygenation in Immunocompromised Patients with Acute Hypoxemic Respiratory Failure: A Groupe de Recherche Respiratoire en Réanimation Onco-Hématologique Study. Critical Care Medicine. 2017;45(3):e274-e80. doi: 10.1097/CCM.0000000000002085.

159. Levitt JE, Festic E, Desai M, Hedlin H, Mahaffey KW, Rogers AJ, et al. The ARREST Pneumonia clinical trial: Rationale and design. Annals of the American Thoracic Society. 2021;18(4):698-708. doi: 10.1513/AnnalsATS.202009-1115SD.

160. Li J, Liu B, Zhou QH, Ni HD, Liu MJ, Deng K. Pre-oxygenation with high-flow oxygen through the nasopharyngeal airway compared to facemask on carbon dioxide clearance in emergency adults: a prospective randomized non-blinded clinical trial. European journal of trauma and emergency surgery : official publication of the European Trauma Society. 2024;50(3):1051-61. doi: 10.1007/s00068-023-02418-2.

161. Li XN, Zhou CC, Lin ZQ, Jia B, Li XY, Zhao GF, et al. High-flow nasal cannula oxygen therapy during anesthesia recovery for older orthopedic surgery patients: A prospective randomized controlled trial. World Journal of Clinical Cases. 2022;10(24):8615-24. doi: 10.12998/wjcc.v10.i24.8615.

162. Li Z, Pang M, Yu Y, Peng T, Hu Z, Niu R, et al. Effect of different ventilation modalities on the early prognosis of patients with sleep apnea after acute ischemic stroke–––protocol for a prospective, open-label and randomised controlled trial. BMC Neurology. 2023;23(1). doi: 10.1186/s12883-023-03117-6.

163. Li Z, Pang M, Zhang J, Mao L, Wang X, Sun P. Effect of ventilation modalities on the early prognosis of patients with poststroke sleep apnea. Annals of Clinical and Translational Neurology. 2024;11(2):355-67. doi: 10.1002/acn3.51956.

164. Lin Y, Zhang X, Li L, Wei M, Zhao B, Wang X, et al. High-flow nasal cannula oxygen therapy and hypoxia during gastroscopy with propofol sedation: a randomized multicenter clinical trial. Gastrointestinal Endoscopy. 2019;90(4):591-601. doi: 10.1016/j.gie.2019.06.033.

165. Linnér A, Lode Kolz K, Klemming S, Bergman N, Lilliesköld S, Markhus Pike H, et al. Immediate skin-to-skin contact may have beneficial effects on the cardiorespiratory stabilisation in very preterm infants. Acta Paediatrica, International Journal of Paediatrics. 2022;111(8):1507-14. doi: 10.1111/apa.16371.

166. Liu C, Cheng WY, Li JS, Tang T, Tan PL, Yang L. High-Flow Nasal Cannula vs. Continuous Positive Airway Pressure Therapy for the Treatment of Children <2 Years With Mild to Moderate Respiratory Failure Due to Pneumonia. Frontiers in Pediatrics. 2020;8. doi: 10.3389/fped.2020.590906.

167. Liu L, Yang W, Zhang C, Wang G. Analysis of the application effect and safety of transnasal humidified rapid insufflation ventilatory exchange technique in hysteroscopic diagnostic and therapeutic surgery. National Medical Journal of China. 2024;104(17):1493-8. doi: 10.3760/cma.j.cn112137-20231213-01374.

168. Liu S, Walline JH, Zhu H, Li Y, Wang C, Liu J. High-flow nasal cannula therapy with sequential noninvasive ventilation versus noninvasive ventilation alone as the initial ventilatory strategy in acute COPD exacerbations: study protocol for a randomized controlled trial. Trials. 2022;23(1). doi: 10.1186/s13063-022-06963-w.

169. Liu T, Duan Y, Li Y, Hu Y, Su L, Zhang A. ChatGPT achieves comparable accuracy to specialist physicians in predicting the efficacy of high-flow oxygen therapy. 2023.

170. Liu X, Ren H, He S. EVALUATION OF THE CLINICAL EFFECT OF "CLUSTER" COLLABORATIVE PROGRAM IN ICU PATIENTS WITH TRACHEAL INTUBATION AND EXTUBATION. Acta Medica Mediterranea. 2022;38(3):1831-6. doi: 10.19193/0393-6384_2022_3_280.

171. Lodenius A, Piehl J, Ostlund A, Ullman J, Fagerlund MJ. Pre-oxygenation in rapid sequence induction anesthesia in adults; Transnasal humidified rapid-insufflation ventilatory exchange (THRIVE) vs facemask breathing. Acta Anaesthesiologica Scandinavica. 2017;61(8):1001. doi: 10.1111/(ISSN)1399-6576.

172. Lodenius Å, Piehl J, Östlund A, Ullman J, Jonsson Fagerlund M. Transnasal humidified rapid-insufflation ventilatory exchange (THRIVE) vs. facemask breathing pre-oxygenation for rapid sequence induction in adults: a prospective randomised non-blinded clinical trial. Anaesthesia. 2018;73(5):564-71. doi: 10.1111/anae.14215. PubMed Central PMCID: PMCBraun(Germany)

Sandoz(Denmark)

Glaxo SmithKline(Italy)

Fresenius Kabi(Sweden)

Meda(Sweden)

Pfizer(Sweden).

173. Luca Grieco D, Menga L, Cesarano M, Spadaro S, Maddalena Bitondo M, Berardi C, et al. Phenotypes of Patients with COVID-19 Who Have a Positive Clinical Response to Helmet Noninvasive Ventilation. American Journal of Respiratory and Critical Care Medicine. 2022;205(3):360-5. doi: 10.1164/rccm.202105-1212LE.

174. Lyons C, McElwain J, Coughlan MG, O’Gorman DA, Harte BH, Kinirons B, et al. Pre-oxygenation with facemask oxygen vs high-flow nasal oxygen vs high-flow nasal oxygen plus mouthpiece: a randomised controlled trial. Anaesthesia. 2022;77(1):40-5. doi: 10.1111/anae.15556.

175. Lyons C, McElwain J, Young O, O'Gorman DA, Harte BH, Kinirons B, et al. The effect of high-flow nasal oxygen flow rate on gas exchange in apnoeic patients: a randomised controlled trial. Anaesthesia. 2024;79(6):576-82. doi: 10.1111/anae.16200.

176. Magdy DM, Metwally A. Direct Extubation to High-Flow Nasal Cannula versus Noninvasive Ventilation in Obese Subjects. Respiratory Care. 2023;68(2):234-40. doi: 10.4187/respcare.10222.

177. Magdy DM, Metwally A. Effect of high‑flow nasal cannula versus non‑invasive ventilation in preventing re‑intubation in high‑risk chronic obstructive pulmonary disease patients: A randomised controlled trial. Lung India. 2023;40(4):312-20. doi: 10.4103/lungindia.lungindia_338_22.

178. Maggiore SM, Idone FA, Vaschetto R, Festa R, Cataldo A, Antonicelli F, et al. Nasal high-flow versus venturi mask oxygen therapy after extubation: Effects on oxygenation, comfort, and clinical outcome. American Journal of Respiratory and Critical Care Medicine. 2014;190(3):282-8. doi: 10.1164/rccm.201402-0364OC.

179. Maggiore SM, Jaber S, Grieco DL, Mancebo J, Zakynthinos S, Demoule A, et al. High-Flow Versus VenturiMask Oxygen Therapy to Prevent Reintubation in Hypoxemic Patients after Extubation: A Multicenter Randomized Clinical Trial. American Journal of Respiratory and Critical Care Medicine. 2022;206(12):1452-62. doi: 10.1164/rccm.202201-0065OC.

180. Makdee O, Monsomboon A, Surabenjawong U, Praphruetkit N, Chaisirin W, Chakorn T, et al. High-Flow Nasal Cannula Versus Conventional Oxygen Therapy in Emergency Department Patients With Cardiogenic Pulmonary Edema: A Randomized Controlled Trial. Annals of Emergency Medicine. 2017;70(4):465-72.e2. doi: 10.1016/j.annemergmed.2017.03.028.

181. Makdee O, Nakornchai T. Efficacy of nasal high flow in emergency patients with cardiogenic pulmonary edema: A randomized controlled trial. Academic Emergency Medicine. 2017;24:S266-S7. doi: 10.1111/acem.13203.

182. Maláska J, Stašek J, Duška F, Balík M, Máca J, Hruda J, et al. Effect of dexamethasone in patients with ARDS and COVID-19 – prospective, multi-centre, open-label, parallel-group, randomised controlled trial (REMED trial): A structured summary of a study protocol for a randomised controlled trial. Trials. 2021;22(1). doi: 10.1186/s13063-021-05116-9.

183. Margarson M. Nasal high flow oxygen therapy. What the anaesthetist should know. Journal of Clinical Monitoring and Computing. 2017;31(3):498. doi: 10.1007/s10877-017-9991-4.

184. Marín T, Aldás I, Galdeano M, Mendiluce L, Solis AJ, Alfaya I, et al. Predictors of Non-invasive Respiratory Support Failure in COVID-19 Pneumonia (RUTIROX). Preliminary Results. European Respiratory Journal. 2022;60. doi: 10.1183/13993003.congress-2022.1497.

185. Marjanovic N, Piton M, Lamarre J, Alleyrat C, Couvreur R, Guenezan J, et al. High-flow nasal cannula oxygen versus noninvasive ventilation for the management of acute cardiogenic pulmonary edema: a randomized controlled pilot study. European journal of emergency medicine : official journal of the European Society for Emergency Medicine. 2024;31(4):267-75. doi: 10.1097/MEJ.0000000000001128.

186. Mauri T, Eronia N, Grasselli G, Tagliabue P, Rona R, Suriano G, et al. Mechanisms underlying relief of dyspnea by high flow nasal cannula in acute respiratory failure patients. American Journal of Respiratory and Critical Care Medicine. 2015;191.

187. Mayock DE, Gogcu S, Puia-Dumitrescu M, Shaw DWW, Wright JN, Comstock BA, et al. Association between Term Equivalent Brain Magnetic Resonance Imaging and 2-Year Outcomes in Extremely Preterm Infants: A Report from the Preterm Erythropoietin Neuroprotection Trial Cohort. Journal of Pediatrics. 2021;239:117-25.e6. doi: 10.1016/j.jpeds.2021.08.040.

188. Meena K, Meena RK. Role of High Flow Nasal Oxygenation During Video Laryngoscopy Assisted Intubation in COVID-positive Patients: A Prospective Randomized Control Trial. Indian Journal of Critical Care Medicine. 2022;26:S50. doi: 10.5005/jp-journals-10071-23712A.105.

189. Mendil N, Temel S, Yüksel RC, Gundogan K, Eser B, Kaynar L, et al. The effect of high flow nasal oxygen in hematological malignancy patients with acute hypoxemic respiratory insufficiency: Prospective, single center, randomized controlled trial. Intensive Care Medicine Experimental. 2019;7. doi: 10.1186/s40635-019-0265-y.

190. Merry AF, van Waart H, Allen SJ, Baker PA, Cumin D, Frampton CMA, et al. Ease and comfort of pre-oxygenation with high-flow nasal oxygen cannulae vs. facemask: a randomised controlled trial. Anaesthesia. 2022;77(12):1346-55. doi: 10.1111/anae.15853.

191. Michi T, Mattana C, Menga LS, Bocci MG, Cesarano M, Rosà T, et al. Long-term outcome of COVID-19 patients treated with helmet noninvasive ventilation vs. high-flow nasal oxygen: a randomized trial. Journal of Intensive Care. 2023;11(1). doi: 10.1186/s40560-023-00669-0.

192. Milesi C, Essouri S, Pouyau R, Liet JM, Afanetti M, Baleine J, et al. Interest of high-flow nasal cannula (HFNC) versus nasal continuous positive airway pressure (nCPAP) during the initial management of severe bronchiolitis in infants: A multicenter randomized controlled trial. Annals of Intensive Care. 2016;6. doi: 10.1186/s13613-016-0114-z.

193. Milési C, Essouri S, Pouyau R, Liet JM, Afanetti M, Portefaix A, et al. High flow nasal cannula (HFNC) versus nasal continuous positive airway pressure (nCPAP) for the initial respiratory management of acute viral bronchiolitis in young infants: a multicenter randomized controlled trial (TRAMONTANE study). Intensive Care Medicine. 2017;43(2):209-16. doi: 10.1007/s00134-016-4617-8.

194. Milési C, Pierre AF, Deho A, Pouyau R, Liet JM, Guillot C, et al. A multicenter randomized controlled trial of a 3-L/kg/min versus 2-L/kg/min high-flow nasal cannula flow rate in young infants with severe viral bronchiolitis (TRAMONTANE 2). Intensive Care Medicine. 2018;44(11):1870-8. doi: 10.1007/s00134-018-5343-1.

195. Min SH, Yoon H, Huh G, Kwon SK, Seo JH, Cho YJ. Efficacy of high-flow nasal oxygenation compared with tracheal intubation for oxygenation during laryngeal microsurgery: a randomised non-inferiority study. British Journal of Anaesthesia. 2022;128(1):207-13. doi: 10.1016/j.bja.2021.09.016.

196. Mir F, Patel A, Iqbal R, Cecconi M, Nouraei SAR. A randomised controlled trial comparing transnasal humidified rapid insufflation ventilatory exchange (THRIVE) pre-oxygenation with facemask pre-oxygenation in patients undergoing rapid sequence induction of anaesthesia. Anaesthesia. 2017;72(4):439-43. doi: 10.1111/anae.13799.

197. Mohanty S. High flow oxygen through nasal canula as alternative to continuous positive airway pressure ventilation as initial respiratory support for children with hypoxic respiratory failure. Pediatric Critical Care Medicine. 2021;22(SUPPL 1):1. doi: 10.1097/01.pcc.0000738112.96536.2b.

198. Morsy RS, Sedky Badawy MM, Said RN, Ali AA, Abuelhamd WA. A comparative study between postextubation of preterm neonates into high-flow nasal cannula versus nasal continuous positive airway pressure. Iranian Journal of Neonatology. 2021;12(1):12-9. doi: 10.22038/ijn.2020.43810.1753.

199. Murphy K, Kessel A. High-flow nasal cannula versus bilevel positive pressure ventilation for status asthmaticus. Critical Care Medicine. 2019;47(1).

200. Nagata K, Yokoyama T, Tsugitomi R, Nakashima H, Kuraishi H, Ohshimo S, et al. Continuous positive airway pressure versus high-flow nasal cannula oxygen therapy for acute hypoxemic respiratory failure: A randomized controlled trial. Respirology. 2024;29(1):36-45. doi: 10.1111/resp.14588.

201. Nair PR, Haritha D, Behera S, Kayina CA, Maitra S, Anand RK, et al. Comparison of high-flow nasal cannula and noninvasive ventilation in acute hypoxemic respiratory failure due to severe covid-19 pneumonia. Respiratory Care. 2021;66(12):1824-30. doi: 10.4187/respcare.09130.

202. Nasrallah BZN, Mahmoud MS, ElGendy HMA, Youssri Mahmoud NM, ElGendy MAEA. Patients self-proning with high-flow nasal cannula improves oxygenation in mild ARDS patients: a randomized clinical trial. Anaesthesia, Pain and Intensive Care. 2023;27(3):351-5. doi: 10.35975/apic.v27i3.2079.

203. Navarese EP, Podhajski P, Gurbel PA, Grzelakowska K, Ruscio E, Tantry U, et al. PCSK9 Inhibition During the Inflammatory Stage of SARS-CoV-2 Infection. Journal of the American College of Cardiology. 2023;81(3):224-34. doi: 10.1016/j.jacc.2022.10.030.

204. Naveena K. Comparative study of trans-nasal humidified rapid insufflation ventilator exchange vs variable flow nasal catheter in optimizing oxygenation-awake fiberoptic intubation. Anesthesia and Analgesia. 2021;133(3 SUPPL 2):440-1.

205. Nay MA, Hindre R, Perrin C, Clément J, Plantier L, Sève A, et al. Prone position versus usual care in hypoxemic COVID-19 patients in medical wards: a randomised controlled trial. Critical Care. 2023;27(1). doi: 10.1186/s13054-023-04529-z.

206. Nekhendzy V, Saxena A, Mittal B, Sun E, Sung K, Dewan K, et al. The Safety and Efficacy of Transnasal Humidified Rapid-Insufflation Ventilatory Exchange for Laryngologic Surgery. Laryngoscope. 2020;130(12):E874-E81. doi: 10.1002/lary.28562.

207. Nevola R, Russo A, Scuotto S, Imbriani S, Aprea C, Abitabile M, et al. Non-invasive respiratory support in SARS-CoV-2 related acute respiratory distress syndrome: when is it most appropriate to start treatment? Respiratory Research. 2022;23(1). doi: 10.1186/s12931-022-02258-5.

208. Ng I, Krieser R, Mezzavia P, Lee K, Tseng C, Douglas NWR, et al. The Use of Transnasal Humidified Rapid-Insufflation Ventilatory Exchange (THRIVE) for Pre-Oxygenation in Neurosurgical Patients: A Randomised Controlled Trial. Anaesthesia and Intensive Care. 2018;46(4):360-7. doi: 10.1177/0310057X1804600403.

209. Omote N, Hashimoto N, Matsuda N, Nishida K, Sakamoto K, Ando A, et al. High-Flow nasal cannula therapy for acute respiratory failure in patients with interstitial pneumonia: A retrospective observational study. American Journal of Respiratory and Critical Care Medicine. 2018;197(MeetingAbstracts).

210. Oraczewska A, Cofta S, Warcholiński A, Trejnowska E, Brożek G, Swinarew A, et al. The use of non-invasive respiratory assistance to facilitate bronchofiberoscopy performance in patients with hypoxemic (type one) respiratory failure - Study protocol. Advances in Medical Sciences. 2023;68(2):474-81. doi: 10.1016/j.advms.2023.10.011.

211. Osman A, Via G, Sallehuddin RM, Ahmad AH, Fei SK, Azil A, et al. Helmet continuous positive airway pressure vs. high flow nasal cannula oxygen in acute cardiogenic pulmonary oedema: A randomized controlled trial. European Heart Journal: Acute Cardiovascular Care. 2021;10(10):1103-11. doi: 10.1093/ehjacc/zuab078.

212. Osman YM, Abd El-Raof R. High flow nasal cannula oxygen preventing deoxygenation during induction of general anaesthesia in caesarean section: A randomized controlled trial. Trends in Anaesthesia and Critical Care. 2021;40:23-7. doi: 10.1016/j.tacc.2021.06.006.

213. Ospina-Tascón GA, Calderón-Tapia LE, García AF, Zarama V, Gómez-Álvarez F, Álvarez-Saa T, et al. Effect of High-Flow Oxygen Therapy vs Conventional Oxygen Therapy on Invasive Mechanical Ventilation and Clinical Recovery in Patients with Severe COVID-19: A Randomized Clinical Trial. JAMA. 2021;326(21):2161-71. doi: 10.1001/jama.2021.20714.

214. Papachatzakis Y, Nikolaidis PT, Kontogiannis S, Trakada G. High-flow oxygen through nasal cannula vs. Non-invasive ventilation in hypercapnic respiratory failure: A randomized clinical trial. International Journal of Environmental Research and Public Health. 2020;17(16):1-8. doi: 10.3390/ijerph17165994.

215. Papalampidou A, Bibaki E, Boutlas S, Pantazopoulos I, Athanasiou N, Moylan M, et al. Nasal high-flow oxygen versus noninvasive ventilation in acute exacerbation of copd: Protocol for a randomised noninferiority clinical trial. ERJ Open Research. 2020;6(4):1-7. doi: 10.1183/23120541.00114-2020.

216. Papi A, Stapleton RD, Shore PM, Bica MA, Chen Y, Larbig M, et al. Efficacy and Safety of Garadacimab in Combination with Standard of Care Treatment in Patients with Severe COVID-19. Lung. 2023;201(2):159-70. doi: 10.1007/s00408-023-00615-9.

217. Park S, Kim SY, Kim MS, Park WK, Byon HJ, Kim HJ. Comparison of preoxygenation efficiency measured by the oxygen reserve index between high-flow nasal oxygenation and facemask ventilation: a randomised controlled trial. BMC Anesthesiology. 2023;23(1). doi: 10.1186/s12871-023-02126-9.

218. Patel A, El-Boghdadly K. Facemask or high-flow nasal oxygenation: time to switch? Anaesthesia. 2022;77(1):7-11. doi: 10.1111/anae.15593.

219. Patsaki I, Christakou A, Papadopoulos E, Katartzi M, Kouvarakos A, Siempos I, et al. The combination of inspiratory muscle training and high-flow nasal cannula oxygen therapy for promoting weaning outcomes in difficult-to-wean patients: Protocol for a randomised controlled trial. ERJ Open Research. 2020;6(3):1-6. doi: 10.1183/23120541.00088-2020.

220. Pawar SC. Early versus Late Awake Prone positioning in COVID-19 Patients. Indian Journal of Critical Care Medicine. 2022;26:S88. doi: 10.5005/jp-journals-10071-23712A.183.

221. Perkins GD, Couper K, Connolly B, Baillie JK, Bradley JM, Dark P, et al. RECOVERY-Respiratory Support: Respiratory Strategies for patients with suspected or proven COVID-19 respiratory failure; Continuous Positive Airway Pressure, High-flow Nasal Oxygen, and standard care: A structured summary of a study protocol for a randomised controlled trial. Trials. 2020;21(1). doi: 10.1186/s13063-020-04617-3.

222. Perkins GD, Ji C, Connolly BA, Couper K, Lall R, Baillie JK, et al. Effect of Noninvasive Respiratory Strategies on Intubation or Mortality among Patients with Acute Hypoxemic Respiratory Failure and COVID-19: The RECOVERY-RS Randomized Clinical Trial. JAMA. 2022;327(6):546-58. doi: 10.1001/jama.2022.0028.

223. Pettenuzzo T, Boscolo Bozza A, Pistollato E, Pretto C, Giacon TA, Frasson S, et al. Noninvasive respiratory support to prevent re-intubation after surgery: a systematic review and network meta-analysis. Intensive Care Medicine Experimental. 2023;11. doi: 10.1186/s40635-023-00546-y.

224. Pibul W, Kocharunjitt W, Foofuengmonkolkit K. High-flow nasal cannula vs. Incentive spirometer after cardiac surgery: A randomized controlled trial. Journal of the Medical Association of Thailand. 2021;104(5):794-801. doi: 10.35755/jmedassocthai.2021.05.12120.

225. Pietzsch JB, Geisler BP, Whittle J, Kearney J, Ashe T, Garner AM, et al. Resource utilization and costs of high velocity nasal insufflation compared to non-invasive positive pressure ventilation for respiratory failure. Value in Health. 2017;20(5):A2-A3.

226. Préel MN, Theiler DML, Greif DMR, Ulmer DMF, Riva DMT. Improved ventilation during apnoea in children with THRIVE at different flow rates. Trends in Anaesthesia and Critical Care. 2020;30:e82. doi: 10.1016/j.tacc.2019.12.204.

227. Qin H, Jing GQ, Tan W, Wang J, Yin YN, Chen RZ, et al. Comparison of high-flow nasal cannula and conventional oxygen therapy for high-risk patients during bronchoscopy examination: protocol for a randomized controlled trial. Trials. 2023;24(1). doi: 10.1186/s13063-022-07001-5.

228. Rahnema A, Jones J. Non-invasive ventilation versus high-flow nasal cannula oxygen therapy with apnoeic oxygenation for preoxygenation before intubation of patients with acute hypoxaemic respiratory failure: a randomised, multicentre, open-label trial: Frat JP, Ricard JD, Quenot JP, et al. Lancet Respir Med. 2019;7:303-312. Journal of Emergency Medicine. 2019;57(3):417. doi: 10.1016/j.jemermed.2019.08.018.

229. Rajan S, Joseph N, Tosh P, Kadapamannil D, Paul J, Kumar L. Effectiveness of transnasal humidified rapid-insufflation ventilatory exchange versus traditional preoxygenation followed by apnoeic oxygenation in delaying desaturation during apnoea: A preliminary study. Indian Journal of Anaesthesia. 2018;62(3):202-7. doi: 10.4103/ija.IJA_717_17.

230. Ramnarayan P, Lister P, Dominguez T, Habibi P, Edmonds N, Canter RR, et al. FIRST-line support for Assistance in Breathing in Children (FIRST-ABC): A multicentre pilot randomised controlled trial of high-flow nasal cannula therapy versus continuous positive airway pressure in paediatric critical care. Critical Care. 2018;22(1). doi: 10.1186/s13054-018-2080-3.

231. Ramnarayan P, Richards-Belle A, Drikite L, Saull M, Orzechowska I, Darnell R, et al. Effect of High-Flow Nasal Cannula Therapy vs Continuous Positive Airway Pressure Therapy on Liberation from Respiratory Support in Acutely Ill Children Admitted to Pediatric Critical Care Units: A Randomized Clinical Trial. JAMA. 2022;328(2):162-72. doi: 10.1001/jama.2022.9615.

232. Rattanajiajaroen P, Kongpolprom N. Effects of High Flow Nasal Cannula on the Coordination between Swallowing and Breathing in Postextubation Patients. European Respiratory Journal. 2020;56. doi: 10.1183/13993003.congress-2020.4414.

233. Rattanajiajaroen P, Kongpolprom N. Effects of high flow nasal cannula on the coordination between swallowing and breathing in postextubation patients, a randomized crossover study. Critical Care. 2021;25(1). doi: 10.1186/s13054-021-03786-0.

234. Reep C, Fleuren L, Wils EJ, Heunks L. Effect of Non-invasive Respiratory Strategies on Intubation Rate in Patients with Hypoxemic Respiratory Failure with COVID-19: A Target Trial Emulation. Intensive Care Medicine Experimental. 2023;11. doi: 10.1186/s40635-023-00546-y.

235. Rejdak K, Fiedor P, Bonek R, Łukasiak J, Chełstowski W, Kiciak S, et al. Amantadine in unvaccinated patients with early, mild to moderate COVID-19: A randomized, placebo-controlled, double-blind trial. European Journal of Neurology. 2024;31(1). doi: 10.1111/ene.16045.

236. Rémi C, Jean-Pierre F, Stephan E, Frédéric P, Maxens D, Nicolas T, et al. High-flow nasal oxygen alone or alternating with noninvasive ventilation in critically ill immunocompromised patients with acute respiratory failure. A randomised controlled trial. Annals of Intensive Care. 2022;12. doi: 10.1186/s13613-022-01016-6.

237. Ricottilli F, Ickx B, Van Obbergh L. High-flow nasal cannula preoxygenation in obese patients undergoing general anaesthesia: a randomised controlled trial. British Journal of Anaesthesia. 2019;123(3):e443-e4. doi: 10.1016/j.bja.2019.05.006.

238. Riedel T, Bürgi F, Greif R, Kaiser H, Riva T, Theiler L, et al. Changes in lung volume estimated by electrical impedance tomography during apnea and high-flow nasal oxygenation: A single-center randomized controlled trial. PLoS ONE. 2022;17(9 September). doi: 10.1371/journal.pone.0273120.

239. Roberts C, Owen L, Manley B, Frøisland DH, Donath S, Dalziel K, et al. Nasal high flowas primary respiratory support for preterm infants. European Journal of Pediatrics. 2016;175(11):1479. doi: 10.1007/s00431-016-2785-8.

240. Roberts CT, Owen LS, Frøisland DH, Doyle LW, Davis PG, Manley BJ. Predictors and Outcomes of Early Intubation in Infants Born at 28-36 Weeks of Gestation Receiving Noninvasive Respiratory Support. Journal of Pediatrics. 2020;216:109-16.e1. doi: 10.1016/j.jpeds.2019.09.026.

241. Roberts CT, Owen LS, Manley BJ, Frøisland DH, Donath SM, Pritchard MA, et al. High-flow nasal cannulae as primary respiratory support for preterm infants-an international, multi-centre, randomised, controlled, non-inferiority trial. Journal of Paediatrics and Child Health. 2016;52:120. doi: 10.1111/jpc.13194.

242. Rosén J, Fors D, Frykholm P. Preoxygenation with high-flow nasal cannula versus face mask in morbidly obese patients. British Journal of Anaesthesia. 2020;125(1):e206-e7. doi: 10.1016/j.bja.2020.04.033.

243. Rosén J, Frykholm P, Fors D. High-flow nasal cannula versus face mask for preoxygenation in obese patients: A randomised controlled trial. Acta Anaesthesiologica Scandinavica. 2021;65(10):1381-9. doi: 10.1111/aas.13960.

244. Rosén J, von Oelreich E, Fors D, Jonsson Fagerlund M, Taxbro K, Skorup P, et al. Awake prone positioning in patients with hypoxemic respiratory failure due to COVID-19: the PROFLO multicenter randomized clinical trial. Critical Care. 2021;25(1). doi: 10.1186/s13054-021-03602-9.

245. Ruangsomboon O, Limsuwat C, Praphruetkit N, Monsomboon A, Chakorn T. Nasal High-flow Oxygen Versus Conventional Oxygen Therapy for Acute Severe Asthma Patients: A Pilot Randomized Controlled Trial. Academic Emergency Medicine. 2021;28(5):530-41. doi: 10.1111/acem.14187.

246. Ruangsomboon O, Praphruetkit N, Monsomboon A. Parallel-group, randomised, controlled, non-inferiority trial of high-flow nasal cannula versus non-invasive ventilation for emergency patients with acute cardiogenic pulmonary oedema: study protocol. BMJ Open. 2022;12(7). doi: 10.1136/bmjopen-2021-052761.

247. Sailo L, Lalnundiki, Sailo S, Zodinpuia M, Anusuya GS, Lalramthara I, et al. Factors associated with Mortality among COVID-19 Patients Admitted in an Intensive Care Unit at a Tertiary Care Setting: A Retrospective Study from Mizoram, India. Journal of Clinical and Diagnostic Research. 2022;16(1):UC22-UC6. doi: 10.7860/JCDR/2022/52500.15860.

248. Sakaguchi Y, Nozaki-Taguchi N, Hasegawa M, Ishibashi K, Sato Y, Isono S. Combination Therapy of High-flow Nasal Cannula and Upper-body Elevation for Postoperative Sleep-disordered Breathing: Randomized Crossover Trial. Anesthesiology. 2022;137(1):15-27. doi: 10.1097/ALN.0000000000004254.

249. Saksitthichok B, Petnak T, Songern A, Boonsarngsuk V. A prospective randomized comparative study of high-flow nasal cannula oxygen and non-invasive ventilation in hypoxemic patients undergoing diagnostic flexible bronchoscopy. Journal of Thoracic Disease. 2019;11(5):1929-39. doi: 10.21037/jtd.2019.05.02.

250. Saksitthichok B, Petnak T, So-Ngern A, Boonsarngsuk V. A prospective randomized comparative study of high-flow nasal cannula oxygen and non-invasive ventilation in hypoxemic patients undergoing diagnostic flexible bronchoscopy. Respirology. 2017;22:241. doi: 10.1111/resp.13207_398.

251. Salvarani C, Massari M, Costantini M, Merlo DF, Mariani GL, Viale P, et al. Intravenous methylprednisolone pulses in hospitalised patients with severe COVID-19 pneumonia: a double-blind, randomised, placebo-controlled trial. European Respiratory Journal. 2022;60(4). doi: 10.1183/13993003.00025-2022.

252. Schibler A. Is high-flow oxygen therapy the magic bullet? Respirology. 2016;21:7. doi: 10.1111/resp.12939-3.

253. Shaikh FA, Kodicherla VR, Ramaswamy KN, Yerra A, Sachane KB, Chirla D. RANDOMIZED CONTROLLED PILOT STUDY TO COMPARE CONTINUOUS POSITIVE AIRWAY PRESSURE MODE WITH CONTINUOUS POSITIVE AIRWAY PRESSURE PLUS PRESSURE SUPPORT MODE DURING SPONTANEOUS BREATHING TRIAL. Pediatric Critical Care Medicine. 2022;23(11). doi: 10.1097/01.pcc.0000900812.38944.0e.

254. Shang X, Wang Y. Comparison of outcomes of high-flow nasal cannula and noninvasive positive-pressure ventilation in patients with hypoxemia and various APACHE II scores after extubation. Therapeutic Advances in Respiratory Disease. 2021;15. doi: 10.1177/17534666211004235.

255. Shebl E, Embarak S. High-flow nasal oxygen therapy versus noninvasive ventilation in chronic interstitial lung disease patients with acute respiratory failure. Egyptian Journal of Chest Diseases and Tuberculosis. 2018;67(3):270-5. doi: 10.4103/ejcdt.ejcdt_33_18.

256. Shin J, Park K, Lee EH, Choi BM. Humidified High Flow Nasal Cannula versus Nasal Continuous Positive Airway Pressure as an Initial Respiratory Support in Preterm Infants with Respiratory Distress: a Randomized, Controlled Non-Inferiority Trial. Journal of Korean medical science. 2017;32(4):650-5. doi: 10.3346/jkms.2017.32.4.650.

257. Shirvani TE, Nayeri FS, Shariat M, Nafs NN, Mirjalili MR, Hosseini SN, et al. Continuous positive airway pressure or humidified high flow nasal cannula for respiratory distress syndrome: A randomized control trial among premature neonates. Iranian Journal of Neonatology. 2020;11(4):50-6. doi: 10.22038/ijn.2020.46421.1783.

258. Simon M, Frings D, Wachs C, Braune S, Kluge S. High flow nasal cannula oxygen vs. bag-valvemask for preoxygenation before intubation in patients with hypoxemic respiratory failure – A prospective randomized trial. Intensive Care Medicine Experimental. 2015;3. doi: 10.1186/2197-425X-3-S1-A934.

259. Simon M, Wachs C, Braune S, de Heer G, Frings D, Kluge S. High-flow nasal cannula versus bag-valve-mask for preoxygenation before intubation in subjects with hypoxemic respiratory failure. Respiratory Care. 2016;61(9):1160-7. doi: 10.4187/respcare.04413.

260. Singh S, Ananthan A, Nanavati R. Post-INSURE Administration of Heated Humidified High-Flow Therapy Versus Nasal Continuous Positive Airway Pressure in Preterm Infants More Than 28 Weeks Gestation with Respiratory Distress Syndrome: A Randomized Non-Inferiority Trial. Journal of Tropical Pediatrics. 2022;68(4). doi: 10.1093/tropej/fmac062.

261. Sitthikarnkha P, Samransamruajkit R, Prapphal N, Deerojanawong J, Sritippayawan S. High-flow nasal cannula versus conventional oxygen therapy in children with respiratory distress. Indian Journal of Critical Care Medicine. 2018;22(5):321-5. doi: 10.4103/ijccm.IJCCM_181_17.

262. Sivasubramaniyam S, Mathews H, Dharmarajah A. Transnasal humidified rapid-insufflation ventilatory exchange (THRIVE) for paediatric endoscopy. Trends in Anaesthesia and Critical Care. 2020;30:e131-e2. doi: 10.1016/j.tacc.2019.12.322.

263. Sjöblom A, Hedberg M, Gille A, Guerra A, Aanesen V, Forsberg IM, et al. Pre-oxygenation using high-flow nasal oxygen versus tight facemask in trauma patients undergoing emergency anaesthesia. Acta Anaesthesiologica Scandinavica. 2024;68(4):447-56. doi: 10.1111/aas.14368.

264. Smith A, Banville D, O’Rourke C, Melvin P, Batey L, Borgmann A, et al. Randomized Trial of Weight-Based Versus Fixed Limit High-Flow Nasal Cannula in Bronchiolitis. Hospital Pediatrics. 2023;13(5):387-93. doi: 10.1542/hpeds.2022-006656.

265. Soliman HAZ, Fikry DM, El-Attar AM, El Hadidy MS. High flow nasal cannula effect on pulmonary complications after major elective upper abdominal surgeries: A randomized control study. Egyptian Journal of Anaesthesia. 2022;38(1):656-64. doi: 10.1080/11101849.2022.2143175.

266. Soonsawad S, Swatesutipun B, Limrungsikul A, Nuntnarumit P. Heated Humidified High-Flow Nasal Cannula for Prevention of Extubation Failure in Preterm Infants. Indian Journal of Pediatrics. 2017;84(4):262-6. doi: 10.1007/s12098-016-2280-2.

267. Soonsawad S, Tongsawang N, Nuntnarumit P. Heated Humidified High-Flow Nasal Cannula for Weaning from Continuous Positive Airway Pressure in Preterm Infants: A Randomized Controlled Trial. Neonatology. 2016;110(3):204-9. doi: 10.1159/000446063.

268. Stéphan F, Bérard L, Rézaiguia-Delclaux S, Amaru P. High-flow nasal cannula therapy versus intermittent noninvasive ventilation in obese subjects after cardiothoracic surgery. Respiratory Care. 2017;62(9):1193-202. doi: 10.4187/respcare.05473.

269. Stone GW, Farkouh ME, Lala A, Tinuoye E, Dressler O, Moreno PR, et al. Randomized Trial of Anticoagulation Strategies for Noncritically Ill Patients Hospitalized With COVID-19. Journal of the American College of Cardiology. 2023;81(18):1747-62. doi: 10.1016/j.jacc.2023.02.041.

270. Swayampakula A, Eguchi J, Juma D, Wilson M, Ejike J. Use of external nasal dilator as an adjuvant to high-flow nasal cannula oxygen therapy in children. Critical Care Medicine. 2016;44(12):310. doi: 10.1097/01.ccm.0000509612.21549.56.

271. Tan D, Walline JH, Ling B, Xu Y, Sun J, Wang B, et al. High-flow nasal cannula oxygen therapy versus non-invasive ventilation for chronic obstructive pulmonary disease patients after extubation: A multicenter, randomized controlled trial. Critical Care. 2020;24(1). doi: 10.1186/s13054-020-03214-9.

272. Tan D, Wang B, Cao P, Wang Y, Sun J, Geng P, et al. High flow nasal cannula oxygen therapy versus non-invasive ventilation for acute exacerbations of chronic obstructive pulmonary disease with acute-moderate hypercapnic respiratory failure: a randomized controlled non-inferiority trial. Critical Care. 2024;28(1). doi: 10.1186/s13054-024-05040-9.

273. Tavernier E, McNicholas B, Pavlov I, Roca O, Perez Y, Laffey J, et al. Awake prone positioning of hypoxaemic patients with COVID-19: Protocol for a randomised controlled open-label superiority meta-trial. BMJ Open. 2020;10(11). doi: 10.1136/bmjopen-2020-041520.

274. Taylor SP, Bundy H, Smith WM, Skavroneck S, Taylor B, Kowalkowski MA. Awake prone positioning strategy for nonintubated hypoxic patients with covid-19 a pilot trial with embedded implementation evaluation. Annals of the American Thoracic Society. 2021;18(8):1360-8. doi: 10.1513/AnnalsATS.202009-1164OC.

275. Theerawit P, Natpobsuk N, Sutherasan Y. The efficacy of the Whispherflow CPAP system versus high flow nasal cannula in patients at high risk for postextubation failure. Intensive Care Medicine Experimental. 2017;5(2). doi: 10.1186/s40635-017-0151-4.

276. Thille AW, Monseau G, Coudroy R, Nay MA, Gacouin A, Decavèle M, et al. Non-invasive ventilation versus high-flow nasal oxygen for postextubation respiratory failure in ICU: a post-hoc analysis of a randomized clinical trial. Critical Care. 2021;25(1). doi: 10.1186/s13054-021-03621-6.

277. Thille AW, Muller G, Gacouin A, Coudroy R, Decavèle M, Sonneville R, et al. Effect of Postextubation High-Flow Nasal Oxygen with Noninvasive Ventilation vs High-Flow Nasal Oxygen Alone on Reintubation among Patients at High Risk of Extubation Failure: A Randomized Clinical Trial. JAMA - Journal of the American Medical Association. 2019;322(15):1465-75. doi: 10.1001/jama.2019.14901.

278. Thiruvenkatarajan V, Dharmalingam A, Arenas G, Wahba M, Liu WM, Zaw Y, et al. Effect of high-flow vs. low-flow nasal plus mouthguard oxygen therapy on hypoxaemia during sedation: a multicentre randomised controlled trial. Anaesthesia. 2022;77(1):46-53. doi: 10.1111/anae.15527.

279. Tongyoo S, Tantibundit P, Daorattanachai K, Viarasilpa T, Permpikul C, Udompanturak S. High-flow nasal oxygen cannula vs. noninvasive mechanical ventilation to prevent reintubation in sepsis: a randomized controlled trial. Annals of Intensive Care. 2021;11(1). doi: 10.1186/s13613-021-00922-5.

280. Tremey B, Squara P, de Labarre H, Ma S, Fischler M, Lawkoune JD, et al. Hands-free induction of general anesthesia: A randomised pilot study comparing usual care and high-flow nasal oxygen. Minerva Anestesiologica. 2020;86(11):1135-42. doi: 10.23736/S0375-9393.20.14456-0.

281. Tseng CW, Chao KY, Chiang CE, Liu WL, Chou WR, Wu HL, et al. The efficacy of heated humidifier high-flow nasal cannula compared with noninvasive positive-pressure ventilation in prevention of reintubation in patients with prolonged mechanical ventilation. European Respiratory Journal. 2019;54. doi: 10.1183/13993003.congress-2019.RCT5097.

282. Tsui B, Caruso T, O'Connell C, Menendez M, Fonseca A, Lawrence K, et al. Transnasal humidified rapid-insufflation ventilatory exchange improves oxygenation in pediatric bronchoscopy. Canadian Journal of Anesthesia. 2019;66(2):S169-S71. doi: 10.1007/s12630-019-01499-1.

283. Tverring J, Åkesson A, Nielsen N. Helmet continuous positive airway pressure versus high-flow nasal cannula in COVID-19: a pragmatic randomised clinical trial (COVID HELMET). Trials. 2020;21(1). doi: 10.1186/s13063-020-04863-5.

284. Uy AE, Patricio M, Permejo C. Outcomes of Hemoperfusion for Critical COVID-19 Infection in a Cardiovascular Specialty Hospital in the Philippines: A Single Center Experience. Intensive Care Medicine Experimental. 2022;10. doi: 10.1186/s40635-022-00469-0.

285. Van Der Veeken E, Manley BJ, Owen L, Kamlin O, Roberts C, Newman S, et al. Cerebral Oxygenation during Neonatal Intubation with Nasal High Flow: A Sub-Study of the SHINE Randomized Trial. Neonatology. 2023;120(4):458-64. doi: 10.1159/000529870.

286. Vitaliti G, Vitaliti MC, Finocchiaro MC, Di Stefano VA, Pavone P, Matin N, et al. Randomized comparison of helmet CPAP versus high-flow nasal cannula oxygen in pediatric respiratory distress. Respiratory Care. 2017;62(8):1036-42. doi: 10.4187/respcare.05384.

287. Vourc’h M, Asfar P, Volteau C, Bachoumas K, Clavieras N, Egreteau PY, et al. High-flow nasal cannula oxygen during endotracheal intubation in hypoxemic patients: a randomized controlled clinical trial. Intensive Care Medicine. 2015;41(9):1538-48. doi: 10.1007/s00134-015-3796-z.

288. Vourc'h M, Baud G, Feuillet F, Blanchard C, Mirallie E, Guitton C, et al. High-flow Nasal Cannulae Versus Non-invasive Ventilation for Preoxygenation of Obese Patients: The PREOPTIPOP Randomized Trial. EClinicalMedicine. 2019;13:112-9. doi: 10.1016/j.eclinm.2019.05.014.

289. Vourc'H M, Huard D, Feuillet F, Baud G, Guichoux A, Surbled M, et al. Preoxygenation in difficult airway management: High-flow oxygenation by nasal cannula versus face mask (the PREOPTIDAM study). Protocol for a single-centre randomised study. BMJ Open. 2019;9(4). doi: 10.1136/bmjopen-2018-025909.

290. Vourc'h M, Huard D, Le Penndu M, Deransy R, Surbled M, Malidin M, et al. High-flow oxygen therapy versus facemask preoxygenation in anticipated difficult airway management (PREOPTI-DAM): an open-label, single-centre, randomised controlled phase 3 trial. eClinicalMedicine. 2023;60. doi: 10.1016/j.eclinm.2023.101998.

291. Waheed S, Kapadia NN, Khan MF, Kerai SM, Raheem A, Naeem R. Randomised controlled trial to assess the effectiveness of apnoeic oxygenation in adults using low-flow or high-flow nasal cannula with head side elevation versus usual care to prevent desaturation during endotracheal intubation in the emergency department (ApOxED): Study protocol. BMJ Open. 2020;10(11). doi: 10.1136/bmjopen-2020-037964.

292. Wang Q, Xie T, Gao R, Long X, Wei J, Ye L, et al. Neutrophil-to-lymphocyte ratio is a powerful predictor of adult patients with acute respiratory distress syndrome who might benefit from corticosteroid therapy. American Journal of Translational Research. 2021;13(10):11556-70.

293. Wang W, Zhang W, Lu Y, Xu Y, Zhang Y, Shi H, et al. Comparison of the Effectiveness of Transnasal Humidified Rapid Insufflation Ventilator Exchange (THRIVE) with Facemask Pre-Oxygenation in 40 Patients 365 Years of Age Undergoing General Anaesthesia During Gastrointestinal Surgery for Intestinal Obstruction. Medical Science Monitor. 2022;28. doi: 10.12659/MSM.938168.

294. Wang Z, Xiang JW, Gao WW, Shen YZ, Zhou WJ, Chen J, et al. Comparison of clinical efficacy of two noninvasive respiratory support therapies for respiratory distress syndrome in very low birth weight preterm infants. Chinese Journal of Contemporary Pediatrics. 2018;20(8):603-7. doi: 10.7499/j.issn.1008-8830.2018.08.001.

295. Wijakprasert P, Chomchoey J. High-flow nasal cannula versus conventional oxygen therapy in post-extubation pediatric patients: A randomized controlled trial. Journal of the Medical Association of Thailand. 2018;101(10):1331-5.

296. Wong DT, Dallaire A, Singh KP, Madhusudan P, Jackson T, Singh M, et al. High-Flow Nasal Oxygen Improves Safe Apnea Time in Morbidly Obese Patients Undergoing General Anesthesia: A Randomized Controlled Trial. Anesthesia and analgesia. 2019;129(4):1130-6. doi: 10.1213/ANE.0000000000003966.

297. Woodhead DD, Lambert DK, Clark JM, Christensen RD. Comparing two methods of delivering high-flow gas therapy by nasal cannula following endotracheal extubation: A prospective, randomized, masked, crossover trial. Journal of Perinatology. 2006;26(8):481-5. doi: 10.1038/sj.jp.7211543.

298. Wu Q, Xu R, Zhou X, Wang L, Sheng C, Ding M, et al. Bolus administration of remimazolam was superior to midazolam for deep sedation in elderly patients undergoing diagnostic bronchoscopy: A randomized, double-blind, controlled trial. Medicine (United States). 2024;103(12):E37215. doi: 10.1097/MD.0000000000037215. PubMed Central PMCID: PMChengrui pharmaceuticals co ltd(China)

Jiangsu Nhwa(China)

yichang renfu pharmaceuticals co ltd(China).

299. Wu YM, Li CC, Huang SY, Su YH, Wang CW, Chen JT, et al. A Comparison of Oxygenation Efficacy between High-Flow Nasal Cannulas and Standard Facemasks during Elective Tracheal Intubation for Patients with Obesity: A Randomized Controlled Trial. Journal of Clinical Medicine. 2022;11(6). doi: 10.3390/jcm11061700.

300. Xia J, Gu S, Lei W, Zhang J, Wei H, Liu C, et al. High-flow nasal cannula versus conventional oxygen therapy in acute COPD exacerbation with mild hypercapnia: a multicenter randomized controlled trial. Critical Care. 2022;26(1). doi: 10.1186/s13054-022-03973-7.

301. Xing D, Chen L, Wang L, Jin J, Liu D, Liu H, et al. An analysis of the treatment effect of two modes of oxygenation on patients with radiation pneumonia complicated by respiratory failure. Technology and Health Care. 2022;30(4):869-80. doi: 10.3233/THC-213597.

302. Xu SX, Wu CS, Liu SY, Lu X. High-flow nasal cannula oxygen therapy and noninvasive ventilation for preventing extubation failure during weaning from mechanical ventilation assessed by lung ultrasound score: A single-center randomized study. World Journal of Emergency Medicine. 2021;12(4):274-80. doi: 10.5847/WJEM.J.1920-8642.2021.04.004.

303. Xu Z, Li X, Huang Y, Mao P, Liu X, Zhang H, et al. The efficacy of high-flow nasal cannula oxygen therapy in adult respiratory failure: A metaanalysis. Critical Care Medicine. 2016;44(12):319. doi: 10.1097/01.ccm.0000509649.65505.4b.

304. Ye M, Zhang R. Effect of Humidified High-Flow Nasal Cannula Oxygen Therapy with a Pulmonary Infection Control Window as a Ventilation Switching Indication in Combination with Atomizing Inhalation of Terbutaline on the Lung Function of Patients with Acute Exacerbation of COPD. Acta medica Okayama. 2024;78(3):271-9. doi: 10.18926/AMO/67202.

305. Yoder BA, Stoddard RA, Li M, King J, Dirnberger DR, Abbasi S. Heated, humidified high-flow nasal cannula versus nasal CPAP for respiratory support in neonates. Pediatrics. 2013;131(5):e1482-e90. doi: 10.1542/peds.2012-2742.

306. Yokoyama T, Nagata K, Tsugitomi R, Nishimura N, Kondoh Y, Tomii K. Continuous Positive Airway Pressure Versus High-flow Nasal Cannula Oxygen Therapy for Acute Hypoxemic Respiratory Failure. American Journal of Respiratory and Critical Care Medicine. 2023;207(1). doi: 10.1164/ajrccm-conference.2023.B23.

307. Yuan Z, Han X, Wang L, Xue P, Sun Y, Frerichs I, et al. Oxygen therapy delivery and body position effects measured with electrical impedance tomography. Respiratory Care. 2020;65(3):281-7. doi: 10.4187/respcare.07109.

308. Yurtseven A, Turan C, Erseven E, Saz EU. Comparison of heated humidiﬁed high-flow nasal cannula flow rates (1-L·kg·min−1 vs 2-L·kg·min −1) in the management of acute bronchiolitis. Pediatric Pulmonology. 2019;54(6):894-900. doi: 10.1002/ppul.24318.

309. Zhang W, Yin H, Xu Y, Fang Z, Wang W, Zhang C, et al. The effect of varying inhaled oxygen concentrations of high-flow nasal cannula oxygen therapy during gastroscopy with propofol sedation in elderly patients: a randomized controlled study. BMC Anesthesiology. 2022;22(1). doi: 10.1186/s12871-022-01879-z.

310. Zhang W, Yuan X, Shen Y, Wang J, Xie K. Optimal flow of high-flow nasal cannula oxygenation to prevent desaturation during sedation for bronchoscopy: a randomized controlled study. Therapeutic Advances in Respiratory Disease. 2024;18. doi: 10.1177/17534666241246637.

311. Zhao Y, Zhang Y, Huang T, Ding Y, Tao Y, Gao J. Effect of transnasal humidified rapid insufflation ventilatory exchange on cerebral oxygen saturation during induction of general anesthesia in patients undergoing traumatic brain injury emergency surgery. Chinese Critical Care Medicine. 2024;36(4):404-9. doi: 10.3760/cma.j.cn121430-20230911-00774.

312. Zhong M, Xia R, Zhou J, Zhang J, Yi X, Yang A. The comparison of preoxygenation methods before endotracheal intubation: a network meta-analysis of randomized trials. Frontiers in Medicine. 2024;11. doi: 10.3389/fmed.2024.1379369.

313. Zhou R, Xiong T, Tang J, Huang Y, Liu W, Zhu J, et al. High-flow nasal cannula (HFNC) vs continuous positive airway pressure (CPAP) vs nasal intermittent positive pressure ventilation as primary respiratory support in infants of ≥ 32 weeks gestational age (GA): study protocol for a three-arm multi-center randomized controlled trial. Trials. 2023;24(1). doi: 10.1186/s13063-023-07665-7.

314. Zhou S, Zhou Y, Cao X, Ni X, Du W, Xu Z, et al. The efficacy of high flow nasal oxygenation for maintaining maternal oxygenation during rapid sequence induction in pregnancy: A prospective randomised clinical trial. European Journal of Anaesthesiology. 2021;38(10):1052-8. doi: 10.1097/EJA.0000000000001395.

315. Zhou X, Huang X, Zhou Z, Xu Q, Mei A, Mazomba LX, et al. Effect of transnasal humidified rapid-insufflation ventilatory exchange on gastric insufflation during anaesthesia induction A randomised controlled trial and multivariate analysis. European Journal of Anaesthesiology. 2023;40(7):521-8. doi: 10.1097/EJA.0000000000001846.

316. Zhou X, Luo X, Li Q, Chen G, Tong J, Deng W. Prone versus lateral position in acute hypoxemic respiratory failure patients with HFNO therapy: study protocol for a multicentre randomised controlled open-label trial. Trials. 2023;24(1). doi: 10.1186/s13063-023-07761-8.

317. Zhou Y, Shi X, Pu Z, Liu A. Clinical effect of high-flow nasal cannula oxygen therapy combined with naloxone on severe respiratory failure in older adult patients: a randomized controlled trial. American Journal of Translational Research. 2023;15(11):6613-20.

318. Zhu Q, Wang B, Li Y, Ling B, Xu J, Jin K, et al. High-flow nasal cannula oxygen therapy versus noninvasive ventilation for patients with blunt chest trauma: protocol for a randomized controlled trial. Trials. 2022;23(1). doi: 10.1186/s13063-022-06507-2.

319. Zhu Z, Liu Y, Wang Q, Wang S. Preliminary evaluation of sequential therapy by high flow nasal cannula oxygen therapy following endotracheal tube extubation in mechanically ventilated patients. Chinese Critical Care Medicine. 2017;29(9):778-82. doi: 10.3760/cma.j.issn.2095-4352.2017.09.003.

Pubmed:

1. Al Hashim AH, Al Reesi A, Al Lawati NM, Burad J, Al Khabori M, Chandwani J, et al. Comparison of Noninvasive Mechanical Ventilation With High-Flow Nasal Cannula, Face-Mask, and Helmet in Hypoxemic Respiratory Failure in Patients With COVID-19: A Randomized Controlled Trial. Crit Care Med. 2023;51(11):1515-26. Epub 2023/06/13. doi: 10.1097/ccm.0000000000005963. PubMed PMID: 37310174; PubMed Central PMCID: PMCPMC10563904 Al Masroori, Mr. Al Siyabi, Dr. F. Al Lawati, Dr. Ahmed, Dr. Al Huraizi, Ms. Al Zaabi, Mr. Sebastian, and Dr. Al Busaidy received funding and support for this article research from the Medical Research Center, Sultan Qaboos University (EG/DVC/MRC/20/04). Dr. Al Reesi, Ms. Varghese, and Mr. Al Abri disclosed government work. The remaining authors have disclosed that they do not have any potential conflicts of interest.

2. Andino R, Vega G, Pacheco SK, Arevalillo N, Leal A, Fernández L, et al. High-flow nasal oxygen reduces endotracheal intubation: a randomized clinical trial. Ther Adv Respir Dis. 2020;14:1753466620956459. Epub 2020/09/26. doi: 10.1177/1753466620956459. PubMed PMID: 32976085; PubMed Central PMCID: PMCPMC7522841.

3. Badruddin SS, Clayton JA, McKee BP, Slain KN, Rotta AT, Malay S, et al. Prevalence of Reintubation Within 24 Hours of Extubation in Bronchiolitis: Retrospective Cohort Study Using the Virtual Pediatric Systems Database. Pediatric critical care medicine : a journal of the Society of Critical Care Medicine and the World Federation of Pediatric Intensive and Critical Care Societies. 2021;22(5):474-82. Epub 2020/10/09. doi: 10.1097/pcc.0000000000002581. PubMed PMID: 33031349.

4. Cai Q, Ma W, Wu C, Liu H, Wang S, Zhang G. [Is pre-oxygenation with high-flow nasal oxygen safe? randomized control trial of 56 cases of elderly patients during induction of general anesthesia with endotracheal intubation]. Nan Fang Yi Ke Da Xue Xue Bao. 2022;42(7):1069-74. Epub 2022/07/24. doi: 10.12122/j.issn.1673-4254.2022.07.16. PubMed PMID: 35869772; PubMed Central PMCID: PMCPMC9308868.

5. Carrié C, Rieu B, Benard A, Trin K, Petit L, Massri A, et al. Early non-invasive ventilation and high-flow nasal oxygen therapy for preventing endotracheal intubation in hypoxemic blunt chest trauma patients: the OptiTHO randomized trial. Critical care (London, England). 2023;27(1):163. Epub 2023/04/27. doi: 10.1186/s13054-023-04429-2. PubMed PMID: 37101272; PubMed Central PMCID: PMCPMC10131545.

6. Casey JD, Vaughan EM, Lloyd BD, Billas PA, Jackson KE, Hall EJ, et al. Protocolized Postextubation Respiratory Support to Prevent Reintubation: A Randomized Clinical Trial. Am J Respir Crit Care Med. 2021;204(3):294-302. Epub 2021/04/02. doi: 10.1164/rccm.202009-3561OC. PubMed PMID: 33794131; PubMed Central PMCID: PMCPMC8513595.

7. Cho JY, Kim HS, Kang H, Kim SH, Choe KH, Lee KM, et al. Comparison of Postextubation Outcomes Associated with High-Flow Nasal Cannula vs. Conventional Oxygen Therapy in Patients at High Risk of Reintubation: a Randomized Clinical Trial. J Korean Med Sci. 2020;35(25):e194. Epub 2020/07/01. doi: 10.3346/jkms.2020.35.e194. PubMed PMID: 32597041; PubMed Central PMCID: PMCPMC7324267.

8. Chua MT, Ng WM, Lu Q, Low MJW, Punyadasa A, Cove ME, et al. Pre- and apnoeic high-flow oxygenation for rapid sequence intubation in the emergency department (the Pre-AeRATE trial): A multicentre randomised controlled trial. Ann Acad Med Singap. 2022;51(3):149-60. Epub 2022/04/05. doi: 10.47102/annals-acadmedsg.2021407. PubMed PMID: 35373238.

9. Cırıl MF, Akarca M, Unal Akoglu E, Cimilli Ozturk T, Onur Ö. High-Flow Nasal Cannula versus Bag Valve Mask for Preoxygenation during Rapid Sequence Intubation in the Emergency Department: A Single-Center, Prospective, Randomized Controlled Trial. Prehosp Disaster Med. 2024;39(1):45-51. Epub 2023/12/18. doi: 10.1017/s1049023x23006684. PubMed PMID: 38108139.

10. Claassen CC, Hillman NH, Brown K, Williams HL, Strand ML. Comparison of Bubble CPAP Devices Using RAM Cannula for Extubation Failure in Very Low Birth Weight Infants: Randomized and Cohort Studies. Neonatology. 2019;115(1):28-35. Epub 2018/10/03. doi: 10.1159/000493156. PubMed PMID: 30278459.

11. De Rosa S, Messina A, Sorbello M, Rigobello A, Colombo D, Piccolo A, et al. Laryngeal Mask Airway Supreme vs. the Spritztube tracheal cannula in anaesthetised adult patients: A randomised controlled trial. Eur J Anaesthesiol. 2019;36(12):955-62. Epub 2019/10/24. doi: 10.1097/eja.0000000000001106. PubMed PMID: 31644512.

12. Frat JP, Ragot S, Coudroy R, Constantin JM, Girault C, Prat G, et al. Predictors of Intubation in Patients With Acute Hypoxemic Respiratory Failure Treated With a Noninvasive Oxygenation Strategy. Crit Care Med. 2018;46(2):208-15. Epub 2017/11/04. doi: 10.1097/ccm.0000000000002818. PubMed PMID: 29099420.

13. Frat JP, Ragot S, Girault C, Perbet S, Prat G, Boulain T, et al. Effect of non-invasive oxygenation strategies in immunocompromised patients with severe acute respiratory failure: a post-hoc analysis of a randomised trial. The Lancet Respiratory medicine. 2016;4(8):646-52. Epub 2016/06/02. doi: 10.1016/s2213-2600(16)30093-5. PubMed PMID: 27245914.

14. Frat JP, Ricard JD, Quenot JP, Pichon N, Demoule A, Forel JM, et al. Non-invasive ventilation versus high-flow nasal cannula oxygen therapy with apnoeic oxygenation for preoxygenation before intubation of patients with acute hypoxaemic respiratory failure: a randomised, multicentre, open-label trial. The Lancet Respiratory medicine. 2019;7(4):303-12. Epub 2019/03/23. doi: 10.1016/s2213-2600(19)30048-7. PubMed PMID: 30898520.

15. Futier E, Paugam-Burtz C, Constantin JM, Pereira B, Jaber S. The OPERA trial - comparison of early nasal high flow oxygen therapy with standard care for prevention of postoperative hypoxemia after abdominal surgery: study protocol for a multicenter randomized controlled trial. Trials. 2013;14:341. Epub 2013/10/22. doi: 10.1186/1745-6215-14-341. PubMed PMID: 24138710; PubMed Central PMCID: PMCPMC3854478.

16. Grieco DL, Menga LS, Cesarano M, Rosà T, Spadaro S, Bitondo MM, et al. Effect of Helmet Noninvasive Ventilation vs High-Flow Nasal Oxygen on Days Free of Respiratory Support in Patients With COVID-19 and Moderate to Severe Hypoxemic Respiratory Failure: The HENIVOT Randomized Clinical Trial. Jama. 2021;325(17):1731-43. Epub 2021/03/26. doi: 10.1001/jama.2021.4682. PubMed PMID: 33764378; PubMed Central PMCID: PMCPMC7995134 Italian Society of Anesthesia, Analgesia, and Intensive Care Medicine during the conduct of the study and grants from the European Society of Intensive Care Medicine and GE Healthcare and travel expenses from Maquet, Getinge, and Air Liquide outside the submitted work. Dr Montomoli reported receiving personal fees from Active Medica BV outside the submitted work. Dr Conti reported receiving payments for lectures from Chiesi Pharmaceuticals SpA. Dr Maggiore reported serving as the principal investigator of the RINO trial (ClinicalTrials.gov NCT02107183), which was supported by Fisher and Paykel Healthcare through an institutional grant, and receiving personal fees from Draeger Medical and GE Healthcare outside the submitted work. Dr Antonelli reported receiving personal fees from Maquet, Chiesi, and Air Liquide and grants from GE Healthcare outside the submitted work. No other disclosures were reported.

17. Guitton C, Ehrmann S, Volteau C, Colin G, Maamar A, Jean-Michel V, et al. Nasal high-flow preoxygenation for endotracheal intubation in the critically ill patient: a randomized clinical trial. Intensive Care Med. 2019;45(4):447-58. Epub 2019/01/23. doi: 10.1007/s00134-019-05529-w. PubMed PMID: 30666367.

18. Hagan KB, Coronel E, Ge P, Hagberg C. A randomized controlled trial of the LMA® Gastro™ compared to nasal cannula for endoscopic retrograde cholangiopancreatography. Anaesthesia, critical care & pain medicine. 2024;43(4):101379. Epub 2024/03/21. doi: 10.1016/j.accpm.2024.101379. PubMed PMID: 38508391.

19. Hernández G, Paredes I, Moran F, Buj M, Colinas L, Rodríguez ML, et al. Effect of postextubation noninvasive ventilation with active humidification vs high-flow nasal cannula on reintubation in patients at very high risk for extubation failure: a randomized trial. Intensive Care Med. 2022;48(12):1751-9. Epub 2022/11/19. doi: 10.1007/s00134-022-06919-3. PubMed PMID: 36400984; PubMed Central PMCID: PMCPMC9676812.

20. Hernández G, Vaquero C, González P, Subira C, Frutos-Vivar F, Rialp G, et al. Effect of Postextubation High-Flow Nasal Cannula vs Conventional Oxygen Therapy on Reintubation in Low-Risk Patients: A Randomized Clinical Trial. Jama. 2016;315(13):1354-61. Epub 2016/03/16. doi: 10.1001/jama.2016.2711. PubMed PMID: 26975498.

21. Hua Z, Liu Z, Li Y, Zhang H, Yang M, Zuo M. Transnasal humidified rapid insufflation ventilatory exchange vs. facemask oxygenation in elderly patients undergoing general anaesthesia: a randomized controlled trial. Sci Rep. 2020;10(1):5745. Epub 2020/04/03. doi: 10.1038/s41598-020-62716-2. PubMed PMID: 32238855; PubMed Central PMCID: PMCPMC7113239.

22. Ibarra-Estrada M, Marín-Rosales M, García-Salcido R, Aguirre-Díaz SA, Vargas-Obieta A, Chávez-Peña Q, et al. Prone positioning in non-intubated patients with COVID-19 associated acute respiratory failure, the PRO-CARF trial: A structured summary of a study protocol for a randomised controlled trial. Trials. 2020;21(1):940. Epub 2020/11/24. doi: 10.1186/s13063-020-04882-2. PubMed PMID: 33225990; PubMed Central PMCID: PMCPMC7680981.

23. Jaber S, Molinari N, De Jong A. New method of preoxygenation for orotracheal intubation in patients with hypoxaemic acute respiratory failure in the intensive care unit, non-invasive ventilation combined with apnoeic oxygenation by high flow nasal oxygen: the randomised OPTINIV study protocol. BMJ Open. 2016;6(8):e011298. Epub 2016/08/16. doi: 10.1136/bmjopen-2016-011298. PubMed PMID: 27519921; PubMed Central PMCID: PMCPMC4985915.

24. Jaber S, Monnin M, Girard M, Conseil M, Cisse M, Carr J, et al. Apnoeic oxygenation via high-flow nasal cannula oxygen combined with non-invasive ventilation preoxygenation for intubation in hypoxaemic patients in the intensive care unit: the single-centre, blinded, randomised controlled OPTINIV trial. Intensive Care Med. 2016;42(12):1877-87. Epub 2016/10/13. doi: 10.1007/s00134-016-4588-9. PubMed PMID: 27730283.

25. Jo JY, Kim WJ, Ku S, Choi SS. Comparison of preoxygenation with a high-flow nasal cannula and a simple mask before intubation during induction of general anesthesia in patients undergoing head and neck surgery: Study protocol clinical trial (SPIRIT Compliant). Medicine (Baltimore). 2020;99(12):e19525. Epub 2020/03/21. doi: 10.1097/md.0000000000019525. PubMed PMID: 32195955; PubMed Central PMCID: PMCPMC7220443.

26. King BJ, Megison A, Scogin Z, Christensen BJ. Capnography Detection Using Nasal Cannula Is Superior to Modified Nasal Hood in an Open Airway System: A Randomized Controlled Trial. Journal of oral and maxillofacial surgery : official journal of the American Association of Oral and Maxillofacial Surgeons. 2019;77(8):1576-81. Epub 2019/03/10. doi: 10.1016/j.joms.2019.02.002. PubMed PMID: 30851253.

27. Lemiale V, Resche-Rigon M, Mokart D, Pène F, Argaud L, Mayaux J, et al. High-Flow Nasal Cannula Oxygenation in Immunocompromised Patients With Acute Hypoxemic Respiratory Failure: A Groupe de Recherche Respiratoire en Réanimation Onco-Hématologique Study. Crit Care Med. 2017;45(3):e274-e80. Epub 2016/09/23. doi: 10.1097/ccm.0000000000002085. PubMed PMID: 27655324.

28. Li J, Liu B, Zhou QH, Ni HD, Liu MJ, Deng K. Pre-oxygenation with high-flow oxygen through the nasopharyngeal airway compared to facemask on carbon dioxide clearance in emergency adults: a prospective randomized non-blinded clinical trial. Eur J Trauma Emerg Surg. 2024;50(3):1051-61. Epub 2023/12/27. doi: 10.1007/s00068-023-02418-2. PubMed PMID: 38148421; PubMed Central PMCID: PMCPMC11249433.

29. Li XQ, Zhao WL, Li DY, Lei L, Luo LL, Qiao LN. [Clinical Study on Early Extubation and Sequential Non-Invasive Respiratory Support for Children with Acute Respiratory Failure Receiving Invasive Mechanical Ventilation]. Sichuan da xue xue bao Yi xue ban = Journal of Sichuan University Medical science edition. 2022;53(2):321-6. Epub 2022/03/26. doi: 10.12182/20220360206. PubMed PMID: 35332737; PubMed Central PMCID: PMCPMC10409362.

30. Lodenius Å, Piehl J, Östlund A, Ullman J, Jonsson Fagerlund M. Transnasal humidified rapid-insufflation ventilatory exchange (THRIVE) vs. facemask breathing pre-oxygenation for rapid sequence induction in adults: a prospective randomised non-blinded clinical trial. Anaesthesia. 2018;73(5):564-71. Epub 2018/01/14. doi: 10.1111/anae.14215. PubMed PMID: 29330853.

31. Maggiore SM, Jaber S, Grieco DL, Mancebo J, Zakynthinos S, Demoule A, et al. High-Flow Versus VenturiMask Oxygen Therapy to Prevent Reintubation in Hypoxemic Patients after Extubation: A Multicenter Randomized Clinical Trial. Am J Respir Crit Care Med. 2022;206(12):1452-62. Epub 2022/07/19. doi: 10.1164/rccm.202201-0065OC. PubMed PMID: 35849787.

32. Makdee O, Monsomboon A, Surabenjawong U, Praphruetkit N, Chaisirin W, Chakorn T, et al. High-Flow Nasal Cannula Versus Conventional Oxygen Therapy in Emergency Department Patients With Cardiogenic Pulmonary Edema: A Randomized Controlled Trial. Ann Emerg Med. 2017;70(4):465-72.e2. Epub 2017/06/12. doi: 10.1016/j.annemergmed.2017.03.028. PubMed PMID: 28601264.

33. Matsuda W, Hagiwara A, Uemura T, Sato T, Kobayashi K, Sasaki R, et al. High-Flow Nasal Cannula May Not Reduce the Re-Intubation Rate Compared With a Large-Volume Nebulization-Based Humidifier. Respir Care. 2020;65(5):610-7. Epub 2020/01/30. doi: 10.4187/respcare.07095. PubMed PMID: 31992669.

34. McQuade D, Miller MR, Hayes-Bradley C. Addition of Nasal Cannula Can Either Impair or Enhance Preoxygenation With a Bag Valve Mask: A Randomized Crossover Design Study Comparing Oxygen Flow Rates. Anesth Analg. 2018;126(4):1214-8. Epub 2017/08/02. doi: 10.1213/ane.0000000000002341. PubMed PMID: 28759496.

35. Miller SM, Dowd SA. High-flow nasal cannula and extubation success in the premature infant: a comparison of two modalities. Journal of perinatology : official journal of the California Perinatal Association. 2010;30(12):805-8. Epub 2010/03/20. doi: 10.1038/jp.2010.38. PubMed PMID: 20237485.

36. Min SH, Yoon H, Huh G, Kwon SK, Seo JH, Cho YJ. Efficacy of high-flow nasal oxygenation compared with tracheal intubation for oxygenation during laryngeal microsurgery: a randomised non-inferiority study. Br J Anaesth. 2022;128(1):207-13. Epub 2021/10/25. doi: 10.1016/j.bja.2021.09.016. PubMed PMID: 34688473.

37. Mir F, Patel A, Iqbal R, Cecconi M, Nouraei SA. A randomised controlled trial comparing transnasal humidified rapid insufflation ventilatory exchange (THRIVE) pre-oxygenation with facemask pre-oxygenation in patients undergoing rapid sequence induction of anaesthesia. Anaesthesia. 2017;72(4):439-43. Epub 2016/12/31. doi: 10.1111/anae.13799. PubMed PMID: 28035669.

38. Mosier J, Reardon RF, DeVries PA, Stang JL, Nelsen A, Prekker ME, et al. Time to Loss of Preoxygenation in Emergency Department Patients. The Journal of emergency medicine. 2020;59(5):637-42. Epub 2020/08/11. doi: 10.1016/j.jemermed.2020.06.064. PubMed PMID: 32771321.

39. Ng I, Krieser R, Mezzavia P, Lee K, Tseng C, Douglas N, et al. The use of Transnasal Humidified Rapid-Insufflation Ventilatory Exchange (THRIVE) for pre-oxygenation in neurosurgical patients: a randomised controlled trial. Anaesth Intensive Care. 2018;46(4):360-7. Epub 2018/07/03. doi: 10.1177/0310057x1804600403. PubMed PMID: 29966108.

40. Perkins GD, Ji C, Connolly BA, Couper K, Lall R, Baillie JK, et al. Effect of Noninvasive Respiratory Strategies on Intubation or Mortality Among Patients With Acute Hypoxemic Respiratory Failure and COVID-19: The RECOVERY-RS Randomized Clinical Trial. Jama. 2022;327(6):546-58. Epub 2022/01/25. doi: 10.1001/jama.2022.0028. PubMed PMID: 35072713; PubMed Central PMCID: PMCPMC8787685 National Institute for Health Research (NIHR) West Midlands Applied Research Collaboration and serving as co-director of research for the Intensive Care Society until recently (term ended in June 2021). Dr Connolly reported receiving grants from the NIHR; receiving personal fees from Fisher & Paykel Healthcare; and serving as the director of research for the Intensive Care Society. Dr Baillie reported receiving grants from the Wellcome Trust, the Biotechnology and Biological Sciences Research Council, and the Medical Research Council. Dr Dark reported receiving grants from the Manchester NIHR Biomedical Research Centre and being a national specialty cluster lead for the NIHR. Dr Dave reported receiving personal fees from Chiesi. Dr De Soyza reported being a national specialty cluster lead for the NIHR and receiving personal fees from AstraZeneca, Bayer, Chiesi, Gilead, GlaxoSmithKline, Forest Labs, Novartis, Insmed, Teva, Zambon, and Pfizer. Mrs Devrell reported receiving personal fees from the NIHR. Dr Gorman reported receiving grants from the NIHR and Wellcome Trust. Dr Hart reported receiving a UK Research and Innovation grant from the Medical Research Council; receiving unrestricted grants and equipment from Philips-Respironics, Fisher & Paykel Healthcare, and ResMed; receiving institutional funding for his role on the Philips Global medical advisory board; receiving personal fees from Philips-Respironics, Philips, ResMed, and Fisher & Paykel Healthcare; and receiving financial support from Philips for the development of the Myotrace technology that has a patent approved in Europe and in the US. Dr Hee reported receiving grants from the British Heart Foundation and the NIHR West Midlands Research Design Service. Mr Messer reported receiving personal fees from Fisher & Paykel Healthcare. Dr Parekh reported receiving a UK Research and Innovation grant from the Medical Research Council and receiving grants from the NIHR. Dr Steiner reported receiving personal fees from GlaxoSmithKline. Dr McAuley reported receiving personal fees from GlaxoSmithKline, Boehringer Ingelheim, Bayer, Novartis, Sobi, Eli Lilly, Vir Biotechnology, and Faron Pharmaceuticals; receiving grants from the NIHR, Randox, Wellcome Trust, Innovate UK, the Medical Research Council, and the Northern Ireland Health and Social Research and Development Division; holding a patent for an anti-inflammatory treatment issued to Queen’s University Belfast; and serving as co-director of research for the Intensive Care Society until recently (term ended in June 2021) and as program director for the NIHR Efficacy and Mechanism Evaluation program. No other disclosures were reported.

41. Rosén J, von Oelreich E, Fors D, Jonsson Fagerlund M, Taxbro K, Skorup P, et al. Awake prone positioning in patients with hypoxemic respiratory failure due to COVID-19: the PROFLO multicenter randomized clinical trial. Critical care (London, England). 2021;25(1):209. Epub 2021/06/16. doi: 10.1186/s13054-021-03602-9. PubMed PMID: 34127046; PubMed Central PMCID: PMCPMC8200797.

42. Semler MW, Janz DR, Lentz RJ, Matthews DT, Norman BC, Assad TR, et al. Randomized Trial of Apneic Oxygenation during Endotracheal Intubation of the Critically Ill. Am J Respir Crit Care Med. 2016;193(3):273-80. Epub 2015/10/02. doi: 10.1164/rccm.201507-1294OC. PubMed PMID: 26426458; PubMed Central PMCID: PMCPMC4803060.

43. Shang X, Wang Y. Comparison of outcomes of high-flow nasal cannula and noninvasive positive-pressure ventilation in patients with hypoxemia and various APACHE II scores after extubation. Ther Adv Respir Dis. 2021;15:17534666211004235. Epub 2021/03/31. doi: 10.1177/17534666211004235. PubMed PMID: 33781130; PubMed Central PMCID: PMCPMC8013886.

44. Simon M, Wachs C, Braune S, de Heer G, Frings D, Kluge S. High-Flow Nasal Cannula Versus Bag-Valve-Mask for Preoxygenation Before Intubation in Subjects With Hypoxemic Respiratory Failure. Respir Care. 2016;61(9):1160-7. Epub 2016/06/09. doi: 10.4187/respcare.04413. PubMed PMID: 27274092.

45. Sjöblom A, Broms J, Hedberg M, Lodenius Å, Furubacke A, Henningsson R, et al. Pre-oxygenation using high-flow nasal oxygen vs. tight facemask during rapid sequence induction. Anaesthesia. 2021;76(9):1176-83. Epub 2021/02/19. doi: 10.1111/anae.15426. PubMed PMID: 33599993.

46. Srinet P, Van Daele DJ, Adam SI, Burrell MI, Aronberg R, Leder SB. A Biomechanical Study of Hyoid Bone and Laryngeal Movements During Swallowing Comparing the Blom Low Profile Voice Inner Cannula and Passy-Muir One Way Tracheotomy Tube Speaking Valves. Dysphagia. 2015;30(6):723-9. Epub 2015/09/18. doi: 10.1007/s00455-015-9649-7. PubMed PMID: 26376918.

47. Theerawit P, Natpobsuk N, Petnak T, Sutherasan Y. The efficacy of the WhisperFlow CPAP system versus high flow nasal cannula in patients at risk for postextubation failure: A Randomized controlled trial. J Crit Care. 2021;63:117-23. Epub 2020/10/06. doi: 10.1016/j.jcrc.2020.09.031. PubMed PMID: 33012589.

48. Thille AW, Muller G, Gacouin A, Coudroy R, Decavèle M, Sonneville R, et al. Effect of Postextubation High-Flow Nasal Oxygen With Noninvasive Ventilation vs High-Flow Nasal Oxygen Alone on Reintubation Among Patients at High Risk of Extubation Failure: A Randomized Clinical Trial. Jama. 2019;322(15):1465-75. Epub 2019/10/03. doi: 10.1001/jama.2019.14901. PubMed PMID: 31577036; PubMed Central PMCID: PMCPMC6802261 French Ministry of Health and personal fees and nonfinancial support from Fisher & Paykel Healthcare during the conduct of the study and personal fees from Maquet-Getinge, GE Healthcare, and Covidien outside the submitted work. Dr Sonneville reported receiving grants from the French Ministry of Health, the European Society of Intensive Care Medicine, and the French Society of Intensive Care Medicine and personal fees from Baxter outside the submitted work. Dr Beloncle reported receiving personal fees from Lowenstein Medical and nonfinancial support from GE Healthcare, Getinge Group, and Covidien outside the submitted work. Dr Girault reported receiving grants, personal fees, and nonfinancial support from Fisher & Paykel Healthcare during the conduct of the study and grants and nonfinancial support from ResMed outside the submitted work. Dr Ricard reported receiving travel and accommodation expenses from Fisher & Paykel Healthcare outside the submitted work. Dr Ehrmann reported receiving grants, nonfinancial support, and other funding from Fisher & Paykel Healthcare during the conduct of the study; grants, personal fees, nonfinancial support, and other funding from Aerogen; grants from Hamilton; personal fees from La Diffusion Technique Française; and personal fees from Baxter outside the submitted work. In addition, Dr Ehrmann had a patent to EP17305015 issued. Dr Terzi reported receiving personal fees from Boehringer Ingelheim and Pfizer outside the submitted work. Dr Danin reported receiving fees for lectures from Fisher and Paykel during the conduct of the study. Dr Deye reported receiving lecture and travel fees from Zoll and Bard outside the submitted work. Dr Demoule reported receiving personal fees from Medtronic, Baxter, Hamilton, and Getinge; grants, personal fees, and nonfinancial support from Philips and Lungpacer; personal fees and nonfinancial support from Fisher & Paykel Healthcare; and grants from the French Ministry of Health and Respinor outside the submitted work. Dr Frat reported receiving personal fees and nonfinancial support from Fisher & Paykel Healthcare during the conduct of the study and personal fees and nonfinancial support from SOS Oxygen outside the submitted work. No other disclosures were reported.

49. Tiruvoipati R, Lewis D, Haji K, Botha J. High-flow nasal oxygen vs high-flow face mask: a randomized crossover trial in extubated patients. J Crit Care. 2010;25(3):463-8. Epub 2009/09/29. doi: 10.1016/j.jcrc.2009.06.050. PubMed PMID: 19781896.

50. Vourc'h M, Asfar P, Volteau C, Bachoumas K, Clavieras N, Egreteau PY, et al. High-flow nasal cannula oxygen during endotracheal intubation in hypoxemic patients: a randomized controlled clinical trial. Intensive Care Med. 2015;41(9):1538-48. Epub 2015/04/15. doi: 10.1007/s00134-015-3796-z. PubMed PMID: 25869405.

51. Wang W, Zhang W, Lu Y, Xu Y, Zhang Y, Shi H, et al. Comparison of the Effectiveness of Transnasal Humidified Rapid Insufflation Ventilator Exchange (THRIVE) with Facemask Pre-Oxygenation in 40 Patients ≥65 Years of Age Undergoing General Anaesthesia During Gastrointestinal Surgery for Intestinal Obstruction. Medical science monitor : international medical journal of experimental and clinical research. 2022;28:e938168. Epub 2022/12/04. doi: 10.12659/msm.938168. PubMed PMID: 36461619; PubMed Central PMCID: PMCPMC9727993.

52. Wong DT, Dallaire A, Singh KP, Madhusudan P, Jackson T, Singh M, et al. High-Flow Nasal Oxygen Improves Safe Apnea Time in Morbidly Obese Patients Undergoing General Anesthesia: A Randomized Controlled Trial. Anesth Analg. 2019;129(4):1130-6. Epub 2019/10/05. doi: 10.1213/ane.0000000000003966. PubMed PMID: 31584919.

53. Woodhead DD, Lambert DK, Clark JM, Christensen RD. Comparing two methods of delivering high-flow gas therapy by nasal cannula following endotracheal extubation: a prospective, randomized, masked, crossover trial. Journal of perinatology : official journal of the California Perinatal Association. 2006;26(8):481-5. Epub 2006/05/26. doi: 10.1038/sj.jp.7211543. PubMed PMID: 16724119.

54. Yurtseven A, Turan C, Erseven E, Saz EU. Comparison of heated humidiﬁed high-flow nasal cannula flow rates (1-L·kg·min(-1) vs 2-L·kg·min (-1) ) in the management of acute bronchiolitis. Pediatr Pulmonol. 2019;54(6):894-900. Epub 2019/03/20. doi: 10.1002/ppul.24318. PubMed PMID: 30887731; PubMed Central PMCID: PMCPMC7167921.

55. Zhang C, Liao H, Li H, Xu Y, Zhang G, Wang X, et al. [THRIVE combined with i-gel laryngeal mask for prolonging apneic oxygenation time in retrograde intrarenal surgery]. Nan Fang Yi Ke Da Xue Xue Bao. 2023;43(9):1599-605. Epub 2023/10/10. doi: 10.12122/j.issn.1673-4254.2023.09.18. PubMed PMID: 37814875; PubMed Central PMCID: PMCPMC10563095.

56. Zhang J, Ong S, Toh H, Chew M, Ang H, Goh S. Success and Time to Oxygen Delivery for Scalpel-Finger-Cannula and Scalpel-Finger-Bougie Front-of-Neck Access: A Randomized Crossover Study With a Simulated "Can't Intubate, Can't Oxygenate" Scenario in a Manikin Model With Impalpable Neck Anatomy. Anesth Analg. 2022;135(2):376-84. Epub 2022/03/05. doi: 10.1213/ane.0000000000005969. PubMed PMID: 35245225.

Web of science:

1. Abiodun MT, Ochaba EA, Kpongo-Ogieva A. Bedside critical care training: A quasi-experimental study in the paediatric emergency department of a referral hospital in Nigeria. Southern African Journal of Critical Care. 2024;40(1):31-6. doi: 10.7196/SAJCC.2024.v40i1.1141. PubMed PMID: WOS:001222111600007.

2. Abouleish AE, Chung DH, Cohen M. Caudal anesthesia for vascular access procedures in two extremely small premature neonates. Pediatric Surgery International. 2005;21(9):749-51. doi: 10.1007/s00383-005-1474-x. PubMed PMID: WOS:000232722300017.

3. Acquisto NM, Mosier JM, Bittner EA, Patanwala AE, Hirsch KG, Hargwood P, et al. Society of Critical Care Medicine Clinical Practice Guidelines for Rapid Sequence Intubation in the Critically Ill Adult Patient. Critical Care Medicine. 2023;51(10):1411-30. doi: 10.1097/ccm.0000000000006000. PubMed PMID: WOS:001070755300017.

4. Agmy G, Adam M, Hsanen EHM, Mahmoud MA. High-flow nasal cannula versus noninvasive ventilation in the prevention of escalation to invasive mechanical ventilation in patients with acute hypoxemic respiratory failure. Egyptian Journal of Chest Diseases and Tuberculosis. 2022;71(1):81-7. doi: 10.4103/ecdt.ecdt_12_20. PubMed PMID: WOS:000779205200013.

5. Aktas S, Unal S, Aksu M, Ozcan E, Ergenekon E, Turkyilmaz C, et al. Nasal HFOV with Binasal Cannula Appears Effective and Feasible in ELBW Newborns. Journal of Tropical Pediatrics. 2016;62(2):165-8. doi: 10.1093/tropej/fmv088. PubMed PMID: WOS:000374232000014.

6. Al Hashim AH, Al Reesi A, Al Lawati NM, Burad J, Al Khabori M, Chandwani J, et al. Comparison of Noninvasive Mechanical Ventilation With High-Flow Nasal Cannula, Face-Mask, and Helmet in Hypoxemic Respiratory Failure in Patients With COVID-19: A Randomized Controlled Trial. Critical Care Medicine. 2023;51(11):1515-26. doi: 10.1097/ccm.0000000000005963. PubMed PMID: WOS:001087381900015.

7. AlGhamdi Z, Alqahtani SY, AlDajani K, Alsaedi A, Al-Rubaish O, Alharbi A, et al. Pneumothorax in Critically Ill COVID-19 Patients: Prevalence, Analysis of Risk Factors and Clinical Outcomes. International Journal of General Medicine. 2022;15:8249-56. doi: 10.2147/ijgm.S387868. PubMed PMID: WOS:000890268500001.

8. Allison MG, Winters ME. Noninvasive Ventilation for the Emergency Physician. Emergency Medicine Clinics of North America. 2016;34(1):51-+. doi: 10.1016/j.emc.2015.08.004. PubMed PMID: WOS:000366963100007.

9. Alshahrani MS, Alshaqaq HM, Alhumaid J, Binammar AA, AlSalem KH, Alghamdi A, et al. High-Flow nasal cannula treatment in patients with COVID-19 acute hypoxemic respiratory failure: A prospective cohort study. Saudi Journal of Medicine & Medical Sciences. 2021;9(3):215-+. doi: 10.4103/sjmms.sjmms_316_21. PubMed PMID: WOS:000753899200002.

10. Al-Thaqafy MS, Alzahrani S, Alghamdi A, Alselemi S, Alshebani K, Bugis BA, et al. The efficiency of high-flow nasal cannula for adult patients with coronavirus disease 19 in Jeddah, Saudi Arabia. Annals of Thoracic Medicine. 2022;17(4):214-9. doi: 10.4103/atm.atm_156_22. PubMed PMID: WOS:000886545300006.

11. Ananthapadmanabhan S, Kudpaje A, Raju D, Smith M, Riffat F, Novakovic D, et al. Trans-nasal Humidified Rapid Insufflation Ventilatory Exchange (THRIVE) and its Utility in Otolaryngology, Head and Neck Surgery: A Literature Review. Indian Journal of Otolaryngology and Head & Neck Surgery. 2024. doi: 10.1007/s12070-023-04445-2. PubMed PMID: WOS:001136018700002.

12. Andino R, Vega G, Pacheco SK, Arevalillo N, Leal A, Fernández L, et al. High-flow nasal oxygen reduces endotracheal intubation: a randomized clinical trial. Therapeutic Advances in Respiratory Disease. 2020;14. doi: 10.1177/1753466620956459. PubMed PMID: WOS:000576095900001.

13. Apltekinoglu Mendil N, Temel S, Yüksel RC, Gündogan K, Eser B, Kaynar L, et al. The use of high-flow nasal oxygen vs. standard oxygen therapy in hematological malignancy patients with acute respiratory failure in hematology wards. Turkish Journal of Medical Sciences. 2021;51(4):1756-63. doi: 10.3906/sag-2007-228. PubMed PMID: WOS:000691544700022.

14. Arnold M, Wade C, Micetic B, Mody K. A Term Infant Presenting with COVID-19 Disease at Birth and a Croup-Like Cough. American Journal of Perinatology. 2024;41(01):110-3. doi: 10.1055/a-1877-6640. PubMed PMID: WOS:000849022100010.

15. Aroonpruksakul N, Sangsungnern P, Kiatchai T. Apneic oxygenation with low-flow oxygen cannula for rapid sequence induction and intubation in pediatric patients: a randomized-controlled trial. Translational Pediatrics. 2022;11(4):427-37. doi: 10.21037/tp-21-484. PubMed PMID: WOS:000767679500001.

16. Artaud-Macari E, Bubenheim M, Le Bouar G, Carpentier D, Grangé S, Boyer D, et al. High-flow oxygen therapy <i>versus</i> noninvasive ventilation: a randomised physiological crossover study of alveolar recruitment in acute respiratory failure. Erj Open Research. 2021;7(4). doi: 10.1183/23120541.00373-2021. PubMed PMID: WOS:000769759500032.

17. Ayanmanesh F, Abdat R, Jurine A, Azale M, Rousseaux G, Coulons S, et al. Transnasal humidified rapid-insufflation ventilatory exchange during rapid sequence induction in children. Anaesthesia Critical Care & Pain Medicine. 2021;40(2). doi: 10.1016/j.accpm.2021.100817. PubMed PMID: WOS:000706889000007.

18. Baillard C, Prat G, Jung B, Futier E, Lefrant JY, Vincent F, et al. Effect of preoxygenation using non-invasive ventilation before intubation on subsequent organ failures in hypoxaemic patients: a randomised clinical trial. British Journal of Anaesthesia. 2018;120(2):361-7. doi: 10.1016/j.bja.2017.11.067. PubMed PMID: WOS:000438189100019.

19. Bailly A, Ricard JD, Le Thuaut A, Helms J, Kamel T, Mercier E, et al. Compared Efficacy of Four Preoxygenation Methods for Intubation in the ICU: Retrospective Analysis of McGrath Mac Videolaryngoscope Versus Macintosh Laryngoscope (MACMAN) Trial Data. Critical Care Medicine. 2019;47(4):E340-E8. doi: 10.1097/ccm.0000000000003656. PubMed PMID: WOS:000469386100009.

20. Baloglu O, Flagg LK, Suleiman A, Gupta V, Fast JA, Wang L, et al. Association of Fluid Overload with Escalation of Respiratory Support and Endotracheal Intubation in Acute Bronchiolitis Patients. Journal of Pediatric Intensive Care. 2024;13(01):7-17. doi: 10.1055/s-0041-1735873. PubMed PMID: WOS:000695676200002.

21. Barbosa A, Mosier JM. Preoxygenation and apneic oxygenation in emergency airway management. Clinical and Experimental Emergency Medicine. 2024;11(2):136-44. doi: 10.15441/ceem.23.089. PubMed PMID: WOS:001262694600003.

22. Basile MC, Mauri T, Spinelli E, Dalla Corte F, Montanari G, Marongiu I, et al. Nasal high flow higher than 60 L/min in patients with acute hypoxemic respiratory failure: a physiological study. Critical Care. 2020;24(1). doi: 10.1186/s13054-020-03344-0. PubMed PMID: WOS:000595785100002.

23. Baudin F, Gagnon S, Crulli B, Proulx F, Jouvet P, Emeriaud G. Modalities and Complications Associated With the Use of High-Flow Nasal Cannula: Experience in a Pediatric ICU. Respiratory Care. 2016;61(10):1305-10. doi: 10.4187/respcare.04452. PubMed PMID: WOS:000385689800005.

24. Bauer PR, Gajic O, Nanchal R, Kashyap R, Martin-Loeches I, Sakr Y, et al. Association between timing of intubation and outcome in critically ill patients: A secondary analysis of the ICON audit. Journal of Critical Care. 2017;42:1-5. doi: 10.1016/j.jcrc.2017.06.010. PubMed PMID: WOS:000418520500002.

25. Bauer PR, Kumbamu A, Wilson ME, Pannu JK, Egginton JS, Kashyap R, et al. Timing of Intubation in Acute Respiratory Failure Associated With Sepsis: A Mixed Methods Study. Mayo Clinic Proceedings. 2017;92(10):1502-10. doi: 10.1016/j.mayocp.2017.07.001. PubMed PMID: WOS:000411959800010.

26. Benninger MS, Zhang ES, Chen BN, Tierney WS, Abdelmalak B, Bryson PC. Utility of Transnasal Humidified Rapid Insufflation Ventilatory Exchange for Microlaryngeal Surgery. Laryngoscope. 2021;131(3):587-91. doi: 10.1002/lary.28776. PubMed PMID: WOS:000540303200001.

27. Bergese SD, Bender SP, McSweeney TD, Fernandez S, Dzwonczyk R, Do KS. A comparative study of dexmedetomidine with midazolam and midazolam alone for sedation during elective awake fiberoptic intubation. Journal of Clinical Anesthesia. 2010;22(1):35-40. doi: 10.1016/j.jclinane.2009.02.016. PubMed PMID: WOS:000276783800007.

28. Berlin D, Singh I, Barjaktarevic I, Friedman O. A Technique for Bronchoscopic Intubation During High-Flow Nasal Cannula Oxygen Therapy. Journal of Intensive Care Medicine. 2016;31(3):213-5. doi: 10.1177/0885066615582020. PubMed PMID: WOS:000369076900008.

29. Besnier E, Hobeika S, Nseir S, Lambiotte F, Du Cheyron D, Sauneuf B, et al. High-flow nasal cannula therapy: clinical practice in intensive care units. Annals of Intensive Care. 2019;9(1). doi: 10.1186/s13613-019-0569-9. PubMed PMID: WOS:000484442900001.

30. Botan E, Uyar E, Öztürk Z, Sevketoglu E, Sari Y, Dursun O, et al. COVID-19 Transmission and Clinical Features in Pediatric Intensive Care Health Care Workers. Turkish Archives of Pediatrics. 2022;57(1):93-8. doi: 10.5152/TurkArchPediatr.2022.21205. PubMed PMID: WOS:000744060100015.

31. Boulton AJ, Mashru A, Lyon R. Oxygenation strategies prior to and during prehospital emergency anaesthesia in UK HEMS practice (PREOXY survey). Scandinavian Journal of Trauma Resuscitation & Emergency Medicine. 2020;28(1). doi: 10.1186/s13049-020-00794-x. PubMed PMID: WOS:000579131100001.

32. Bourn S, Milligan P, McNarry AF. Use of transnasal humidified rapid-insufflation ventilatory exchange (THRIVE) to facilitate the management of subglottic stenosis in pregnancy. International Journal of Obstetric Anesthesia. 2020;41:108-13. doi: 10.1016/j.ijoa.2019.07.004. PubMed PMID: WOS:000518724200015.

33. Brodsky MB, Mayfield EB, Gross RD. Clinical Decision Making in the ICU: Dysphagia Screening, Assessment, and Treatment. Seminars in Speech and Language. 2019;40(3):170-87. doi: 10.1055/s-0039-1688980. PubMed PMID: WOS:000474856200005.

34. Busico M, Laiz MM, Urrutia JG, Amado ME, Villalba D, Saavedra SN, et al. Strategies to achieve adherence to prone positioning in awake COVID-19 patients with high-flow nasal oxygen. A case series. Canadian Journal of Respiratory Therapy. 2022;58(1):151-4. doi: 10.29390/cjrt-2022-035. PubMed PMID: WOS:001120696700017.

35. Can FK, Anil AB, Anil M, Zengin N, Bal A, Bicilioglu Y, et al. Impact of High-flow Nasal Cannula Therapy in Quality Improvement and Clinical Outcomes in a Non-invasive Ventilation Device-free Pediatric Intensive Care Unit. Indian Pediatrics. 2017;54(10):835-40. doi: 10.1007/s13312-017-1145-8. PubMed PMID: WOS:000413698100007.

36. Caputo ND, Strayer RJ, Levitan R. Early Self-Proning in Awake, Non-intubated Patients in the Emergency Department: A Single ED's Experience During the COVID-19 Pandemic. Academic Emergency Medicine. 2020;27(5):375-8. doi: 10.1111/acem.13994. PubMed PMID: WOS:000531690800002.

37. Carrié C, Rieu B, Benard A, Trin K, Petit L, Massri A, et al. Early non-invasive ventilation and high-flow nasal oxygen therapy for preventing endotracheal intubation in hypoxemic blunt chest trauma patients: the OptiTHO randomized trial. Critical Care. 2023;27(1). doi: 10.1186/s13054-023-04429-2. PubMed PMID: WOS:000977329400001.

38. Carrillo-Alcaraz A, Guia M, Lopez-Gomez L, Bayoumy P, Alonso-Fernández N, Martínez-Quintana ME, et al. Analysis of combined non-invasive respiratory support in the first six waves of the COVID-19 pandemic. Outcome according to the first respiratory support. Trends in Anaesthesia and Critical Care. 2023;48. doi: 10.1016/j.tacc.2022.101208. PubMed PMID: WOS:000923052600001.

39. Carter MR, Khan AH, Salman T, Speicher R, Rotta AT, Shein SL. Emergency room endotracheal intubation in children with bronchiolitis: A cohort study using a multicenter database. Health Science Reports. 2020;3(3). doi: 10.1002/hsr2.169. PubMed PMID: WOS:000682998500004.

40. Casey JD, Janz DR, Russell DW, Vonderhaar DJ, Joffe AM, Dischert KM, et al. Bag-Mask Ventilation during Tracheal Intubation of Critically Ill Adults. New England Journal of Medicine. 2019;380(9):811-21. doi: 10.1056/NEJMoa1812405. PubMed PMID: WOS:000459899100006.

41. Castro S, Pedrero S, Ruiz LA, Serrano L, Zalacain R, Perez-Fernandez S, et al. High-flow nasal cannula oxygen therapy for the treatment of acute respiratory failure secondary to SARS-CoV-2 pneumonia out of ICU. Clinical Respiratory Journal. 2023;17(9):905-14. doi: 10.1111/crj.13679. PubMed PMID: WOS:001040072400001.

42. Celejewska-Wójcik N, Polok K, Górka K, Stachura T, Kania A, Nastalek P, et al. High-flow nasal oxygen therapy in the treatment of acute respiratory failure in severe COVID-19 pneumonia: a prospective observational study. Polish Archives of Internal Medicine-Polskie Archiwum Medycyny Wewnetrznej. 2021;131(7-8):658-65. doi: 10.20452/pamw.16015. PubMed PMID: WOS:000700887400001.

43. Cesar RG, Bispo BRP, Felix P, Modolo MCC, Souza AAF, Horigoshi NK, et al. High-Flow Nasal Cannula versus Continuous Positive Airway Pressure in Critical Bronchiolitis: A Randomized Controlled Pilot. Journal of Pediatric Intensive Care. 2020;09(04):248-55. doi: 10.1055/s-0040-1709656. PubMed PMID: WOS:000583627400003.

44. Chalkias A, Pavlopoulos F, Papageorgiou E, Tountas C, Anania A, Panteli M, et al. Development and Testing of a Novel Anaesthesia Induction/Ventilation Protocol for Patients With Cardiogenic Shock Complicating Acute Myocardial Infarction. Canadian Journal of Cardiology. 2018;34(8):1048-58. doi: 10.1016/j.cjca.2018.04.015. PubMed PMID: WOS:000439561200021.

45. Chandel A, Patolia S, Ahmad K, Aryal S, Brown AW, Sahjwani D, et al. Inhaled Nitric Oxide via High-Flow Nasal Cannula in Patients with Acute Respiratory Failure Related to COVID-19. Clinical Medicine Insights-Circulatory Respiratory and Pulmonary Medicine. 2021;15. doi: 10.1177/11795484211047065. PubMed PMID: WOS:000703767400001.

46. Chang HM, Fu BR, Yu HW, Chung FT, Seak CJ, Chen YC, et al. Effectiveness of a Protective Barrier for Aerosol Transmission Control during Oxygen Therapy with Different Devices. Aerosol and Air Quality Research. 2024;24(7). doi: 10.4209/aaqr.230285. PubMed PMID: WOS:001272192500002.

47. Chelly J, Coupry LM, Vong LV, Kamel T, Marzouk M, Terzi N, et al. Comparison of high-flow nasal therapy, noninvasive ventilation, and continuous positive airway pressure on outcomes in critically ill patients admitted for COVID-19 with acute respiratory failure. Minerva Anestesiologica. 2023;89(1):66-73. doi: 10.23736/s0375-9393.22.16918-x. PubMed PMID: WOS:000969802900009.

48. Choung HW, Choi YJ, Kang H, Lei UL. Four Different Reasons of Subcutaneous Emphysema With or Without Pneumomediastinum and Pneumothorax After Facial Bone Surgery. Journal of Craniofacial Surgery. 2022;33(6):E616-E20. doi: 10.1097/scs.0000000000008693. PubMed PMID: WOS:000847808200028.

49. Chua MT, Khan FA, Ng WM, Lu QS, Low MJW, Yau YW, et al. Pre- and Apnoeic high flow oxygenation for RApid sequence intubation in The Emergency department (Pre-AeRATE): study protocol for a multicentre, randomised controlled trial. Trials. 2019;20. doi: 10.1186/s13063-019-3305-8. PubMed PMID: WOS:000463643200003.

50. Chua MT, Ng WM, Lu QS, Low MJW, Punyadasa A, Cove ME, et al. Pre- and apnoeic high-flow oxygenation for rapid sequence intubation in the emergency department (the Pre-AeRATE trial): A multicentre randomised controlled trial. Annals Academy of Medicine Singapore. 2022;51(3):149-60. doi: 10.47102/annals-acadmedsg.2021407. PubMed PMID: WOS:000778647200005.

51. Ciril MF, Akarca M, Akoglu EU, Ozturk TC, Onur Ö. High-Flow Nasal Cannula versus Bag Valve Mask for Preoxygenation during Rapid Sequence Intubation in the Emergency Department: A Single-Center, Prospective, Randomized Controlled Trial. Prehospital and Disaster Medicine. 2024;39(1):45-51. doi: 10.1017/s1049023x23006684. PubMed PMID: WOS:001127845100001.

52. Cmielewski P, Farrow N, Devereux S, Parsons D, Donnelley M. Gene therapy for Cystic Fibrosis: Improved delivery techniques and conditioning with lysophosphatidylcholine enhance lentiviral gene transfer in mouse lung airways. Experimental Lung Research. 2017;43(9-10):426-33. doi: 10.1080/01902148.2017.1395931. PubMed PMID: WOS:000419570800010.

53. Conway A, Collins P, Chang K, Kamboj N, Filici AL, Lam P, et al. High flow nasal oxygen during procedural sedation for cardiac implantable electronic device procedures A randomised controlled trial. European Journal of Anaesthesiology. 2021;38(8):839-49. doi: 10.1097/eja.0000000000001458. PubMed PMID: WOS:000669935400006.

54. Corl KA, Dado C, Agarwal A, Azab N, Amass T, Marks SJ, et al. A modified Montpellier protocol for intubating intensive care unit patients is associated with an increase in first-pass intubation success and fewer complications. Journal of Critical Care. 2018;44:191-5. doi: 10.1016/j.jcrc.2017.11.014. PubMed PMID: WOS:000426565300036.

55. Corley A, Bull T, Spooner AJ, Barnett AG, Fraser JF. Direct extubation onto high-flow nasal cannulae post-cardiac surgery versus standard treatment in patients with a BMI ≥30: a randomised controlled trial. Intensive Care Medicine. 2015;41(5):887-94. doi: 10.1007/s00134-015-3765-6. PubMed PMID: WOS:000353841600014.

56. Cortegiani A, Longhini F, Carlucci A, Scala R, Groff P, Bruni A, et al. High-flow nasal therapy versus noninvasive ventilation in COPD patients with mild-to-moderate hypercapnic acute respiratory failure: study protocol for a noninferiority randomized clinical trial. Trials. 2019;20. doi: 10.1186/s13063-019-3514-1. PubMed PMID: WOS:000476844400003.

57. Costa WND, Miguel JP, Prado FD, Lula L, Amarante GAJ, Righetti RF, et al. Noninvasive ventilation and high-flow nasal cannula in patients with acute hypoxemic respiratory failure by covid-19: A retrospective study of the feasibility, safety and outcomes. Respiratory Physiology & Neurobiology. 2022;298. doi: 10.1016/j.resp.2022.103842. PubMed PMID: WOS:000767505000005.

58. Crewdson K, Heywoth A, Rehn M, Sadek S, Lockey D. Apnoeic oxygenation for emergency anaesthesia of pre-hospital trauma patients. Scandinavian Journal of Trauma Resuscitation & Emergency Medicine. 2021;29(1). doi: 10.1186/s13049-020-00817-7. PubMed PMID: WOS:000608248400001.

59. Crisafulli E, Sartori G, Vianello A, Maroccia A, Lepori E, Quici M, et al. Use of non-invasive respiratory supports in high-intensity internal medicine setting during the first two waves of the COVID-19 pandemic emergency in Italy: a multicenter, real-life experience. Internal and Emergency Medicine. 2023;18(6):1777-87. doi: 10.1007/s11739-023-03371-z. PubMed PMID: WOS:001032836000001.

60. Davis PG, Morley CJ, Owen LS. Non-invasive respiratory support of preterm neonates with respiratory distress: Continuous positive airway pressure and nasal intermittent positive pressure ventilation. Seminars in Fetal & Neonatal Medicine. 2009;14(1):14-20. doi: 10.1016/j.siny.2008.08.003. PubMed PMID: WOS:000262757600004.

61. De Jong A, Rolle A, Molinari N, Paugam-Burtz C, Constantin JM, Lefrant JY, et al. Cardiac Arrest and Mortality Related to Intubation Procedure in Critically Ill Adult Patients: A Multicenter Cohort Study. Critical Care Medicine. 2018;46(4):532-9. doi: 10.1097/ccm.0000000000002925. PubMed PMID: WOS:000427775500029.

62. de la Cuesta RMR, Guerra PD, Norniella CM, Izquierdo FB, García JG, Villanueva AM, et al. Boussignac continuous positive airway pressure device during inter-hospital transportation in infants aged less than three months. Anales Del Sistema Sanitario De Navarra. 2019;42(1):49-54. doi: 10.23938/assn.0587. PubMed PMID: WOS:000465568300006.

63. Delacroix E, Millet A, Pin I, Mortamet G. Use of bilevel positive pressure ventilation in patients with bronchiolitis. Pediatric Pulmonology. 2020;55(11):3134-8. doi: 10.1002/ppul.25033. PubMed PMID: WOS:000563326100001.

64. Delbove A, Foubert A, Mateos F, Guy T, Gousseff M. High flow nasal cannula oxygenation in COVID-19 related acute respiratory distress syndrome: a safe way to avoid endotracheal intubation? Therapeutic Advances in Respiratory Disease. 2021;15. doi: 10.1177/17534666211019555. PubMed PMID: WOS:000756003600001.

65. Dike CR, Rahhal R, Bishop WP. Is Carbon Dioxide Insufflation During Endoscopy in Children as Safe and as Effective as We Think? Journal of Pediatric Gastroenterology and Nutrition. 2020;71(2):211-5. doi: 10.1097/mpg.0000000000002724. PubMed PMID: WOS:000605965200026.

66. Ding L, Zhao Y, Li XY, Wang R, Li Y, Tang X, et al. Early diagnosis and appropriate respiratory support for <i>Mycoplasma pneumoniae</i> pneumonia associated acute respiratory distress syndrome in young and adult patients: a case series from two centers. Bmc Infectious Diseases. 2020;20(1). doi: 10.1186/s12879-020-05085-5. PubMed PMID: WOS:000537712900006.

67. Ding YY, Huang TF, Ge YL, Gao J, Zhang Y. Effect of trans-nasal humidified rapid insufflation ventilatory exchange on reflux and microaspiration in patients undergoing laparoscopic cholecystectomy during induction of general anesthesia: a randomized controlled trial. Frontiers in Medicine. 2023;10. doi: 10.3389/fmed.2023.1212646. PubMed PMID: WOS:001069850700001.

68. Disma N, Asai T, Cools E, Cronin A, Engelhardt T, Fiadjoe J, et al. Airway management in neonates and infants: European Society of Anaesthesiology and Intensive Care and British Journal of Anaesthesia joint guidelines. British Journal of Anaesthesia. 2024;132(1):124-44. doi: 10.1016/j.bja.2023.08.040. PubMed PMID: WOS:001152443600001.

69. Disma N, Asai T, Cools E, Cronin A, Engelhardt T, Fiadjoe J, et al. Airway management in neonates and infants. European Journal of Anaesthesiology. 2024;41(1):3-23. doi: 10.1097/eja.0000000000001928. PubMed PMID: WOS:001114643500011.

70. Doshi P, Whittle JS, Bublewicz M, Kearney J, Ashe T, Graham R, et al. High-Velocity Nasal Insufflation in the Treatment of Respiratory Failure: A Randomized Clinical Trial. Annals of Emergency Medicine. 2018;72(1):73-83. doi: 10.1016/j.annemergmed.2017.12.006. PubMed PMID: WOS:000438661000018.

71. Doyle AJ, Stolady D, Mariyaselvam M, Wijewardena G, Gent E, Blunt M, et al. Preoxygenation and apneic oxygenation using Transnasal Humidified Rapid-Insufflation Ventilatory Exchange for emergency intubation. Journal of Critical Care. 2016;36:8-12. doi: 10.1016/j.jcrc.2016.06.011. PubMed PMID: WOS:000393078100003.

72. Dyett JF, Moser MS, Tobin AE. Prospective observational study of emergency airway management in the critical care environment of a tertiary hospital in Melbourne. Anaesthesia and Intensive Care. 2015;43(5):577-86. doi: 10.1177/0310057x1504300505. PubMed PMID: WOS:000361270000005.

73. Elagamy AE, Taha SS, Elfawy DM. High flow nasal cannula versus non- invasive ventilation in prevention of intubation in immunocompromised patient with acute hypoxemic respiratory failure. Egyptian Journal of Anaesthesia. 2021;37(1):432-9. doi: 10.1080/11101849.2021.1978744. PubMed PMID: WOS:000695293100001.

74. Endlich Y, Beckmann LA, Choi SW, Culwick MD. A prospective six-month audit of airway incidents during anaesthesia in twelve tertiary level hospitals across Australia and New Zealand. Anaesthesia and Intensive Care. 2020;48(5):389-98. doi: 10.1177/0310057x20945325. PubMed PMID: WOS:000582496300008.

75. Esperatti M, Busico M, Fuentes NA, Gallardo A, Osatnik J, Vitali A, et al. Impact of exposure time in awake prone positioning on clinical outcomes of patients with COVID-19-related acute respiratory failure treated with high-flow nasal oxygen: a multicenter cohort study. Critical Care. 2022;26(1). doi: 10.1186/s13054-021-03881-2. PubMed PMID: WOS:000740240900001.

76. Ferrando C, Mellado-Artigas R, Gea A, Arruti E, Aldecoa C, Adalia R, et al. Awake prone positioning does not reduce the risk of intubation in COVID-19 treated with high-flow nasal oxygen therapy: a multicenter, adjusted cohort study. Critical Care. 2020;24(1). doi: 10.1186/s13054-020-03314-6. PubMed PMID: WOS:000578418700004.

77. Foley LJ, Urdaneta F, Berkow L, Aziz MF, Baker PA, Jagannathan N, et al. Difficult Airway Management in Adult Coronavirus Disease 2019 Patients: Statement by the Society of Airway Management. Anesthesia and Analgesia. 2021;133(4):876-90. doi: 10.1213/ane.0000000000005554. PubMed PMID: WOS:000693738300018.

78. Fong KM, Au SY, Ng GWY. Preoxygenation before intubation in adult patients with acute hypoxemic respiratory failure: a network meta-analysis of randomized trials. Critical Care. 2019;23(1). doi: 10.1186/s13054-019-2596-1. PubMed PMID: WOS:000486720200005.

79. Foran J, Moore CM, Chathasaigh CMN, Moore S, Purna JR, Curley A. Nasal high-flow therapy to Optimise Stability during Intubation: the NOSI pilot trial. Archives of Disease in Childhood-Fetal and Neonatal Edition. 2023;108(3):244-9. doi: 10.1136/archdischild-2022-324649. PubMed PMID: WOS:000877091700001.

80. Forsberg IM, Ullman J, Hoffman A, Eriksson LI, Lodenius Å, Fagerlund MJ. Lung volume changes in Apnoeic Oxygenation using Transnasal Humidified Rapid-Insufflation Ventilatory Exchange (THRIVE) compared to mechanical ventilation in adults undergoing laryngeal surgery. Acta Anaesthesiologica Scandinavica. 2020;64(10):1491-8. doi: 10.1111/aas.13686. PubMed PMID: WOS:000565556200001.

81. Franco C, Facciolongo N, Tonelli R, Dongilli R, Vianello A, Pisani L, et al. Feasibility and clinical impact of out-of-ICU noninvasive respiratory support in patients with COVID-19-related pneumonia. European Respiratory Journal. 2020;56(5). doi: 10.1183/13993003.02130-2020. PubMed PMID: WOS:000607520600023.

82. Frat JP, Ragot S, Coudroy R, Constantin JM, Girault C, Prat G, et al. Predictors of Intubation in Patients With Acute Hypoxemic Respiratory Failure Treated With a Noninvasive Oxygenation Strategy. Critical Care Medicine. 2018;46(2):208-15. doi: 10.1097/ccm.0000000000002818. PubMed PMID: WOS:000425974200037.

83. Frat JP, Ragot S, Girault C, Perbet S, Prat G, Boulain T, et al. Effect of non-invasive oxygenation strategies in immunocompromised patients with severe acute respiratory failure: a post-hoc analysis of a randomised trial. Lancet Respiratory Medicine. 2016;4(8):646-52. doi: 10.1016/s2213-2600(16)30093-5. PubMed PMID: WOS:000380836800028.

84. Frat JP, Ricard JD, Coudroy R, Robert R, Ragot S, Thille AW, et al. Preoxygenation with non-invasive ventilation versus high-flow nasal cannula oxygen therapy for intubation of patients with acute hypoxaemic respiratory failure in ICU: the prospective randomised controlled FLORALI-2 study protocol. Bmj Open. 2017;7(12). doi: 10.1136/bmjopen-2017-018611. PubMed PMID: WOS:000423826700156.

85. Frat JP, Ricard JD, Quenot JP, Pichon N, Demoule A, Forel JM, et al. Non-invasive ventilation versus high-flow nasal cannula oxygen therapy with apnoeic oxygenation for preoxygenation before intubation of patients with acute hypoxaemic respiratory failure: a randomised, multicentre, open-label trial. Lancet Respiratory Medicine. 2019;7(4):303-12. doi: 10.1016/s2213-2600(19)30048-7. PubMed PMID: WOS:000462492000017.

86. Frerk C, Mitchell VS, McNarry AF, Mendonca C, Bhagrath R, Patel A, et al. Difficult Airway Society 2015 guidelines for management of unanticipated difficult intubation in adults. British Journal of Anaesthesia. 2015;115(6):827-48. doi: 10.1093/bja/aev371. PubMed PMID: WOS:000366380200050.

87. Frérou A, Maamar A, Rafi S, Lhommet C, Phelouzat P, Pontis E, et al. Monitoring Transcutaneously Measured Partial Pressure of CO<sub>2</sub> During Intubation in Critically III Subjects. Respiratory Care. 2021;66(6):1004-15. doi: 10.4187/respcare.08009. PubMed PMID: WOS:000656534000015.

88. Furlong-Dillard JM, Nguyen A, Facciolo MD, Feygin YB, Napolitano N, Emeriaud G, et al. Associations With Severe Desaturation Events Among Children Receiving Noninvasive Respiratory Support at Time of Intubation. Respiratory Care. 2023;68(12):1646-56. doi: 10.4187/respcare.10765. PubMed PMID: WOS:001162438800006.

89. Gallardo A, Dévoli AP, Arévalo G, Saavedra SN, Moracci RS, Pratto RA, et al. High-Flow Nasal Cannula and Standard Oxygen in Acute Hypoxemic Respiratory Failure Due to COVID-19. Respiratory Care. 2022;67(12):1534-41. doi: 10.4187/respcare.10019. PubMed PMID: WOS:000892566400005.

90. Gandolfo C, Bonfiglio M, Spinetto G, Ferraioli G, Barlascini C, Nicolini A, et al. Pneumomediastinum associated with severe pneumonia related to COVID-19: diagnosis and management. Minerva Medica. 2021;112(6):779-85. doi: 10.23736/s0026-4806.21.07585-6. PubMed PMID: WOS:000756982700014.

91. Gedikloglu M, Gulen M, Satar S, Icen YK, Avci A, Yesiloglu O, et al. How to treat patients with acute respiratory failure? Conventional oxygen therapy versus high-flow nasal cannula in the emergency department. Hong Kong Journal of Emergency Medicine. 2022;29(2):84-93. doi: 10.1177/1024907919886245. PubMed PMID: WOS:000497095100001.

92. Geiseler J, Westhoff M. Weaning from invasive mechanical ventilation. Medizinische Klinik-Intensivmedizin Und Notfallmedizin. 2021;116(8):715-26. doi: 10.1007/s00063-021-00858-5. PubMed PMID: WOS:000701387400002.

93. George S, Gibbons K, Williams T, Humphreys S, Gelbart B, Le Marsney R, et al. Transnasal Humidified Rapid Insufflation Ventilatory Exchange in children requiring emergent intubation (Kids THRIVE): a statistical analysis plan for a randomised controlled trial. Trials. 2023;24(1). doi: 10.1186/s13063-023-07330-z. PubMed PMID: WOS:001000250200001.

94. Gesso AS, Regasa TL, Goshu EM, Woldemariam LG, Fekede MS. Comparative efficacy of apneic oxygenation with face mask versus face mask alone pre-oxygenation to Prevent desaturation during endotracheal intubation of elective Pediatric surgical patients at St. Peter hospital, Addis Ababa Ethiopia:A single center prospective cohort study. International Journal of Surgery Open. 2023;57. doi: 10.1016/j.ijso.2023.100654. PubMed PMID: WOS:001073923500001.

95. Gipson CL, Tobias JD. Flail chest in a neonate resulting from nonaccidental trauma. Southern Medical Journal. 2006;99(5):536-8. doi: 10.1097/01.smj.0000216471.54786.e5. PubMed PMID: WOS:000241315500022.

96. Girault C, Boyer D, Jolly G, Carpentier D, Béduneau G, Frat JP. Operating principles, physiological effects and practical issues of high-flow nasal oxygen therapy. Revue Des Maladies Respiratoires. 2022;39(5):455-68. doi: 10.1016/j.rmr.2022.03.012. PubMed PMID: WOS:000810979500008.

97. Giron F, Rao S, Tapaskar N. A multicomponent oxygen delivery strategy for COVID-19 patients in a step-down intensive care unit: A case series. Respiratory Medicine Case Reports. 2020;31. doi: 10.1016/j.rmcr.2020.101209. PubMed PMID: WOS:000600668000072.

98. Grant S, Khan F, Keijzers G, Shirran M, Marneros L. Ventilator-assisted preoxygenation: Protocol for combining non-invasive ventilation and apnoeic oxygenation using a portable ventilator. Emergency Medicine Australasia. 2016;28(1):67-72. doi: 10.1111/1742-6723.12524. PubMed PMID: WOS:000370266000010.

99. Grenness A, Connolly T, Russell N, Agar NJM, Nicholson R, Borschmann M. Evaluation of the safety of carbon dioxide laser and transnasal humidified rapid insufflation ventilatory exchange in laryngeal surgery. Australian Journal of Otolaryngology. 2024;7. doi: 10.21037/ajo-20-41. PubMed PMID: WOS:001237895300001.

100. Guha D, Dwivedi D, Paul D, Chakrabarti S, Talukdar J, Singh S. A questionnaire-based cross-sectional pilot survey on adherence to the recognized guidelines by the airway managers during intubation at the time of COVID-19 pandemic. Indian Anaesthetists Forum. 2021;22(1):40-6. doi: 10.4103/TheIAForum.TheIAForum_142_20. PubMed PMID: WOS:000621450200007.

101. Guitton C, Ehrmann S, Volteau C, Colin G, Maamar A, Jean-Michel V, et al. Nasal high-flow preoxygenation for endotracheal intubation in the critically ill patient: a randomized clinical trial. Intensive Care Medicine. 2019;45(4):447-58. doi: 10.1007/s00134-019-05529-w. PubMed PMID: WOS:000463211400004.

102. Gupta B, Kerai S, Kakkar K, Gupta L. Role of High-flow Nasal Oxygen Therapy in Cases with Pulmonary Hypertension in an Intensive Care Unit Setting. Indian Journal of Critical Care Medicine. 2019;23(10):458-61. doi: 10.5005/jp-journals-10071-23264. PubMed PMID: WOS:000496525900005.

103. Habra B, Janahi IA, Dauleh H, Chandra P, Veten A. A comparison between high-flow nasal cannula and noninvasive ventilation in the management of infants and young children with acute bronchiolitis in the PICU. Pediatric Pulmonology. 2020;55(2):455-61. doi: 10.1002/ppul.24553. PubMed PMID: WOS:000506566800001.

104. Hagan KB, Carlson R, Arnold B, Nguyen L, Lee J, Weston B, et al. Safety of the LMA®Gastro™ for Endoscopic Retrograde Cholangiopancreatography. Anesthesia and Analgesia. 2020;131(5):1566-72. doi: 10.1213/ane.0000000000005183. PubMed PMID: WOS:000577698500040.

105. Hajnour MS, Amlih HF, Shabr FFB. Efficacy of HFNO during airway management of a COVID pneumonia patient with super morbid obesity undergoing emergency laparotomy. Saudi Journal of Anaesthesia. 2022;16(3):368-70. doi: 10.4103/sja.sja_327_22. PubMed PMID: WOS:000891263000016.

106. Hamp T, Prager G, Baron-Stefaniak J, Müller J, Bichler C, Plöchl W. Duration of safe apnea in patients with morbid obesity during passive oxygenation using high-flow nasal insufflation versus regular flow nasal insufflation, a randomized trial. Surgery for Obesity and Related Diseases. 2021;17(2):347-55. doi: 10.1016/j.soard.2020.09.027. PubMed PMID: WOS:000631552000017.

107. Hanouz JL, Lhermitte D, Gérard JL, Fischer MO. Comparison of pre-oxygenation using spontaneous breathing through face mask and high-flow nasal oxygen <i>A randomised controlled crossover study in healthy volunteers</i>. European Journal of Anaesthesiology. 2019;36(5):335-41. doi: 10.1097/eja.0000000000000954. PubMed PMID: WOS:000480686500004.

108. Hao JJ, Liu JY, Pu L, Li CS, Zhang M, Tan JB, et al. High-Flow Nasal Cannula Oxygen Therapy versus Non-Invasive Ventilation in AIDS Patients with Acute Respiratory Failure: A Randomized Controlled Trial. Journal of Clinical Medicine. 2023;12(4). doi: 10.3390/jcm12041679. PubMed PMID: WOS:000944987300001.

109. Harada K, Kurosawa S, Hino Y, Yamamoto K, Sakaguchi M, Ikegawa S, et al. Clinical utility of high-flow nasal cannula oxygen therapy for acute respiratory failure in patients with hematological disease. Springerplus. 2016;5. doi: 10.1186/s40064-016-2161-1. PubMed PMID: WOS:000375703200010.

110. Hart JC, Goldstein LN. Analysis of the airway registry from an academic emergency department in South Africa. Samj South African Medical Journal. 2020;110(6):484-90. doi: 10.7196/SAMJ.2020.v110i6.14120. PubMed PMID: WOS:000541170200024.

111. He PY, Bao X, Jiang FF, Liu XB, Xu W, Yu DF, et al. Evaluating high-flow oxygen therapy after mechanical thrombectomy under general anesthesia in acute ischemic stroke: A retrospective single-center study. Clinical Neurology and Neurosurgery. 2024;243. doi: 10.1016/j.clineuro.2024.108359. PubMed PMID: WOS:001263661600001.

112. Hermez LA, Spence CJ, Payton MJ, Nouraei SAR, Patel A, Barnes TH. A physiological study to determine the mechanism of carbon dioxide clearance during apnoea when using transnasal humidified rapid insufflation ventilatory exchange (THRIVE). Anaesthesia. 2019;74(4):441-9. doi: 10.1111/anae.14541. PubMed PMID: WOS:000460677500006.

113. Hernandez-Rubio JC, Abad MSC, Leon AY, Higuera GD, Rodriguez LG, Torrejon CG, et al. Outcomes of an intermediate respiratory care unit in the COVID-19 pandemic. Plos One. 2020;15(12). doi: 10.1371/journal.pone.0243968. PubMed PMID: WOS:000600176400078.

114. Hickey AJ, Cummings MJ, Short B, Brodie D, Panzer O, Madahar P, et al. Approach to the Physiologically Challenging Endotracheal Intubation in the Intensive Care Unit. Respiratory Care. 2023;68(10):1438-48. doi: 10.4187/respcare.10821. PubMed PMID: WOS:001099095900015.

115. Higgs A, McGrath BA, Goddard C, Rangasami J, Suntharalingam G, Gale R, et al. Guidelines for the management of tracheal intubation in critically ill adults. British Journal of Anaesthesia. 2018;120(2):323-52. doi: 10.1016/j.bja.2017.10.021. PubMed PMID: WOS:000438189100017.

116. Huang APH, Tsai FF, Chen CC, Lee TS, Kuo LT. Feasibility of Nonintubated Anesthesia for Lumboperitoneal Shunt Implantation. Clinics and Practice. 2022;12(3):449-56. doi: 10.3390/clinpract12030049. PubMed PMID: WOS:000816183000001.

117. Huang L, Manley BJ, Arnolda GRB, Owen LS, Wright IMR, Foster JP, et al. Cost-Effectiveness of Nasal High Flow Versus CPAP for Newborn Infants in Special-Care Nurseries. Pediatrics. 2021;148(2). doi: 10.1542/peds.2020-020438. PubMed PMID: WOS:000685243200028.

118. Huang L, Roberts CT, Manley BJ, Owen LS, Davis PG, Dalziel KM. Cost-Effectiveness Analysis of Nasal Continuous Positive Airway Pressure Versus Nasal High Flow Therapy as Primary Support for Infants Born Preterm. Journal of Pediatrics. 2018;196:58-64. doi: 10.1016/j.jpeds.2017.12.072. PubMed PMID: WOS:000432452300012.

119. Huang SW, Wang ZH, Chan YW, Jiang T. Airway Management of an Infant With Giant Neck Macro-Cystic Hygroma Utilizing a High-Flow Nasal Cannula. Cureus Journal of Medical Science. 2023;15(10). doi: 10.7759/cureus.46865. PubMed PMID: WOS:001096942900009.

120. Huang Y, Zhao J, Hua XT, Luo KR, Shi Y, Lin ZL, et al. Guidelines for high-flow nasal cannula oxygen therapy in neonates (2022). Journal of Evidence Based Medicine. 2023;16(3):394-413. doi: 10.1111/jebm.12546. PubMed PMID: WOS:001059531100001.

121. Huh G, Min SH, Cho SD, Cho YJ, Kwon SK. Application and Efficiency of Transnasal Humidified Rapid-Insufflation Ventilatory Exchange in Laryngeal Microsurgery. Laryngoscope. 2022;132(5):1061-8. doi: 10.1002/lary.29848. PubMed PMID: WOS:000693662600001.

122. Ishimori S, Nagase S, Kanagawa A, Nakajiri T, Okita S, Oyazato Y, et al. Modified setting of negative pressure in children with mild respiratory disease. Pediatrics International. 2021;63(7):838-44. doi: 10.1111/ped.14560. PubMed PMID: WOS:000660560000001.

123. Iskandar R, Kaban RK, Djer MM. Heated, humidified high-flow nasal cannula <i>vs</i>. nasal CPAP in infants with moderate respiratory distress. Paediatrica Indonesiana. 2019;59(6):327-35. doi: 10.14238/pi59.6.2019.327-35. PubMed PMID: WOS:000503419700007.

124. Jaber S, Bellani G, Blanch L, Demoule A, Esteban A, Gattinoni L, et al. The intensive care medicine research agenda for airways, invasive and noninvasive mechanical ventilation. Intensive Care Medicine. 2017;43(9):1352-65. doi: 10.1007/s00134-017-4896-8. PubMed PMID: WOS:000408116700015.

125. Jaber S, De Jong A, Schaefer MS, Zhang JQ, Ma XW, Hao XR, et al. Preoxygenation with standard facemask combining apnoeic oxygenation using high flow nasal cannula versuss standard facemask alone in patients with and without obesity: the OPTIMASK international study. Annals of Intensive Care. 2023;13(1). doi: 10.1186/s13613-023-01124-x. PubMed PMID: WOS:000963903400002.

126. Jaber S, Molinari N, De Jong A. New method of preoxygenation for orotracheal intubation in patients with hypoxaemic acute respiratory failure in the intensive care unit, non-invasive ventilation combined with apnoeic oxygenation by high flow nasal oxygen: the randomised OPTINIV study protocol. Bmj Open. 2016;6(8). doi: 10.1136/bmjopen-2016-011298. PubMed PMID: WOS:000382336700072.

127. Jaber S, Monnin M, Girard M, Conseil M, Cisse M, Carr J, et al. Apnoeic oxygenation via high-flow nasal cannula oxygen combined with non-invasive ventilation preoxygenation for intubation in hypoxaemic patients in the intensive care unit: the single-centre, blinded, randomised controlled OPTINIV trial. Intensive Care Medicine. 2016;42(12):1877-87. doi: 10.1007/s00134-016-4588-9. PubMed PMID: WOS:000387846500007.

128. Janssen ML, Türk Y, Baart SJ, Hanselaar W, Aga Y, van der Steen-Dieperink M, et al. Safety and Outcome of High-Flow Nasal Oxygen Therapy Outside ICU Setting in Hypoxemic Patients With COVID-19. Critical Care Medicine. 2024;52(1):E31-43. doi: 10.1097/ccm.0000000000006068. PubMed PMID: WOS:001126476100001.

129. Jarou ZJ, Beiser DG, Sharp WW, Chacko R, Goode D, Rubin DS, et al. Emergency Department-initiated High-flow Nasal Cannula for COVID-19 Respiratory Distress. Western Journal of Emergency Medicine. 2021;22(4):979-87. doi: 10.5811/westjem.2021.3.50116. PubMed PMID: WOS:000681356800024.

130. Jenkins SA, Marshall CF. Awake intubation made easy and acceptable. Anaesthesia and Intensive Care. 2000;28(5):556-61. doi: 10.1177/0310057x0002800514. PubMed PMID: WOS:000165206000015.

131. Jiang YC, Chen J, Cen FL, Li X, Song Z, Peng M, et al. Importance of respiratory airway management as well as psychological and rehabilitative treatments to COVID-19 patients. American Journal of Emergency Medicine. 2020;38(8). doi: 10.1016/j.ajem.2020.04.055. PubMed PMID: WOS:000553316500029.

132. Jo JY, Yoon J, Jang H, Kim WJ, Ku SW, Choi SS. Comparison of preoxygenation with a high-flow nasal cannula and a simple face mask before intubation in Korean patients with head and neck cancer. Acute and Critical Care. 2024;39(1). doi: 10.4266/acc.2022.01543. PubMed PMID: WOS:001243820000005.

133. Johannes J, Berlin DA, Patel P, Schenck EJ, West FM, Saggar R, et al. A Technique of Awake Bronchoscopic Endotracheal Intubation for Respiratory Failure in Patients With Right Heart Failure and Pulmonary Hypertension. Critical Care Medicine. 2017;45(9):E980-E4. doi: 10.1097/ccm.0000000000002586. PubMed PMID: WOS:000407843600010.

134. Johnson KN, Mon RA, Gadepalli SK, Kunisaki SM. Short-term respiratory outcomes of neonates with symptomatic congenital lung malformations. Journal of Pediatric Surgery. 2019;54(9):1766-70. doi: 10.1016/j.jpedsurg.2019.01.056. PubMed PMID: WOS:000483426100007.

135. Kacmarek RM. Noninvasive Respiratory Support for Postextubation Respiratory Failure. Respiratory Care. 2019;64(6):658-76. doi: 10.4187/respcare.06671. PubMed PMID: WOS:000472898500006.

136. Kang HYJ, Zhao ZL, Tong ZH. Effect of High-Flow Nasal Cannula Oxygen Therapy in Immunocompromised Subjects With Acute Respiratory Failure. Respiratory Care. 2020;65(3):369-76. doi: 10.4187/respcare.07205. PubMed PMID: WOS:000516800000015.

137. Kang K, Wang JF, Du X, Li NA, Jin SG, Ji YY, et al. A safer and more practical tracheotomy in invasive mechanical ventilated patients with COVID-19: A quality improvement study. Frontiers in Surgery. 2022;9. doi: 10.3389/fsurg.2022.1018637. PubMed PMID: WOS:000885137800001.

138. Kang MG, Kim K, Ju S, Park HW, Lee SJ, Koh JS, et al. Clinical efficacy of high-flow oxygen therapy through nasal cannula in patients with acute heart failure. Journal of Thoracic Disease. 2019;11(2):410-7. doi: 10.21037/jtd.2019.01.51. PubMed PMID: WOS:000459708900020.

139. Karlupia D, Garg K, Jain R, Grewal A. Transnasal Humidified Rapid-Insufflation Ventilatory Exchange Versus Conventional Facemask Breathing for Preoxygenation During Rapid Sequence Induction. Cureus Journal of Medical Science. 2023;15(8). doi: 10.7759/cureus.43063. PubMed PMID: WOS:001048051300013.

140. Katayama Y, Takanishi H, Sato Y, Fujita S, Enomoto M. Effect of oral care in reducing the incidence of early-onset ventilator-associated pneumonia in preterm infants. Pediatric Pulmonology. 2021;56(8):2570-5. doi: 10.1002/ppul.25451. PubMed PMID: WOS:000649801600001.

141. Ke HH, Hsu PK, Tsou MY, Ting CK. Nonintubated video-assisted thoracic surgery with high-flow oxygen therapy shorten hospital stay. Journal of the Chinese Medical Association. 2020;83(10):943-9. doi: 10.1097/jcma.0000000000000408. PubMed PMID: WOS:000575618100013.

142. Kharat A, Ribeiro C, Er B, Fisser C, López-Padilla D, Chatzivasiloglou F, et al. ERS International Congress 2021: highlights from the Respiratory Intensive Care Assembly. Erj Open Research. 2022;8(2). doi: 10.1183/23120541.00016-2022. PubMed PMID: WOS:000805175900002.

143. Kilinç M, Yildiz M, Mentes O, Durmaz G. Prognosis and evaluation of patients with chronic obstructive pulmonary disease intubated in intensive care unit. Anaesthesia Pain & Intensive Care. 2022;26(4):474-9. doi: 10.35975/apic.v26i4.1950. PubMed PMID: WOS:000861049300009.

144. Kim JY, Bae J, Lee KH, Kang L, Kim KN, Jeong MA. High-flow nasal cannula application in an infant patient with laryngomalacia during general anesthesia A case report. Medicine. 2021;100(49). doi: 10.1097/md.0000000000028102. PubMed PMID: WOS:000728829100049.

145. Kim NY, Shin JS, Jeong OJ, Kim WY. Factors associated with unsuccessful high-flow nasal cannula therapy in patients presenting to the emergency department for acute hypoxemic respiratory failure. International Emergency Nursing. 2023;66. doi: 10.1016/j.ienj.2022.101236. PubMed PMID: WOS:000912247600001.

146. King AB, Alvis BD, Hester D, Taylor S, Higgins M. Randomized trial of a novel double lumen nasopharyngeal catheter versus traditional nasal cannula during total intravenous anesthesia for gastrointestinal procedures. Journal of Clinical Anesthesia. 2017;38:52-6. doi: 10.1016/j.jclinane.2017.01.025. PubMed PMID: WOS:000399260900021.

147. Kotwinski D, Paton L, Langford R. The role of high flow nasal oxygen therapy in anaesthesia. British Journal of Hospital Medicine. 2018;79(11):620-7. doi: 10.12968/hmed.2018.79.11.620. PubMed PMID: WOS:000449982300022.

148. Kucukdemirci-Kaya P, Kilic I, Kaya M, Kelebek-Girgin N. Role and limitations of high-flow nasal oxygen therapy in COVID-19 patients: An observational study. Nigerian Journal of Clinical Practice. 2022;25(7):1088-93. doi: 10.4103/njcp.njcp_1646_21. PubMed PMID: WOS:000884497900017.

149. Kwon H, Ha SW, Kim B, Chae B, Kim SM, Hong SI, et al. Respiratory rate-oxygenation (ROX) index for predicting high-flow nasal cannula failure in patients with and without COVID-19. American Journal of Emergency Medicine. 2024;75:53-8. doi: 10.1016/j.ajem.2023.09.036. PubMed PMID: WOS:001102623500001.

150. Lacarra B, Hayotte A, Naudin J, Maroni A, Geslain G, Poncelet G, et al. Air leak test in the Paediatric Intensive Care Unit (ALTIPICU): rationale and protocol for a prospective multicentre observational study. Bmj Open. 2024;14(4). doi: 10.1136/bmjopen-2023-081314. PubMed PMID: WOS:001251672400009.

151. Lal A, Mishra AK, Akhtar J, Nabzdyk C. Pneumothorax and pneumomediastinum in COVID-19 acute respiratory distress syndrome. Monaldi Archives for Chest Disease. 2021;91(2). doi: 10.4081/monaldi.2021.1608. PubMed PMID: WOS:000656044200019.

152. Langer C, Wittekindt C, Arens C, Käbisch S. Apnoeic oxygenation with high flow nasal oxygen for interventional surgery of the larynx and pharynx. European Archives of Oto-Rhino-Laryngology. 2024. doi: 10.1007/s00405-024-08726-6. PubMed PMID: WOS:001223799900001.

153. Langeron O, Bourgain JL, Francon D, Amour J, Baillard C, Bouroche G, et al. Difficult intubation and extubation in adult anaesthesia. Anaesthesia Critical Care & Pain Medicine. 2018;37(6):639-51. doi: 10.1016/j.accpm.2018.03.013. PubMed PMID: WOS:000453619900037.

154. Laorden D, Gholamian-Ovejero S, Terán-Tinedo JR, Lorente-González M, Cano-Sanz E, Ortega-Fraile MA, et al. Clinical Findings and Outcomes From Subjects With COVID-19 Pneumonia in an Intermediate Respiratory Care Unit. Respiratory Care. 2023;68(1):67-76. doi: 10.4187/respcare.10193. PubMed PMID: WOS:000916494700010.

155. Laviola M, Niklas C, Das A, Bates DG, Hardman JG. Effect of oxygen fraction on airway rescue: a computational modelling study. British Journal of Anaesthesia. 2020;125(1):E69-E74. doi: 10.1016/j.bja.2020.01.004. PubMed PMID: WOS:000544318800014.

156. Law JA, Duggan LV, Asselin M, Baker P, Crosby E, Downey A, et al. Canadian Airway Focus Group updated consensus-based recommendations for management of the difficult airway: part 2. Planning and implementing safe management of the patient with an anticipated difficult airway. Canadian Journal of Anesthesia-Journal Canadien D Anesthesie. 2021;68(9):1405-36. doi: 10.1007/s12630-021-02008-z. PubMed PMID: WOS:000659046100003.

157. Lee BA, Shin WJ, Jeong D, Choi JM, Gwak M, Song IK. Use of a High-Flow Nasal Cannula in a Child With a Functional Single Ventricle and Difficult Airway. Journal of Cardiothoracic and Vascular Anesthesia. 2021;35(7):2128-31. doi: 10.1053/j.jvca.2020.08.019. PubMed PMID: WOS:000655505900032.

158. Lee JH, Lim CM, Koh Y, Hong SB, Song JW, Huh JW. High-flow nasal cannula oxygen therapy in idiopathic pulmonary fibrosis patients with respiratory failure. Journal of Thoracic Disease. 2020;12(3):966-72. doi: 10.21037/jtd.2019.12.48. PubMed PMID: WOS:000521736500094.

159. Lentz S, Grossman A, Koyfman A, Long B. HIGH-RISK AIRWAY MANAGEMENT IN THE EMERGENCY DEPARTMENT. PART I: DISEASES AND APPROACHES. Journal of Emergency Medicine. 2020;59(1):84-95. doi: 10.1016/j.jemermed.2020.05.008. PubMed PMID: WOS:000573259100019.

160. Li J, Liu B, Zhou QH, Ni HD, Liu MJ, Deng K. Pre-oxygenation with high-flow oxygen through the nasopharyngeal airway compared to facemask on carbon dioxide clearance in emergency adults: a prospective randomized non-blinded clinical trial. European Journal of Trauma and Emergency Surgery. 2024;50(3):1051-61. doi: 10.1007/s00068-023-02418-2. PubMed PMID: WOS:001131843000001.

161. Li S, Hsieh TC, Rehder KJ, Nett S, Kamat P, Napolitano N, et al. Frequency of Desaturation and Association With Hemodynamic Adverse Events During Tracheal Intubations in PICUs. Pediatric Critical Care Medicine. 2018;19(1):E41-E50. doi: 10.1097/pcc.0000000000001384. PubMed PMID: WOS:000419769000006.

162. Li ZY, Pang M, Yu YL, Peng TF, Hu ZH, Niu RJ, et al. Effect of different ventilation modalities on the early prognosis of patients with sleep apnea after acute ischemic stroke---protocol for a prospective, open-label and randomised controlled trial. Bmc Neurology. 2023;23(1). doi: 10.1186/s12883-023-03117-6. PubMed PMID: WOS:001002402600001.

163. Li ZY, Pang M, Zhang JN, Mao L, Wang XR, Sun P. Effect of ventilation modalities on the early prognosis of patients with poststroke sleep apnea. Annals of Clinical and Translational Neurology. 2024;11(2):355-67. doi: 10.1002/acn3.51956. PubMed PMID: WOS:001108784400001.

164. Liao YY, Wu HY, Lam CF, Wang YM. Post-extubation use of high-flow nasal oxygenation induces upper airway leak and intrathoracic sepsis after successful Bentall procedure: A case report. Medicine. 2023;102(28). doi: 10.1097/md.0000000000034240. PubMed PMID: WOS:001029742100013.

165. Lightdale JR, Goldmann DA, Feldman HA, Newburg AR, DiNardo JA, Fox VL. Microstream capnography improves patient monitoring during moderate sedation: A randomized, controlled trial. Pediatrics. 2006;117(6):E1170-E8. doi: 10.1542/peds.2005-1709. PubMed PMID: WOS:000237979000097.

166. Lionello F, Lapia F, Molena B, Padoan A, Lococo S, Arcaro G, et al. The Safety of a High-Flow Nasal Cannula in Neuromuscular Disease Patients with Acute Respiratory Failure: A Retrospective Case-Series Study. Journal of Clinical Medicine. 2023;12(18). doi: 10.3390/jcm12186061. PubMed PMID: WOS:001073586100001.

167. Liu D, Jin TY, Li W, Chen L, Xing D. Effect of high-flow nasal cannula on patients? recovery after inhalation general anesthesia. Pakistan Journal of Medical Sciences. 2023;39(3):687-92. doi: 10.12669/pjms.39.3.6638. PubMed PMID: WOS:000989167300013.

168. Liu S, Walline JH, Zhu HD, Li Y, Wang CT, Liu JH. High-flow nasal cannula therapy with sequential noninvasive ventilation versus noninvasive ventilation alone as the initial ventilatory strategy in acute COPD exacerbations: study protocol for a randomized controlled trial. Trials. 2022;23(1). doi: 10.1186/s13063-022-06963-w. PubMed PMID: WOS:000920680000005.

169. Liu TT, Duan YC, Li YC, Hu YY, Su LL, Zhang AP. ChatGPT achieves comparable accuracy to specialist physicians in predicting the efficacy of high-flow oxygen therapy. Heliyon. 2024;10(11). doi: 10.1016/j.heliyon.2024.e31750. PubMed PMID: WOS:001246464100001.

170. Lodenius Å, Piehl J, Östlund A, Ullman J, Fagerlund MJ. Transnasal humidified rapid-insufflation ventilatory exchange (THRIVE) vs. facemask breathing pre-oxygenation for rapid sequence induction in adults: a prospective randomised non-blinded clinical trial. Anaesthesia. 2018;73(5):564-71. doi: 10.1111/anae.14215. PubMed PMID: WOS:000430117000007.

171. Long B, Lentz S, Koyfman A, Gottlieb M. Evaluation and management of the critically ill adult asthmatic in the emergency department setting. American Journal of Emergency Medicine. 2021;44:441-51. doi: 10.1016/j.ajem.2020.03.029. PubMed PMID: WOS:000659354100015.

172. Loudermilk EP, Hartmannsgruber M, Stoltzfus DP, Langevin PB. A prospective study of the safety of tracheal extubation using a pediatric airway exchange catheter for patients with a known difficult airway. Chest. 1997;111(6):1660-5. doi: 10.1378/chest.111.6.1660. PubMed PMID: WOS:A1997XC90200037.

173. Luján M, Gómez CC, Peñuelas O, Ferrando C, Heili-Frades SB, Perales JMC, et al. Multidisciplinary Consensus on the Management of Non-Invasive Respiratory Support in the COVID-19 Patient. Archivos De Bronconeumologia. 2024;60(5):285-95. doi: 10.1016/j.arbres.2024.02.017. PubMed PMID: WOS:001245021200001.

174. Lyons C, McElwain J, Coughlan MG, O'Gorman DA, Harte BH, Kinirons B, et al. Pre-oxygenation with facemask oxygen vs high-flow nasal oxygen vs high-flow nasal oxygen plus mouthpiece: a randomised controlled trial. Anaesthesia. 2022;77(1):40-5. doi: 10.1111/anae.15556. PubMed PMID: WOS:000685932900001.

175. Mackling T, Shah T, Dimas V, Guleserian K, Sharma M, Forbess J, et al. Management of Single-Ventricle Patients With Berlin Heart EXCOR Ventricular Assist Device: Single-Center Experience. Artificial Organs. 2012;36(6):555-9. doi: 10.1111/j.1525-1594.2011.01403.x. PubMed PMID: WOS:000304751000014.

176. Maddani SS, Deepa HC, Rao S, Chaudhuri S. A Multicenter Cross-sectional Questionnaire-based Study to Know the Practices and Strategies of Ventilatory Management of COVID-19 Patients among the Treating Physicians. Indian Journal of Critical Care Medicine. 2020;24(8):643-8. doi: 10.5005/jp-journals-10071-23516. PubMed PMID: WOS:000582185400011.

177. Maggiore SM, Jaber S, Grieco DL, Mancebo J, Zakynthinos S, Demoule A, et al. High-Flow Versus VenturiMask Oxygen Therapy to Prevent Reintubation in Hypoxemic Patients after Extubation A Multicenter Randomized Clinical Trial. American Journal of Respiratory and Critical Care Medicine. 2022;206(12):1452-62. doi: 10.1164/rccm.202201-0065OC. PubMed PMID: WOS:000901056900009.

178. Maia IS, Kawano-Dourado L, Zampieri FG, Damiani LP, Nakagawa RH, Gurgel RM, et al. High flow nasal catheter therapy versus non-invasive positive pressure ventilation in acute respiratory failure (RENOVATE trial): protocol and statistical analysis plan. Critical Care and Resuscitation. 2022;24(1):61-70. doi: 10.51893/2022.1.Oa8. PubMed PMID: WOS:000766657700010.

179. Matveevskii AS, Mahmoud M. New airway device for ventilation and monitoring in pediatric patients undergoing MRI study. Journal of Clinical Monitoring and Computing. 2012;26(1):17-20. doi: 10.1007/s10877-011-9326-9. PubMed PMID: WOS:000299724700003.

180. McCormack E, Aysenne A, Cardona JJ, Chaiyamoon A, Bui CJ, Dumont AS, et al. Effects of intubation technique on intracranial pressure: a cadaveric study. Neurosurgical Review. 2023;46(1). doi: 10.1007/s10143-023-01996-4. PubMed PMID: WOS:000969471400002.

181. McQuade D, Miller MR, Hayes-Bradley C. Addition of Nasal Cannula Can Either Impair or Enhance Preoxygenation With a Bag Valve Mask: A Randomized Crossover Design Study Comparing Oxygen Flow Rates. Anesthesia and Analgesia. 2018;126(4):1214-8. doi: 10.1213/ane.0000000000002341. PubMed PMID: WOS:000427977400020.

182. Mellado-Artigas R, Mujica LE, Ruiz ML, Ferreyro BL, Angriman F, Arruti E, et al. Predictors of failure with high-flow nasal oxygen therapy in COVID-19 patients with acute respiratory failure: a multicenter observational study. Journal of Intensive Care. 2021;9(1). doi: 10.1186/s40560-021-00538-8. PubMed PMID: WOS:000625938500001.

183. Menga LS, Delle Cese L, Bongiovanni F, Lombardi G, Michi T, Luciani F, et al. High Failure Rate of Noninvasive Oxygenation Strategies in Critically Ill Subjects With Acute Hypoxemic Respiratory Failure Due to COVID-19. Respiratory Care. 2021;66(5):705-14. doi: 10.4187/respcare.08622. PubMed PMID: WOS:000646202900002.

184. Merry AF, van Waart H, Allen SJ, Baker PA, Cumin D, Frampton CMA, et al. Ease and comfort of pre-oxygenation with high-flow nasal oxygen cannulae vs. facemask: a randomised controlled trial. Anaesthesia. 2022;77(12):1346-55. doi: 10.1111/anae.15853. PubMed PMID: WOS:000854613800001.

185. Messina A, Pradella A, Alicino V, Neganov M, De Mattei G, Coppalini G, et al. Critical Care Outreach Team During COVID-19: Ventilatory Support in the Ward and Outcomes. Respiratory Care. 2021;66(6):928-35. doi: 10.4187/respcare.08743. PubMed PMID: WOS:000656534000006.

186. Metkus TS, Miller PE, Stephens RS, Schulman SP, Eid SM. Incidence, Predictors, and Outcomes of Failure of Noninvasive Ventilation in Acute Heart Failure Hospitalization. Respiratory Care. 2020;65(10):1527-33. doi: 10.4187/respcare.07661. PubMed PMID: WOS:000574672900015.

187. Meunier J, Guitton C. The role of HFNC in pre-oxygenation prior to intubation and the practice of invasive procedures. Revue Des Maladies Respiratoires. 2023;40(1). doi: 10.1016/j.rmr.2022.11.002. PubMed PMID: WOS:000928810000001.

188. Miguel-Montanes R, Hajage D, Messika J, Bertrand F, Gaudry S, Rafat C, et al. Use of High-Flow Nasal Cannula Oxygen Therapy to Prevent Desaturation During Tracheal Intubation of Intensive Care Patients With Mild-to-Moderate Hypoxemia. Critical Care Medicine. 2015;43(3):574-83. doi: 10.1097/ccm.0000000000000743. PubMed PMID: WOS:000349963600022.

189. Mir F, Patel A, Iqbal R, Cecconi M, Nouraei SAR. A randomised controlled trial comparing transnasal humidified rapid insufflation ventilatory exchange (THRIVE) pre-oxygenation with facemask pre-oxygenation in patients undergoing rapid sequence induction of anaesthesia. Anaesthesia. 2017;72(4):439-43. doi: 10.1111/anae.13799. PubMed PMID: WOS:000398061000004.

190. Mittal BM, Sheehan KR, Goodnough CL, Turkmani-Bazzi S, Sheppard KO, Bushell E. Transnasal Humidified Rapid-Insufflation Ventilatory Exchange for Difficult Airway Management in Adults With Recessive Dystrophic Epidermolysis Bullosa: A Case Series. A & a Practice. 2022;16(11). doi: 10.1213/xaa.0000000000001630. PubMed PMID: WOS:000884104500003.

191. Mo HJ, Campbell MJ, Fertel BS, Lam SW, Wells EJ, Casserly E, et al. Ketamine Safety and Use in the Emergency Department for Pain and Agitation/Delirium: A Health System Experience. Western Journal of Emergency Medicine. 2020;21(2):272-81. doi: 10.5811/westjem.2019.10.43067. PubMed PMID: WOS:000517818500014.

192. Moffitt CA, Deakins K, Cheifetz I, Clayton JA, Slain KN, Shein SL. Use of Negative Pressure Ventilation in Pediatric Critical Care: Experience in 56 PICUs in the Virtual Pediatric Systems Database (2009-2019). Pediatric Critical Care Medicine. 2021;22(6):E363-E8. doi: 10.1097/pcc.0000000000002690. PubMed PMID: WOS:000657471100004.

193. Morley SL. Non-invasive ventilation in paediatric critical care. Paediatric Respiratory Reviews. 2016;20:24-31. doi: 10.1016/j.prrv.2016.03.001. PubMed PMID: WOS:000389116600005.

194. Morsy RS, Badawy MMS, Said RN, Ali AA, Abuelhamd WA. A Comparative Study between Postextubation of Preterm Neonates into High-Flow Nasal Cannula versus Nasal Continuous Positive Airway Pressure. Iranian Journal of Neonatology. 2021;12(1):12-9. doi: 10.22038/ijn.2020.43810.1753. PubMed PMID: WOS:000604496700003.

195. Mosier J, Reardon RF, DeVries PA, Stang JL, Nelsen A, Prekker ME, et al. TIME TO LOSS OF PREOXYGENATION IN EMERGENCY DEPARTMENT PATIENTS. Journal of Emergency Medicine. 2020;59(5):637-42. doi: 10.1016/j.jemermed.2020.06.064. PubMed PMID: WOS:000598590500012.

196. Natalini D, Grieco DL, Santantonio MT, Mincione L, Toni F, Anzellotti GM, et al. Physiological effects of high-flow oxygen in tracheostomized patients. Annals of Intensive Care. 2019;9(1). doi: 10.1186/s13613-019-0591-y. PubMed PMID: WOS:000489096500001.

197. Natt B, Mosier J. Airway Management in the Critically Ill Patient. Current Anesthesiology Reports. 2021;11(2):116-27. doi: 10.1007/s40140-021-00448-3. PubMed PMID: WOS:000641642300001.

198. Natt BS, Malo J, Hypes CD, Sakles JC, Mosier JM. Strategies to improve first attempt success at intubation in critically ill patients. British Journal of Anaesthesia. 2016;117:60-8. doi: 10.1093/bja/aew061. PubMed PMID: WOS:000383715900011.

199. Ng I, Krieser R, Mezzavia P, Lee K, Tseng C, Douglas NWR, et al. The use of Transnasal Humidified Rapid-Insufflation Ventilatory Exchange (THRIVE) for pre-oxygenation in neurosurgical patients: a randomised controlled trial. Anaesthesia and Intensive Care. 2018;46(4):360-7. doi: 10.1177/0310057x1804600403. PubMed PMID: WOS:000444221900003.

200. Ni YN, Luo J, Yu H, Liu D, Liang BM, Liang ZA. The effect of high-flow nasal cannula in reducing the mortality and the rate of endotracheal intubation when used before mechanical ventilation compared with conventional oxygen therapy and noninvasive positive pressure ventilation. A systematic review and meta-analysis. American Journal of Emergency Medicine. 2018;36(2):226-33. doi: 10.1016/j.ajem.2017.07.083. PubMed PMID: WOS:000425850900010.

201. Ni YN, Luo J, Yu H, Liu D, Ni Z, Cheng JL, et al. Can High-flow Nasal Cannula Reduce the Rate of Endotracheal Intubation in Adult Patients With Acute Respiratory Failure Compared With Conventional Oxygen Therapy and Noninvasive Positive Pressure Ventilation? A Systematic Review and Meta-analysis. Chest. 2017;151(4):764-75. doi: 10.1016/j.chest.2017.01.004. PubMed PMID: WOS:000398731500018.

202. Nong LB, Liang WB, Yu YH, Xi Y, Liu DD, Zhang J, et al. Noninvasive ventilation support during fiberoptic bronchoscopy-guided nasotracheal intubation effectively prevents severe hypoxemia. Journal of Critical Care. 2020;56:12-7. doi: 10.1016/j.jcrc.2019.10.017. PubMed PMID: WOS:000519555700003.

203. Nord A, Linner R, Milesi I, Zannin E, di Castri M, Bianco F, et al. A novel delivery system for supraglottic atomization allows increased lung deposition rates of pulmonary surfactant in newborn piglets. Pediatric Research. 2020;87(6):1019-24. doi: 10.1038/s41390-019-0696-x. PubMed PMID: WOS:000529759500013.

204. Oliver M, Caputo ND, West JR, Hackett R, Sakles JC. Emergency physician use of end-tidal oxygen monitoring for rapidsequence intubation. Journal of the American College of Emergency Physicians Open. 2020;1(5):706-13. doi: 10.1002/emp2.12260. PubMed PMID: WOS:000648695300002.

205. Ospina-Tascón GA, Calderón-Tapia LE, García AF, Zarama V, Gómez-Alvarez F, Alvarez-Saa T, et al. Effect of High-Flow Oxygen Therapy vs Conventional Oxygen Therapy on Invasive Mechanical Ventilation and Clinical Recovery in Patients With Severe COVID-19 A Randomized Clinical Trial. Jama-Journal of the American Medical Association. 2021;326(21):2161-71. doi: 10.1001/jama.2021.20714. PubMed PMID: WOS:000730077500019.

206. Ou XF, Hua YS, Liu J, Gong CS, Zhao WL. Effect of high-flow nasal cannula oxygen therapy in adults with acute hypoxemic respiratory failure: a meta-analysis of randomized controlled trials. Canadian Medical Association Journal. 2017;189(7):E260-E7. doi: 10.1503/cmaj.160570. PubMed PMID: WOS:000395527600002.

207. Overmann KM, Boyd SD, Zhang Y, Kerrey BT. Apneic oxygenation to prevent oxyhemoglobin desaturation during rapid sequence intubation in a pediatric emergency department. American Journal of Emergency Medicine. 2019;37(8):1416-21. doi: 10.1016/j.ajem.2018.10.030. PubMed PMID: WOS:000477927100004.

208. Park S, Kim SY, Kim MS, Park WK, Byon HJ, Kim HJ. Comparison of preoxygenation efficiency measured by the oxygen reserve index between high-flow nasal oxygenation and facemask ventilation: a randomised controlled trial. Bmc Anesthesiology. 2023;23(1). doi: 10.1186/s12871-023-02126-9. PubMed PMID: WOS:000985497500001.

209. Patel A, Nouraei SAR. Transnasal Humidified Rapid-Insufflation Ventilatory Exchange (THRIVE): a physiological method of increasing apnoea time in patients with difficult airways. Anaesthesia. 2015;70(3):323-9. doi: 10.1111/anae.12923. PubMed PMID: WOS:000350109000014.

210. Patel S, Wali A. Airway Management of the Obstetric Patient. Current Anesthesiology Reports. 2020;10(4):350-60. doi: 10.1007/s40140-020-00422-5. PubMed PMID: WOS:000642459800006.

211. Patwa A, Shah A, Garg R, Divatia JV, Kundra P, Doctor JR, et al. All India difficult airway association (AIDAA) consensus guidelines for airway management in the operating room during the COVID-19 pandemic. Indian Journal of Anaesthesia. 2020;64:S107-S15. doi: 10.4103/ija.IJA_498_20. PubMed PMID: WOS:000615984400005.

212. Perera A, Alkouri H, Fogg T, Vassiliadis J, Mackenzie J, Wimalasena Y. Apnoeic oxygenation was associated with decreased desaturation rates during rapid sequence intubation in multiple Australian and New Zealand emergency departments. Emergency Medicine Journal. 2021;38(2):118-24. doi: 10.1136/emermed-2019-208424. PubMed PMID: WOS:000614237700010.

213. Petrillo F, Valenzano L, Franco C, Calò G, Dentico D, Manzoni P, et al. Pulmonary Recruitment Strategy in Preterm Neonates&lt;29 Weeks of Gestational Age to Reduce the Need for Intubation in the Delivery Room. American Journal of Perinatology. 2019;36:S115-S9. doi: 10.1055/s-0039-1692134. PubMed PMID: WOS:000482311200023.

214. Pillai A, Chikhani M, Hardman JG. Apnoeic oxygenation in pregnancy: a modelling investigation. Anaesthesia. 2016;71(9):1077-80. doi: 10.1111/anae.13563. PubMed PMID: WOS:000382949200012.

215. Pirat A, Candan S, Ünlükaplan A, Kömürcü Ö, Kuslu S, Arslan G. Percutaneous Dilational Tracheotomy for Airway Management in a Newborn With Pierre-Robin Syndrome and a Glossopharyngeal Web. Respiratory Care. 2012;57(4):627-30. doi: 10.4187/respcare.01404. PubMed PMID: WOS:000302928500015.

216. Poopipatpab S, Nuchpramool P, Phairatwet P, Lertwattanachai T, Trongtrakul K. The use of respiratory rate-oxygenation index to predict failure of high-flow nasal cannula in patients with coronavirus disease 2019-associated acute respiratory distress syndrome: A retrospective study. Plos One. 2023;18(6). doi: 10.1371/journal.pone.0287432. PubMed PMID: WOS:001017401300030.

217. Powell EK, Hinckley WR, Stolz U, Golden AJ, Ventura A, McMullan JT. PREDICTORS OF DEFINITIVE AIRWAY SANS HYPOXIA/HYPOTENSION ON FIRST ATTEMPT (DASH-1A) SUCCESS IN TRAUMATICALLY INJURED PATIENTS UNDERGOING PREHOSPITAL INTUBATION. Prehospital Emergency Care. 2020;24(4):470-7. doi: 10.1080/10903127.2019.1670299. PubMed PMID: WOS:000554682800001.

218. Qiu J, Xie M, Chen J, Chen B, Chen YJ, Zhu XW, et al. Tracheal Extubation Under Deep Anesthesia Using Transnasal Humidified Rapid Insufflation Ventilatory Exchange vs. Awake Extubation: An Open-Labeled Randomized Controlled Trial. Frontiers in Medicine. 2022;9. doi: 10.3389/fmed.2022.810366. PubMed PMID: WOS:000772196900001.

219. Quintard H, l'Her E, Pottecher J, Adnet F, Constantin JM, De Jong A, et al. Intubation and extubation of the ICU patient. Anaesthesia Critical Care & Pain Medicine. 2017;36(5):327-41. doi: 10.1016/j.accpm.2017.09.001. PubMed PMID: WOS:000416953200015.

220. Rajajee V, Riggs B, Seder DB. Emergency Neurological Life Support: Airway, Ventilation, and Sedation. Neurocritical Care. 2017;27:S4-S28. doi: 10.1007/s12028-017-0451-2. PubMed PMID: WOS:000424094100002.

221. Rao SV, Udhayachandar R, Rao VB, Raju NA, Nesaraj JJJ, Kandasamy S, et al. Voluntary Prone Position for Acute Hypoxemic Respiratory Failure in Unintubated Patients. Indian Journal of Critical Care Medicine. 2020;24(7):557-62. doi: 10.5005/jp-journals-10071-23495. PubMed PMID: WOS:000561881700015.

222. Rattanajiajaroen P, Kongpolprom N. Effects of high flow nasal cannula on the coordination between swallowing and breathing in postextubation patients, a randomized crossover study. Critical Care. 2021;25(1). doi: 10.1186/s13054-021-03786-0. PubMed PMID: WOS:000708910700001.

223. Ricard JD. Hazards of intubation in the ICU: role of nasal high flow oxygen therapy for preoxygenation and apneic oxygenation to prevent desaturation. Minerva Anestesiologica. 2016;82(10):1098-106. PubMed PMID: WOS:000386892300013.

224. Riedel T, Bürgi F, Greif R, Kaiser H, Riva T, Theiler L, et al. Changes in lung volume estimated by electrical impedance tomography during apnea and high-flow nasal oxygenation: A single-center randomized controlled trial. Plos One. 2022;17(9). doi: 10.1371/journal.pone.0273120. PubMed PMID: WOS:000945723600001.

225. Rigal T, Baudouin R, Circiu M, Couineau F, Lechien J, Crevier-Buchman L, et al. Laryngeal microsurgery under Transnasal Humidified Rapid Insufflation Ventilatory Exchange. Oto Open. 2024;8(2). doi: 10.1002/oto2.125. PubMed PMID: WOS:001244164000001.

226. Riva T, Greif R, Kaiser H, Riedel T, Huber M, Theiler L, et al. Carbon Dioxide Changes during High-flow Nasal Oxygenation in Apneic Patients: A Single-center Randomized Controlled Noninferiority Trial. Anesthesiology. 2022;136(1):82-92. doi: 10.1097/aln.0000000000004025. PubMed PMID: WOS:000728960200012.

227. Riva T, Meyer J, Theiler L, Obrist D, Bütikofer L, Greif R, et al. Measurement of airway pressure during high-flow nasal therapy in apnoeic oxygenation: a randomised controlled crossover trial*. Anaesthesia. 2021;76(1):27-35. doi: 10.1111/anae.15224. PubMed PMID: WOS:000557678400001.

228. Riva T, Préel N, Theiler L, Greif R, Bütikofer L, Ulmer F, et al. Evaluating the ventilatory effect of transnasal humidified rapid insufflation ventilatory exchange in apnoeic small children with two different oxygen flow rates: a randomised controlled trial*. Anaesthesia. 2021;76(7):924-32. doi: 10.1111/anae.15335. PubMed PMID: WOS:000600779100001.

229. Rochwerg B, Einav S, Chaudhuri D, Mancebo J, Mauri T, Helviz Y, et al. The role for high flow nasal cannula as a respiratory support strategy in adults: a clinical practice guideline. Intensive Care Medicine. 2020;46(12):2226-37. doi: 10.1007/s00134-020-06312-y. PubMed PMID: WOS:000590207500001.

230. Rodriguez M, Ragot S, Coudroy R, Quenot JP, Vignon P, Forel JM, et al. Noninvasive ventilation vs. high-flow nasal cannula oxygen for preoxygenation before intubation in patients with obesity: a post hoc analysis of a randomized controlled trial. Annals of Intensive Care. 2021;11(1). doi: 10.1186/s13613-021-00892-8. PubMed PMID: WOS:000680395900002.

231. Rubulotta F, Torra LB, Naidoo KD, Aboumarie HS, Mathivha LR, Asiri AY, et al. Mechanical Ventilation, Past, Present, and Future. Anesthesia and Analgesia. 2024;138(2):308-25. doi: 10.1213/ane.0000000000006701. PubMed PMID: WOS:001143125000028.

232. Rutt AL, Torp KD, Zimmermann T, Warner P, Hofer R, Charnin JE, et al. Apneic Technique in Laryngotracheal Surgery. Cureus Journal of Medical Science. 2022;14(1). doi: 10.7759/cureus.21584. PubMed PMID: WOS:000759221800018.

233. Saillard C, Lambert J, Tramier M, Chow-Chine L, Bisbal M, Servan L, et al. High-flow nasal cannula failure in critically ill cancer patients with acute respiratory failure: Moving from avoiding intubation to avoiding delayed intubation. Plos One. 2022;17(6). doi: 10.1371/journal.pone.0270138. PubMed PMID: WOS:000892027900059.

234. Sakles JC, Mosier JM, Patanwala AE, Arcaris B, Dicken JM. First Pass Success Without Hypoxemia Is Increased With the Use of Apneic Oxygenation During Rapid Sequence Intubation in the Emergency Department. Academic Emergency Medicine. 2016;23(6):703-10. doi: 10.1111/acem.12931. PubMed PMID: WOS:000383374900007.

235. Saksitthichok B, Petnak T, So-ngem A, Boonsamgsuk V. A prospective randomized comparative study of high-flow nasal cannula oxygen and non-invasive ventilation in hypoxemic patients undergoing diagnostic flexible bronchoscopy. Journal of Thoracic Disease. 2019;11(5):1929-39. doi: 10.21037/jtd.2019.05.02. PubMed PMID: WOS:000469979100027.

236. Sbaih N, Hawthorne K, Lutes J, Cavallazzi R. Nutrition Therapy in Non-intubated Patients with Acute Respiratory Failure. Current Nutrition Reports. 2021;10(4):307-16. doi: 10.1007/s13668-021-00367-z. PubMed PMID: WOS:000691638800001.

237. Schlueter D, Kovaleski C, Walter V, Thomas NJ, Krawiec C. Impact of Body Mass Index and Initial Respiratory Support on Pediatric Subjects in Acute Respiratory Failure. Respiratory Care. 2021;66(9):1425-32. doi: 10.4187/respcare.08735. PubMed PMID: WOS:000686562000008.

238. Schwartz BC, Jayaraman D, Yang SS, Wong EG, Lipes J, Dial S. High-flow nasal oxygen as first-line therapy for COVID-19-associated hypoxemic respiratory failure: a single-centre historical cohort study. Canadian Journal of Anesthesia-Journal Canadien D Anesthesie. 2022;69(5):582-90. doi: 10.1007/s12630-022-02218-z. PubMed PMID: WOS:000760697000001.

239. Scoccimarro A, West JR, Kanter M, Caputo ND. Waveform capnography: an alternative to physician gestalt in determining optimal intubating conditions after administration of paralytic agents. Emergency Medicine Journal. 2018;35(1):62-4. doi: 10.1136/emermed-2017-206922. PubMed PMID: WOS:000418226700012.

240. Sekar L, Sehgal IS, Kajal K, Kataria S, Premkumar M, Singla K, et al. Factors Associated With Non-invasive Oxygen Therapy Failure in COVID-19 Pneumonia: A Single Center, Retrospective Study in a Tertiary Hospital in North India. Cureus Journal of Medical Science. 2022;14(9). doi: 10.7759/cureus.29721. PubMed PMID: WOS:000886529200030.

241. Semler MW, Janz DR, Lentz RJ, Matthews DT, Norman BC, Assad TR, et al. Randomized Trial of Apneic Oxygenation during Endotracheal Intubation of the Critically III. American Journal of Respiratory and Critical Care Medicine. 2016;193(3):273-80. doi: 10.1164/rccm.201507-1294OC. PubMed PMID: WOS:000369355600012.

242. Semler MW, Janz DR, Russell DW, Casey JD, Lentz RJ, Zouk AN, et al. A Multicenter, Randomized Trial of Ramped Position vs Sniffing Position During Endotracheal Intubation of Critically Ill Adults. Chest. 2017;152(4):712-22. doi: 10.1016/j.chest.2017.03.061. PubMed PMID: WOS:000412429500014.

243. Sener K, Calis M, Köseoglu Z, Sari S, Polat M, Üzücek DM, et al. Comparison of high-flow oxygen treatment and standard oxygen treatment in patients with hypertensive pulmonary edema. Anatolian Journal of Cardiology. 2020;24(4):260-6. doi: 10.14744/AnatolJCardiol.2020.50680. PubMed PMID: WOS:000577198700010.

244. Shekar K, Varkey S, Cornmell G, Parsons L, Tol M, Siuba M, et al. Feasibility of non-invasive nitric oxide gas inhalation to prevent endotracheal intubation in patients with acute hypoxemic respiratory failure: A single-centre experience. Nitric Oxide-Biology and Chemistry. 2021;116:35-7. doi: 10.1016/j.niox.2021.08.005. PubMed PMID: WOS:000701672400005.

245. Shima T, Kashiwagi H, Ino H, Tanaka S, Fukuda M, Kobata H. Acute respiratory distress syndrome due to inhalation of acryloyl chloride. Acute Medicine & Surgery. 2022;9(1). doi: 10.1002/ams2.724. PubMed PMID: WOS:000743692500001.

246. Shippam W, Preston R, Douglas J, Taylor J, Albert A, Chau A. High-flow nasal oxygen vs. standard flow-rate facemask pre-oxygenation in pregnant patients: a randomised physiological study. Anaesthesia. 2019;74(4):450-6. doi: 10.1111/anae.14567. PubMed PMID: WOS:000460677500007.

247. Shoukri AM. High flow nasal cannula oxygen and non-invasive mechanical ventilation in management of COVID-19 patients with acute respiratory failure: a retrospective observational study. Egyptian Journal of Bronchology. 2021;15(1). doi: 10.1186/s43168-021-00063-0. PubMed PMID: WOS:000636317600017.

248. Simioli F, Annunziata A, Coppola A, Imitazione P, Mirizzi AI, Marotta A, et al. The role of dexmedetomidine in ARDS: an approach to non-intensive care sedation. Frontiers in Medicine. 2023;10. doi: 10.3389/fmed.2023.1224242. PubMed PMID: WOS:001068154300001.

249. Simon M, Wachs C, Braune S, de Heer G, Frings D, Kluge S. High-Flow Nasal Cannula Versus Bag-Valve-Mask for Preoxygenation Before Intubation in Subjects With Hypoxemic Respiratory Failure. Respiratory Care. 2016;61(9):1160-7. doi: 10.4187/respcare.04413. PubMed PMID: WOS:000382382400005.

250. Singh S, Jha RK, Chandi DH. Airway Management of Covid 19 and Lowcost Ventilators-Aboonor Bane. Bioscience Biotechnology Research Communications. 2021;14(6):267-70. doi: 10.21786/bbrc/14.6.56. PubMed PMID: WOS:000697827700056.

251. Sivasankar C, Schlichter RA, Baranov D, Kofke WA. Awake Craniotomy: A New Airway Approach. Anesthesia and Analgesia. 2016;122(2):509-11. doi: 10.1213/ane.0000000000001072. PubMed PMID: WOS:000368646600005.

252. Sjöblom A, Hedberg M, Gille A, Guerra A, Aanesen V, Forsberg IM, et al. Pre-oxygenation using high-flow nasal oxygen versus tight facemask in trauma patients undergoing emergency anaesthesia. Acta Anaesthesiologica Scandinavica. 2024;68(4):447-56. doi: 10.1111/aas.14368. PubMed PMID: WOS:001130305800001.

253. Somri M, Gaitini L, Yanovski B, Tome R, Resnikov I, Karsh K, et al. Flexible Upper Videoendoscopy Through a Modified Endoscopy Mask in Infants and Young Children. Journal of Pediatric Gastroenterology and Nutrition. 2009;49(2):191-5. doi: 10.1097/MPG.0b013e31818de362. PubMed PMID: WOS:000268519700007.

254. Soneru CN, Hurt HF, Petersen TR, Davis DD, Braude DA, Falcon RJ. Apneic nasal oxygenation and safe apnea time during pediatric intubations by learners. Pediatric Anesthesia. 2019;29(6):628-34. doi: 10.1111/pan.13645. PubMed PMID: WOS:000474489100010.

255. Sood BG, Cortez J, Kolli M, Sharma A, Delaney-Black V, Chen XG. Aerosolized surfactant in neonatal respiratory distress study. Early Human Development. 2019;134:19-25. doi: 10.1016/j.earlhumdev.2019.05.005. PubMed PMID: WOS:000474309200006.

256. Tam K, Jeffery C, Sung CK. Surgical Management of Supraglottic Stenosis Using Intubationless Optiflow. Annals of Otology Rhinology and Laryngology. 2017;126(9):669-72. doi: 10.1177/0003489417720220. PubMed PMID: WOS:000407822800006.

257. Tan DY, Wang BX, Cao P, Wang YY, Sun JY, Geng P, et al. High flow nasal cannula oxygen therapy versus non-invasive ventilation for acute exacerbations of chronic obstructive pulmonary disease with acute-moderate hypercapnic respiratory failure: a randomized controlled non-inferiority trial. Critical Care. 2024;28(1). doi: 10.1186/s13054-024-05040-9. PubMed PMID: WOS:001272767000004.

258. Tan PCF, Millay OJ, Leeton L, Dennis AT. High-flow humidified nasal preoxygenation in pregnant women: a prospective observational study. British Journal of Anaesthesia. 2019;122(1):86-91. doi: 10.1016/j.bja.2018.08.015. PubMed PMID: WOS:000453927600025.

259. Tauzin M, Durrmeyer X. Managing neonatal pain in the era of non-invasive respiratory support. Seminars in Fetal & Neonatal Medicine. 2019;24(4). doi: 10.1016/j.siny.2019.04.004. PubMed PMID: WOS:000485212200009.

260. Teran-Tinedo JR, Gonzalez-Rubio J, Najera A, Lorente-Gonzalez M, Cano-Sanz E, de la Calle-Gil I, et al. Effect of the Early Combination of Continuous Positive Airway Pressure and High-Flow Nasal Cannula on Mortality and Intubation Rates in Patients With COVID-19 and Acute Respiratory Distress Syndrome. The DUOCOVID Study. Archivos De Bronconeumologia. 2023;59(5):288-94. doi: 10.1016/j.arbres.2023.01.009. PubMed PMID: WOS:001026322800001.

261. Thille AW, Marie D, Reynaud F, Barrau S, Beuvon C, Bironneau V, et al. Sleep Assessment in Critically Ill Patients With Acute Hypoxemic Respiratory Failure. Respiratory Care. 2023;68(10):1417-25. doi: 10.4187/respcare.10844. PubMed PMID: WOS:001099095900012.

262. Thota B, Samantaray A, Vengamma B, Mangu HR, Alladi M, Kalawat U. A randomised controlled trial of high-flow nasal oxygen versus non-rebreathing oxygen face mask therapy in acute hypoxaemic respiratory failure. Indian Journal of Anaesthesia. 2022;66(9):644-50. doi: 10.4103/ija.ija_507_22. PubMed PMID: WOS:001183267100005.

263. Tohda G, Higashi S, Wakahara S, Morikawa M, Sakumoto H, Kane T. Propofol sedation during endoscopic procedures: Safe and effective administration by registered nurses supervised by endoscopists. Endoscopy. 2006;38(4):360-7. doi: 10.1055/s-2005-921192. PubMed PMID: WOS:000237309100005.

264. Tonelli R, Cortegiani A, Marchioni A, Fantini R, Tabbì L, Castaniere I, et al. Nasal pressure swings as the measure of inspiratory effort in spontaneously breathing patients with de novo acute respiratory failure. Critical Care. 2022;26(1). doi: 10.1186/s13054-022-03938-w. PubMed PMID: WOS:000772812900001.

265. Tran A, Thiruvenkatarajan V, Wahba M, Currie J, Rajbhoj A, van Wijk R, et al. LMA® Gastro™ Airway for endoscopic retrograde cholangiopancreatography: a retrospective observational analysis. Bmc Anesthesiology. 2020;20(1). doi: 10.1186/s12871-020-01019-5. PubMed PMID: WOS:000536130900001.

266. Tremey B, Squara P, De Labarre H, Ma S, Fischler M, Lawkoune JD, et al. Hands-free induction of general anesthesia: a randomised pilot study comparing usual care and high-flow nasal oxygen. Minerva Anestesiologica. 2020;86(11):1135-42. doi: 10.23736/s0375-9393.20.14456-0. PubMed PMID: WOS:000593105900006.

267. Tsao M, Wuenstel A, Zider B, Hajduk J, Jagannathan N. Current Concepts in the Management of the Difficult Pediatric Airway. Current Anesthesiology Reports. 2019;9(2):123-34. doi: 10.1007/s40140-019-00319-y. PubMed PMID: WOS:000642450200006.

268. Ucar EY, Araz Ö, Kerget B, Akgun M, Saglam L. Comparison of high-flow and conventional nasal cannula oxygen in patients undergoing endobronchial ultrasonography. Internal Medicine Journal. 2021;51(11):1935-9. doi: 10.1111/imj.15001. PubMed PMID: WOS:000710111200001.

269. Umobong EU, Mayo PH. Critical Care Airway Management. Critical Care Clinics. 2018;34(3):313-+. doi: 10.1016/j.ccc.2018.03.006. PubMed PMID: WOS:000438835400002.

270. Urbina T, Elabbadi A, Gabarre P, Bige N, Turpin M, Bonny V, et al. Endotracheal intubation rate is lower with additional face-mask noninvasive ventilation for critically-ill SARS-CoV-2 patients requiring high-flow nasal oxygen: a retrospective bicentric cohort with propensity score analysis. Minerva Anestesiologica. 2022;88(7-8):580-7. doi: 10.23736/s0375-9393.22.16094-3. PubMed PMID: WOS:000839024400009.

271. Valencia CF, Lucero OD, Castro OC, Sanko AA, Olejua PA. Comparison of ROX and HACOR scales to predict high-flow nasal cannula failure in patients with SARS-CoV-2 pneumonia. Scientific Reports. 2021;11(1). doi: 10.1038/s41598-021-02078-5. PubMed PMID: WOS:000720679400024.

272. Vaughan EM, Seitz KP, Janz DR, Russell DW, Dargin J, Vonderhaar DJ, et al. Bag-Mask Ventilation Versus Apneic Oxygenation During Tracheal Intubation in Critically Ill Adults: A Secondary Analysis of 2 Randomized Trials. Journal of Intensive Care Medicine. 2022;37(7):899-907. doi: 10.1177/08850666211058646. PubMed PMID: WOS:000730362300001.

273. Venkatraman R, Hungerford JL, Hall MW, Moore-Clingenpeel M, Tobias JD. Dexmedetomidine for Sedation During Noninvasive Ventilation in Pediatric Patients. Pediatric Critical Care Medicine. 2017;18(9):831-7. doi: 10.1097/pcc.0000000000001226. PubMed PMID: WOS:000408818800008.

274. Vera M, Kattan E, Cerda T, Niklitshek J, Montaña R, Varas J, et al. Implementation of Distance-Based Simulation Training Programs for Healthcare Professionals Breaking Barriers During COVID-19 Pandemic. Simulation in Healthcare-Journal of the Society for Simulation in Healthcare. 2021;16(6):401-6. doi: 10.1097/sih.0000000000000550. PubMed PMID: WOS:000756772900025.

275. Verma A, Snehy A, Vishen A, Sheikh WR, Haldar M, Jaiswal S. Ketamine Use allows Noninvasive Ventilation in Distressed Patients with Acute Decompensated Heart Failure. Indian Journal of Critical Care Medicine. 2019;23(4):191-2. doi: 10.5005/jp-journals-10071-23153. PubMed PMID: WOS:000496508300011.

276. Vianello A, Turrin M, Guarnieri G, Molena B, Arcaro G, Turato C, et al. Prone Positioning Is Safe and May Reduce the Rate of Intubation in Selected COVID-19 Patients Receiving High-Flow Nasal Oxygen Therapy. Journal of Clinical Medicine. 2021;10(15). doi: 10.3390/jcm10153404. PubMed PMID: WOS:000682093200001.

277. Viscusi CD, Pacheco GS. Pediatric Emergency Noninvasive Ventilation. Emergency Medicine Clinics of North America. 2018;36(2):387-+. doi: 10.1016/j.emc.2017.12.007. PubMed PMID: WOS:000431097600009.

278. Vourc'h M, Asfar P, Volteau C, Bachoumas K, Clavieras N, Egreteau PY, et al. High-flow nasal cannula oxygen during endotracheal intubation in hypoxemic patients: a randomized controlled clinical trial. Intensive Care Medicine. 2015;41(9):1538-48. doi: 10.1007/s00134-015-3796-z. PubMed PMID: WOS:000360314900003.

279. Vourc'h M, Huard D, Feuillet F, Baud G, Guichoux A, Surbled M, et al. Preoxygenation in difficult airway management: high-flow oxygenation by nasal cannula versus face mask (the PREOPTIDAM study). Protocol for a single-centre randomised study. Bmj Open. 2019;9(4). doi: 10.1136/bmjopen-2018-025909. PubMed PMID: WOS:000471157200153.

280. Vukovic AA, Hanson HR, Murphy SL, Mercurio D, Sheedy CA, Arnold DH. Apneic oxygenation reduces hypoxemia during endotracheal intubation in the pediatric emergency department. American Journal of Emergency Medicine. 2019;37(1):27-32. doi: 10.1016/j.ajem.2018.04.039. PubMed PMID: WOS:000451306000005.

281. Waheed S, Kapadia NN, Khan MF, Kerai SM, Raheem A, Naeem R. Randomised controlled trial to assess the effectiveness of apnoeic oxygenation in adults using low-flow or high-flow nasal cannula with head side elevation versus usual care to prevent desaturation during endotracheal intubation in the emergency department (ApOxED): study protocol. Bmj Open. 2020;10(11). doi: 10.1136/bmjopen-2020-037964. PubMed PMID: WOS:000595442000005.

282. Wang ML, Hung MH, Chen JS, Hsu HH, Cheng YJ. Nasal high-flow oxygen therapy improves arterial oxygenation during one-lung ventilation in non-intubated thoracoscopic surgery. European Journal of Cardio-Thoracic Surgery. 2018;53(5):1001-6. doi: 10.1093/ejcts/ezx450. PubMed PMID: WOS:000432296700016.

283. Webb LV, Chahine R, Aban I, Prabhakaran P, Loberger JM. Predicting High-Flow Nasal Cannula Therapy Outcomes Using the ROX-HR Index in the Pediatric ICU. Respiratory Care. 2022;67(11):1377-84. doi: 10.4187/respcare.09765. PubMed PMID: WOS:000885343100002.

284. Wei W, Li X, Feng LL, Jiao JL, Li WX, Cai YR, et al. The effect of intraoperative transnasal humidified rapid-insufflation ventilatory exchange on emergence from general anesthesia in patients undergoing microlaryngeal surgery: a randomized controlled trial. Bmc Anesthesiology. 2023;23(1). doi: 10.1186/s12871-023-02169-y. PubMed PMID: WOS:001006131400001.

285. Wendel-Garcia PD, Mas A, González-Isern C, Ferrer R, Máñez R, Masclans JR, et al. Non-invasive oxygenation support in acutely hypoxemic COVID-19 patients admitted to the ICU: a multicenter observational retrospective study. Critical Care. 2022;26(1). doi: 10.1186/s13054-022-03905-5. PubMed PMID: WOS:000752921200001.

286. West JR, Scoccimarro A, Kramer C, Caputo ND. The effect of the apneic period on the respiratory physiology of patients undergoing intubation in the ED. American Journal of Emergency Medicine. 2017;35(9):1320-3. doi: 10.1016/j.ajem.2017.03.076. PubMed PMID: WOS:000407942600022.

287. Westergren V, Forsum U, Lundgren J. POSSIBLE ERRORS IN DIAGNOSIS OF BACTERIAL SINUSITIS IN TRACHEAL INTUBATED PATIENTS. Acta Anaesthesiologica Scandinavica. 1994;38(7):699-703. doi: 10.1111/j.1399-6576.1994.tb03980.x. PubMed PMID: WOS:A1994PP71900014.

288. Wetsch WA, Schroeder DC, Finke SR, Sander D, Ecker H, Böttiger BW, et al. A special oropharyngeal oxygenation device to facilitate apneic oxygenation in comparison to high flow oxygenation devices. Medical Gas Research. 2022;12(1):28-31. doi: 10.4103/2045-9912.323536. PubMed PMID: WOS:000693535100006.

289. Wetsch WA, Schroeder DC, Herff SJ, Böttiger BW, Wenzel V, Herff H. Identification of the Optimal Position of a Nasal Oxygen Cannula for Apneic Oxygenation: A Technical Simulation. Journal of Clinical Medicine. 2022;11(22). doi: 10.3390/jcm11226809. PubMed PMID: WOS:000887548800001.

290. Wilhelms SB, Wilhelms DB. Airway management procedures in Swedish emergency department patients-a national retrospective study. Bmc Emergency Medicine. 2022;22(1). doi: 10.1186/s12873-022-00627-3. PubMed PMID: WOS:000784561200001.

291. Wimalasena Y, Burns B, Reid C, Ware S, Habig K. Apneic Oxygenation Was Associated With Decreased Desaturation Rates During Rapid Sequence Intubation by an Australian Helicopter Emergency Medicine Service. Annals of Emergency Medicine. 2015;65(4):371-6. doi: 10.1016/j.annemergmed.2014.11.014. PubMed PMID: WOS:000352181200007.

292. Wong DT, Dallaire A, Singh KP, Madhusudan P, Jackson T, Singh M, et al. High-Flow Nasal Oxygen Improves Safe Apnea Time in Morbidly Obese Patients Undergoing General Anesthesia: A Randomized Controlled Trial. Anesthesia and Analgesia. 2019;129(4):1130-6. doi: 10.1213/ane.0000000000003966. PubMed PMID: WOS:000487076900035.

293. Wu YM, Li CC, Huang SY, Su YH, Wang CW, Chen JT, et al. A Comparison of Oxygenation Efficacy between High-Flow Nasal Cannulas and Standard Facemasks during Elective Tracheal Intubation for Patients with Obesity: A Randomized Controlled Trial. Journal of Clinical Medicine. 2022;11(6). doi: 10.3390/jcm11061700. PubMed PMID: WOS:000775151700001.

294. Xia JE, Zhang Y, Ni L, Chen L, Zhou CZ, Gao C, et al. High-Flow Nasal Oxygen in Coronavirus Disease 2019 Patients With Acute Hypoxemic Respiratory Failure: A Multicenter, Retrospective Cohort Study*. Critical Care Medicine. 2020;48(11):E1079-E86. doi: 10.1097/ccm.0000000000004558. PubMed PMID: WOS:000576935300011.

295. Yamagishi H, Wakatsuki Y, Tada T, Matsukura T. An air-locking port and high-flow nasal cannula in non-intubated uniportal video-assisted thoracic surgery for pneumothorax with pulmonary dysfunction: a case report. Surgical Case Reports. 2021;7(1). doi: 10.1186/s40792-021-01321-5. PubMed PMID: WOS:000711411700001.

296. Yan K, Lin J, Albaugh S, Yang M, Wang E, Cyberski T, et al. Measuring SARS-CoV-2 aerosolization in rooms of hospitalized patients. Laryngoscope Investigative Otolaryngology. 2022;7(4):1033-41. doi: 10.1002/lio2.802. PubMed PMID: WOS:000822976600001.

297. Yasuda H, Okano H, Mayumi T, Narita C, Onodera Y, Nakane M, et al. Post-extubation oxygenation strategies in acute respiratory failure: a systematic review and network meta-analysis. Critical Care. 2021;25(1). doi: 10.1186/s13054-021-03550-4. PubMed PMID: WOS:000639113600001.

298. Yi Y, Kim D, Choi EJ, Hong SB, Oh DK. The effect of a dedicated intensivist staffing to a medical emergency team on airway management in general wards. Medicine. 2024;103(25). doi: 10.1097/md.0000000000038571. PubMed PMID: WOS:001252260600051.

299. Zemach S, Helviz Y, Shitrit M, Friedman R, Levin PD. The Use of High-Flow Nasal Cannula Oxygen Outside the ICU. Respiratory Care. 2019;64(11):1333-42. doi: 10.4187/respcare.06611. PubMed PMID: WOS:000493310400003.

300. Zerbib O, Rattanachaiwong S, Palti N, Kagan I, Singer P. Energy and protein intake in critically ill people with respiratory failure treated by high-flow nasal-cannula oxygenation: An observational study. Nutrition. 2021;84. doi: 10.1016/j.nut.2020.111117. PubMed PMID: WOS:000633421800003.

301. Zhang C, Ou M. Comparison of hypoxemia, intubation procedure, and complications for non-invasive ventilation against high-flow nasal cannula oxygen therapy for patients with acute hypoxemic respiratory failure: a non-randomized retrospective analysis for effectiveness and safety (NIVaHIC-aHRF). Bmc Emergency Medicine. 2021;21(1). doi: 10.1186/s12873-021-00402-w. PubMed PMID: WOS:000607858300001.

302. Zhang WW, Yin HL, Xu YJ, Fang ZJ, Wang WL, Zhang C, et al. The effect of varying inhaled oxygen concentrations of high-flow nasal cannula oxygen therapy during gastroscopy with propofol sedation in elderly patients: a randomized controlled study. Bmc Anesthesiology. 2022;22(1). doi: 10.1186/s12871-022-01879-z. PubMed PMID: WOS:000878141100001.

303. Zhong M, Xia R, Zhou JY, Zhang J, Yi X, Yang AB. The comparison of preoxygenation methods before endotracheal intubation: a network meta-analysis of randomized trials. Frontiers in Medicine. 2024;11. doi: 10.3389/fmed.2024.1379369. PubMed PMID: WOS:001251713400001.

304. Zhou SQ, Zhou Y, Cao XH, Ni X, Du WJ, Xu ZD, et al. The efficacy of high flow nasal oxygenation for maintaining maternal oxygenation during rapid sequence induction in pregnancy A prospective randomised clinical trial. European Journal of Anaesthesiology. 2021;38(10):1052-8. doi: 10.1097/eja.0000000000001395. PubMed PMID: WOS:000693373000007.

305. Zhou XX, Luo XQ, Li Q, Chen GH, Tong J, Deng W. Prone versus lateral position in acute hypoxemic respiratory failure patients with HFNO therapy: study protocol for a multicentre randomised controlled open-label trial. Trials. 2023;24(1). doi: 10.1186/s13063-023-07761-8. PubMed PMID: WOS:001114811600008.

306. Zivanovic S, Scrivens A, Panza R, Reynolds P, Laforgia N, Ives KN, et al. Nasal High-Flow Therapy as Primary Respiratory Support for Preterm Infants without the Need for Rescue with Nasal Continuous Positive Airway Pressure. Neonatology. 2019;115(2):175-81. doi: 10.1159/000492930. PubMed PMID: WOS:000460983200011.

307. Zou L, Sun JK, Liu Y, Zhang WH, Jiang W, Yuan ST, et al. Surviving 2019 novel coronavirus pneumonia: A successful critical case report. Heart & Lung. 2020;49(6):692-5. doi: 10.1016/j.hrtlng.2020.08.009. PubMed PMID: WOS:000594532000007.
