# Supplementary material for: High-flow nasal cannula for pre- and apneic oxygenation during rapid sequence induction intubation in emergency surgery: A systematic review and meta-analysis
Source: PLoS One. 2025 Jan 24;20(1):e0316918. doi: 10.1371/journal.pone.0316918 (PMC11760591; doi:10.1371/journal.pone.0316918)
Supplement: S5 File — (DOCX) [file pone.0316918.s010.docx]

Pubmed (n=56)

(("intubation"[MeSH Terms] OR "intubation, intratracheal"[MeSH Terms] OR "rapid sequence induction and intubation"[MeSH Terms] OR "rapid sequence induction and intubation"[MeSH Terms] OR "rapid sequence intubation"[Title/Abstract] OR "rapid sequence induction"[Title/Abstract]) AND ("Cannula"[MeSH Terms] OR "Cannula"[MeSH Terms] OR "Cannula"[MeSH Terms] OR "high flow nasal cannula"[Title/Abstract] OR "high flow nasal cannula oxygen therapy"[Title/Abstract] OR "high flow nasal oxygen"[Title/Abstract] OR "transnasal humidified rapid insufflation ventilatory exchange"[Title/Abstract] OR "HFNC"[Title/Abstract] OR "HHFNC"[Title/Abstract] OR "THRIVE"[Title/Abstract])) AND ((randomizedcontrolledtrial[Filter]) AND (humans[Filter]))

Embase (n=319)

('high-flow nasal cannula'/exp OR 'high-flow nasal cannula' OR 'high-flow nasal cannula oxygen therapy' OR 'high-flow nasal oxygen' OR 'transnasal humidified rapid insufflation ventilatory exchange'/exp OR 'transnasal humidified rapid insufflation ventilatory exchange' OR 'hfnc' OR 'hhfnc' OR 'thrive'/exp OR 'thrive' OR 'hfno') AND ('rapid sequence induction' OR 'intubation' OR 'endotracheal intubation' OR 'rapid sequence intubation':ab,kw,ti OR 'intubation'/exp OR 'rapid sequence induction'/exp OR 'endotracheal intubation'/exp) AND 'randomized controlled trial'/de

SCOPUS (n=46)

TITLE-ABS-KEY ( ( "rapid sequence induction" OR "rapid sequence intubation" ) AND ( "high-flow nasal cannula" OR "high-flow nasal cannula oxygen therapy" OR "transnasal humidified rapid insufflation ventilatory exchange" OR "high-flow nasal oxygen" OR "nasal cannulate" OR "nasal cannula" ) ) AND ( LIMIT-TO ( DOCTYPE , "ar" ) )

Web of science (n=307)

1、((TS=(rapid sequence induction)) OR TS=(rapid sequence Intubation)) OR TS=(endotracheal intubation)

2、(((((TS=(oxygen nasal cannula)) OR TS=(humidified high flow nasal cannula therapy )) OR TS=(transnasal humidified rapid insufflation ventilatory exchange )) OR TS=(nasal cannula)) OR TS=(HFNC)) OR TS=(HFNO)

3、#2 AND #1 and Article (Document Types)
